# Supplementary material for: Single Cell Genetic Profiling of Tumors of Breast Cancer Patients Aged 50 Years and Older Reveals Enormous Intratumor Heterogeneity Independent of Individual Prognosis
Source: Cancers (Basel). 2021 Jul 5;13(13):3366. doi: 10.3390/cancers13133366 (PMC8267950; doi:10.3390/cancers13133366)
Supplement: Supplementary file 1 [file cancers-13-03366-s001.zip › cancers-1245840-SI/Supplementary_Files/Supplemental Figures/Supplemental Figure Legends.pdf]

Supplemental Figure Legends

Supplemental Figure S1.

Phylogenetic trees and color charts of miFISH analysis as well as results of image cytometry (if available) for all *long survival* cases except for 5L and 7L, which are shown in the main body of the manuscript (Figure 3 and 6). (A) case 1L, (B) case 2L, (C) case 3L, (D) case 4L, (E) case 6L, (F) case 8L, (G) case 9L, (H) case 10L, (I) case 11L, (J) case 12L, (K) case 13L, (L) case 14L, (M) case 15L, (N) case 16L, (O) case 17L, (P) case 18L, (Q) case 19L, (R) case 20L, (S) case 21L.

Image cytometry for selected cases: DNA histograms showing the quantitative measurements of the nuclear DNA content assessed by image cytometry using Feulgen-stained cytospins. For quantitative measurement of the DNA content the sample was screened for several diploid nuclei (granulocytes, lymphocytes) to set the 2c value indicating a diploid DNA content. The quantitative measurements of the nuclear DNA content (x axis) of the tumor cells given in “c” units were then calculated accordingly [18]. The y axis represents the total cell count: a minimum of 1214 nuclei for each case (mean, 6700; range, 1214 to 16,425) were analyzed.

Phylogenetic trees: The construction of the FISHtrees was done using phylogenetic algorithms (software FISHtrees 3.2) in the ploidyless mode. FISH patterns are depicted in the following gene order *COX2*, *DBC2*, *MYC*, *CCND1*, *CDH1*, *TP53*, *HER2*, *ZNF217*. The FISHtrees results show the clonal evolution by generating a tree model starting from a normal state root (2-2-2-2-2-2-2-2) continuing by heuristically seeking to minimize the total number of CNAs across the tree. The size of the nodes reflects, but is not proportional to, the frequency of the patterns in the cell population. Nodes encircled by a solid line reflects miFISH-signal-patterns observed in the tumor sample. FISHtree algorithm predicts transit signal-patterns that are not observed in the sample so that in the evolution tree generated by the algorithm the up and downstream nodes can be linked. Those transit patterns are represented by nodes encircled with a dashed line.

Color charts: Color chart of miFISH analysis with eight gene-specific probes. Copy number counts for each nucleus are displayed as gains (green), losses (red) and neutral (blue). Gene-specific miFISH markers are plotted vertically with the “Locus” column depicting the specific chromosome arm for each probe on the left of the plot, and the corresponding gene name on the right.

Nuclei are arranged horizontally by the frequency of signal patterns from left to right. Each vertical line discerns specific gain-and-loss patterns and the prevalence of the cell clone in the tumor population. Copy number gains and losses are depicted as percentages of the total cell population in the “Gain” and “Loss” column of the table on the right. Color-labeled percentages indicate a threshold of 15%. The column “AvgSig” refers to the average of all signal numbers specified for each of the eight analyzed gene probes. Orange labeled AvgSig-values indicate, that the threshold value of 15% of all nuclei was reached for both a detected copy number gain and loss in the respective gene probe.

Supplemental Figure S2.

Phylogenetic trees and color charts of miFISH analysis as well as results of image cytometry (if available) for all *short survival* cases except for case 13S (see Figure 2A and Supplemental Figure S6A,B). Also, the color charts and DNA histograms of case 4S and 8S are shown in the main body of the manuscript (Figure 4). (A) case 1S, (B) case 2S, (C) case 3S, (D) case 4S, (E) case 5S, (F) case 6S, (G) case 7S, (H) case 8S, (I) case 9S, (J) case 10S, (K) case 11S, (L) case 12S, (M) case 14S, (N) case 15S, (O) case 16S, (P) case 17S, (Q) case 18S.

Image cytometry for selected cases: DNA histograms showing the quantitative measurements of the nuclear DNA content assessed by image cytometry using Feulgen-stained cytospins. For quantitative measurement of the DNA content the sample was screened for several diploid nuclei (granulocytes, lymphocytes) to set the 2c value indicating a diploid DNA content. The quantitative measurements of the nuclear DNA content (x axis) of the tumor cells given in “c” units were then calculated accordingly [18]. The y axis represents the total cell count: a minimum of 1214 nuclei for each case (mean, 6700; range, 1214 to 16,425) were analyzed.

Consensus phylogenetic trees: The construction of the FISHtrees was done using phylogenetic algorithms (software FISHtrees 3.2) in the ploidyless mode. FISH patterns are depicted in the following gene order *COX2*, *DBC2*, *MYC*, *CCND1*, *CDH1*, *TP53*, *HER2*, *ZNF217*. The FISHtrees results show the clonal evolution by generating a tree model starting from a normal state root (2-2-2-2-2-2-2-2) continuing by heuristically seeking to minimize the total number of CNAs across the tree.

The size of the nodes reflects, but is not proportional to, the frequency of the patterns in the cell population. Nodes encircled by a solid line reflects miFISH-signal-patterns

observed in the tumor sample. The FISH trees algorithm predicts transit signal-patterns that are not observed in the sample so that in the evolution tree generated by the algorithm the up and downstream nodes can be linked. Those transit patterns are represented by nodes encircled with a dashed line. Details of the analysis are described in Materials and Methods.

Color charts: Color chart of miFISH analysis with eight gene-specific probes. Copy number counts for each nucleus are displayed as gains (green), losses (red) and neutral (blue). Gene-specific miFISH markers are plotted vertically with the “Locus” column depicting the specific chromosome arm for each probe on the left of the plot, and the corresponding gene name on the right. Nuclei are arranged horizontally by the frequency of signal patterns from left to right. Each vertical line discerns specific gain-and-loss patterns and the prevalence of the cell clone in the tumor population. Copy number gains and losses are depicted as percentages of the total cell population in the “Gain” and “Loss” column of the table on the right. Color-labeled percentages indicate a threshold of 15%. The column “AvgSig” refers to the average of all signal numbers specified for each of the eight analyzed gene probes. Orange labeled AvgSig-values indicate that the threshold value of 15% of all nuclei was reached for both a detected copy number gain and loss in the respective gene probe..

Supplemental Figure S3.

Clinicopathological features, NGS-mutation-analysis and miFISH results presented for the entire cohort (*n*=39) with corresponding color codes sorted by survival time after diagnosis and separated into the groups diploid and aneuploid.

(A) Summary of the clinicopathological features (rows), plotted per individual sample (columns).

(B) Mutation analysis of 563 breast-cancer associated genes (OncoVar) by NGS. Distribution of mutations sorted by chromosomal location that affected genes in at least three samples. The color code indicates the type of mutation. *PIK3CA*, *TP53* and *MAP3K1* were the most frequently mutated genes.

(C) Copy number alterations of 8 breast-cancer related genes sorted by chromosomal location were identified by miFISH and are plotted vertically per individual sample (columns). Green color indicates a gain, red color a loss. We chose dark hues if the majority (≥ 85%) of all nuclei showed a gain/loss and light hues if 15-84% showed a

gain/loss. The oncogenes *COX2* and *MYC* and the tumor suppressor genes *CDH1* and *TP53* were the most frequently altered genes.

Supplemental Figure S4.

Clinicopathological features, NGS-mutation-analysis and miFISH results presented for the entire cohort (*n*=39) with corresponding color codes sorted by ploidy and separated into the groups low instability index versus high instability index.

(A) Summary of the clinicopathological features (rows), plotted per individual sample (columns).

(B) Mutation analysis of 563 breast-cancer associated genes (OncoVar) by NGS. Distribution of mutations sorted by chromosomal location that affected genes in at least three samples. The color code indicates the type of mutation. *PIK3CA*, *TP53* and *MAP3K1* were the most frequently mutated genes.

(C) Copy number alterations of 8 breast-cancer related genes sorted by chromosomal location were identified by miFISH and are plotted vertically per individual sample (columns). Green color indicates a gain, red color a loss. We chose dark hues if the majority (≥ 85%) of all nuclei showed a gain/loss and light hues if 15-84% showed a gain/loss. The oncogenes *COX2* and *MYC* and the tumor suppressor genes *CDH1* and *TP53* were the most frequently altered genes.

Supplemental Figure S5.

Lolliplot chart of the mutation sites in *PIK3CA* (A) and *TP53* (B).

(A) Mutations are mapped on the linear PIK3CA protein and its domains from Pfam (Protein family database from Wellcome Trust Sanger Institute; lollipop plot). The different domains of PIK3CA are marked in color: PI3-kinase family, p85-binding domain (32–108) in green, PI3-kinase family, ras-binding domain (174–291) in red, Phosphoinositide 3-kinase C2 (351–483) in blue, Phosphoinositide 3-kinase family, accessory domain (PIK domain) (520–703) in yellow, and Phosphatidylinositol 3- and 4-kinase (798–1014) in purple.

The amount of cases showing a mutation at the same location is presented by the height of the ‘lollipops‘ (y-axis). ‘Lollipops‘: green, missense mutation; black, truncating mutation.

(B) Mutations are mapped on the linear TP53 protein and its domains from Pfam (Protein family database from Wellcome Trust Sanger Institute; lollipop plot). The different domains of TP53 are marked in color: TP53 transactivation motif (5–29) in green, TP53 DNA binding domain (95–289) in red and TP53 tetramerization motif (319–358) in blue. The amount of cases showing a mutation at the same location is presented by the height of the 'lollipops' (y-axis). 'Lollipops': green, missense mutation; black, truncating mutation.

Supplemental Figure S6.

Phylogenetic tree (A) and color chart (C) of miFISH results as well as DNA histogram (B) for case 13S.

(A) Consensus phylogenetic tree of miFISH results with the eight described gene probes: The construction of the FISHtrees was done using phylogenetic algorithms (software FISHtrees 3.2) in the ploidyless mode. FISH patterns are depicted in the following gene order *COX2*, *DBC2*, *MYC*, *CCND1*, *CDH1*, *TP53*, *HER2*, *ZNF217*. The FISHtrees results show the clonal evolution by generating a tree model starting from a normal state root (2-2-2-2-2-2-2-2) continuing by heuristically seeking to minimize the total number of CNAs across the tree. The size of the nodes reflects, but is not proportional to, the frequency of the patterns in the cell population. Nodes encircled by a solid line reflects miFISH-signal-patterns observed in the tumor sample. The FISH trees algorithm predicts transit signal-patterns that are not observed in the sample so that in the evolution tree generated by the algorithm the up and downstream nodes can be linked. Those transit patterns are represented by nodes encircled with a dashed line. Details of the analysis are described in Materials and Methods.

(B) DNA histogram showing the quantitative measurements of the nuclear DNA content assessed by image cytometry using Feulgen-stained cytospins. For quantitative measurement of the DNA content the sample was screened for several diploid nuclei (granulocytes, lymphocytes) to set the 2c value indicating a diploid DNA content.

The quantitative measurements of the nuclear DNA content (x axis) of the tumor cells given in "c" units were then calculated accordingly [18]. The y axis represents the total cell count: in case 13S 1,381 nuclei were analyzed.

(C) Color chart of miFISH analysis with 25 gene probes: centromere probes CCP2, 3, 4, 6, 7, 9, 10, 11, 12, 15, 18, X and locus-specific probes *COX2*, *CCNB1*, *DBC2*, *MYC*,

CCND1, *RB1*, *CDH1*, *TP53*, *HER2*, *CCNE1*, *ZNF217*, *DSCR8*, *NF2,* as described in Materials and Methods. Copy number counts for each nucleus are displayed as gains (green), losses (red) and neutral (blue). Gene-specific miFISH markers are plotted vertically with the “Locus” column depicting the specific chromosome arm for each probe on the left of the plot, and the corresponding gene name on the right. Nuclei are arranged horizontally by the frequency of signal patterns from left to right. Each vertical line discerns specific gain-and-loss patterns and the prevalence of the cell clone in the tumor population. Copy number gains and losses are depicted as percentages of the total cell population in the “Gain” and “Loss” column of the table on the right. Color-labeled percentages indicate a threshold of 15%. The column “AvgSig” refers to the average of all signal numbers specified for each of the 25 analyzed gene probes. Notice the eye-catching several copy number losses leading to the postulation of a severe hypodiploid tumor sample.

Supplemental Figure S7.

Results of the FISHtree analysis presenting the maximum FISHtree depth and total number of events in the tree of different subgroups.

(A,B,C) Total number of events in the FISHtrees including minimum, maximum, median and outliners are presented as a boxplot for each subgroup: *(*A) long and short survival*,* (B) diploid versus aneuploid tumors and (C) tumors with low versus high instability index. Note the significant difference between diploid versus aneuploid tumors ($p=0.0008$) and low versus high instability index ($p\leq0.0001$).

(C,D,E) FISHtree depth including minimum, maximum, median and outliners are presented as a boxplot for each subgroup: (C) long and short survival*,* (D) diploid versus aneuploid tumors and (E) tumors with low versus high instability index. Note the significant difference between diploid versus aneuploid tumors ($p=0.0008$) and tumors with low versus high instability index ($p\leq0.0001$).

Supplemental Figure S8.

Visualization of mutual exclusivity and co-occurrence analysis results of mutations in *TP53* and *PIK3CA* (NGS results) and copy number alterations (miFISH results) using the Mutual Exclusivity Modules in Cancer (MeMo) algorithm [40,41] as described in Materials and Methods. Results are plotted vertically per individual sample (columns) for the entire cohort (n=39) differently sorted by their significant correlations (upper two

genes). (A) co-occurrence of *DBC2* and *MYC*, (B) co-occurrence of *TP53* and *HER2*, (C) co-occurrence of *DBC2* and *HER2*, (D) mutual exclusivity of *PIK3CA* and *HER2* and (E) mutual exclusivity of *PIK3CA* and *CCND1*.

Copy number alterations are displayed as colored bars, mutations are marked as horizontal lines in bars. The grey-colored bars represent samples without alterations in the analyzed genes.
