# Supplementary material for: Single Cell Genetic Profiling of Tumors of Breast Cancer Patients Aged 50 Years and Older Reveals Enormous Intratumor Heterogeneity Independent of Individual Prognosis
Source: Cancers (Basel). 2021 Jul 5;13(13):3366. doi: 10.3390/cancers13133366 (PMC8267950; doi:10.3390/cancers13133366)
Supplement: Supplementary file 1 [file cancers-13-03366-s001.zip › cancers-1245840-SI/Supplementary_Files/Supplemental Figures/Supplemental Figure S1.pdf]

A

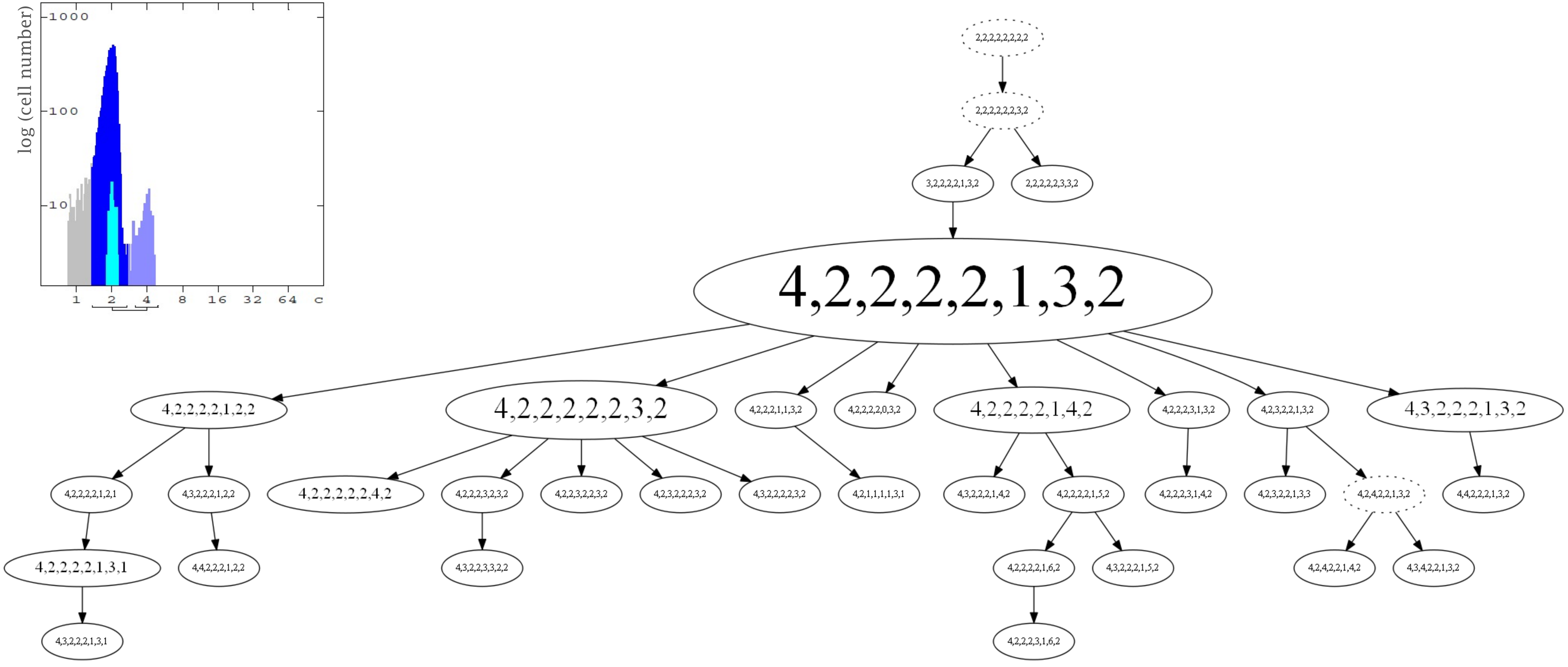

1L

Locus	250 nuclei					Instability Index: 13.2	Average ploidy: 2.0			
	74,4%	8,4%	4,8%	2	2	8,4%	GENE	GAIN	LOSS	AvgSig
1q							COX2	100%	0%	4,0
8p							DBC2	7%	0%	2,1
8q							MYC	2%	0%	2,0
11q							CCND1	0%	0%	2,0
16q							CDH1	2%	1%	2,0
17p							TP53	1%	89%	1,1
17q							HER2	96%	0%	3,1
20q							ZNF217	0%	3%	2,0

B

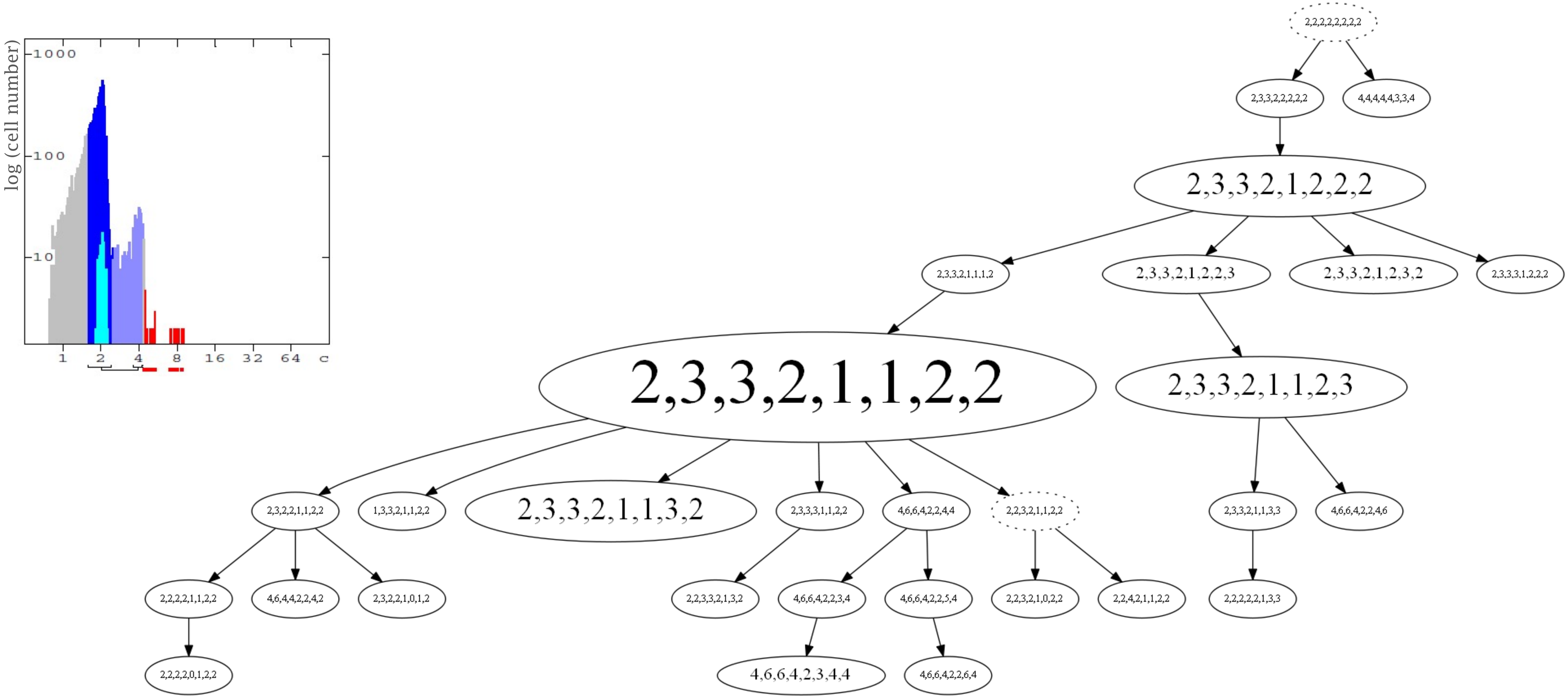

2L	Locus	250 nuclei				Instability Index: 11.2				Average ploidy: 2.1
		67,6%	8,4%	7,6%	5,6%	10,8%	GENE	GAIN	LOSS	AvgSig
	1q						COX2	0%	1%	2,1
	8p						DBC2	97%	0%	3,1
	8q						MYC	97%	0%	3,1
	11q						CCND1	1%	0%	2,1
	16q						CDH1	0%	98%	1,1
	17p						TP53	0%	89%	1,2
	17q						HER2	11%	2%	2,2
	20q						ZNF217	8%	0%	2,2

C

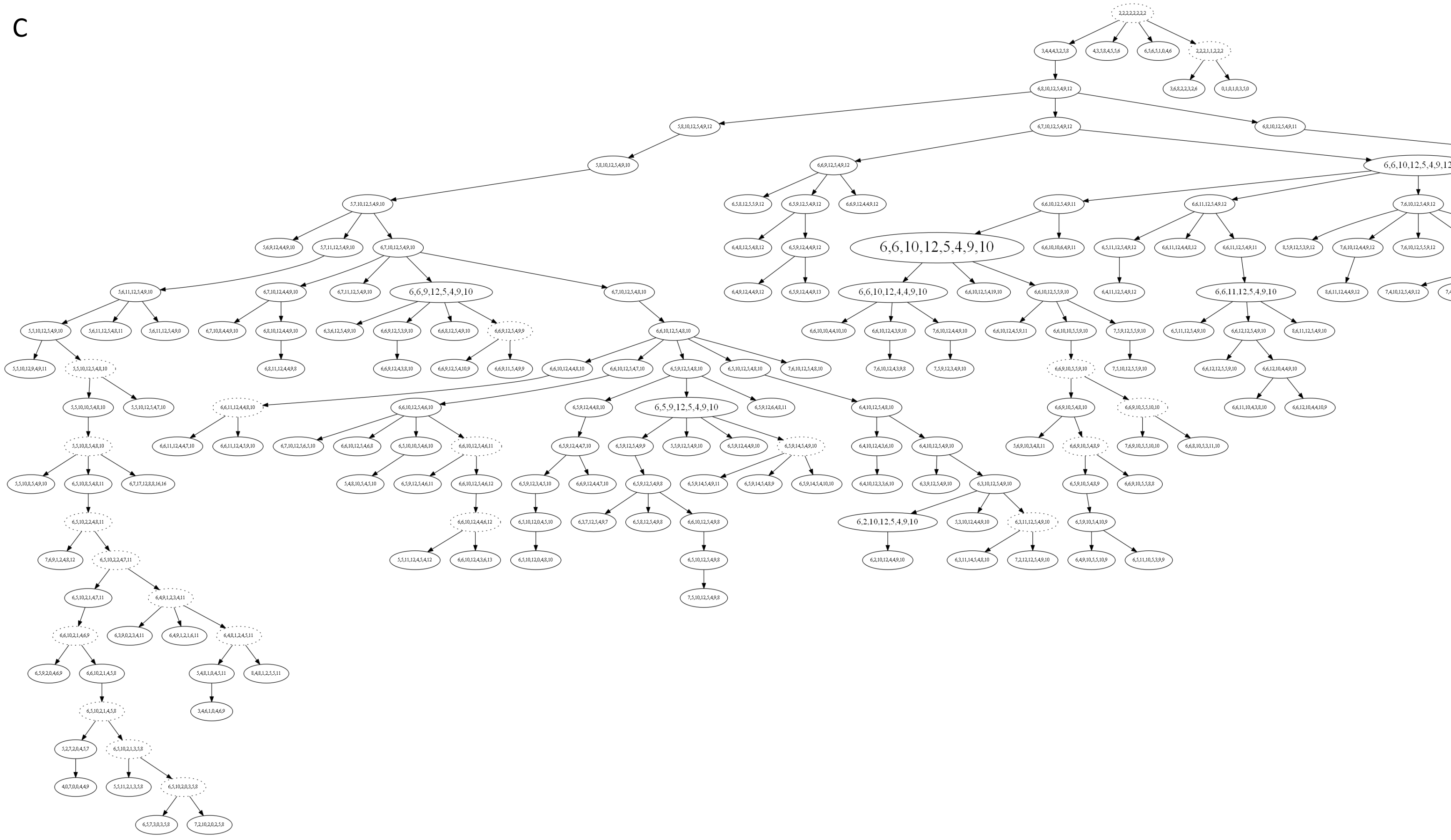

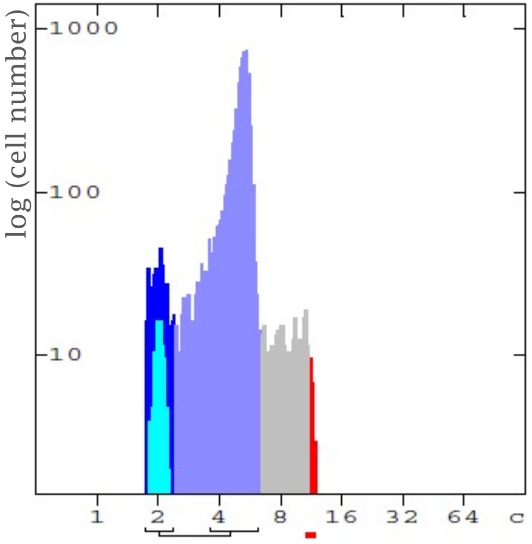

	Locus	250 nuclei											Instability Index: 72.4				Average ploidy: 4.6
3L		23,6%	14,0%	9,6%	8,8%	8,0%	4,0%	3,6%	3,6%	2,8%	2,4	2,4	17,2%	GENE	GAIN	LOSS	AvgSig
	1q													COX2	92%	1%	6,0
	8p													DBC2	62%	11%	5,3
	8q													MYC	99%	0%	9,7
	11q													CCND1	93%	6%	11,1
	16q													CDH1	20%	22%	4,5
	17p													TP53	3%	62%	4,0
	17q													HER2	97%	0%	8,5
	20q													ZNF217	99%	1%	10,3

D

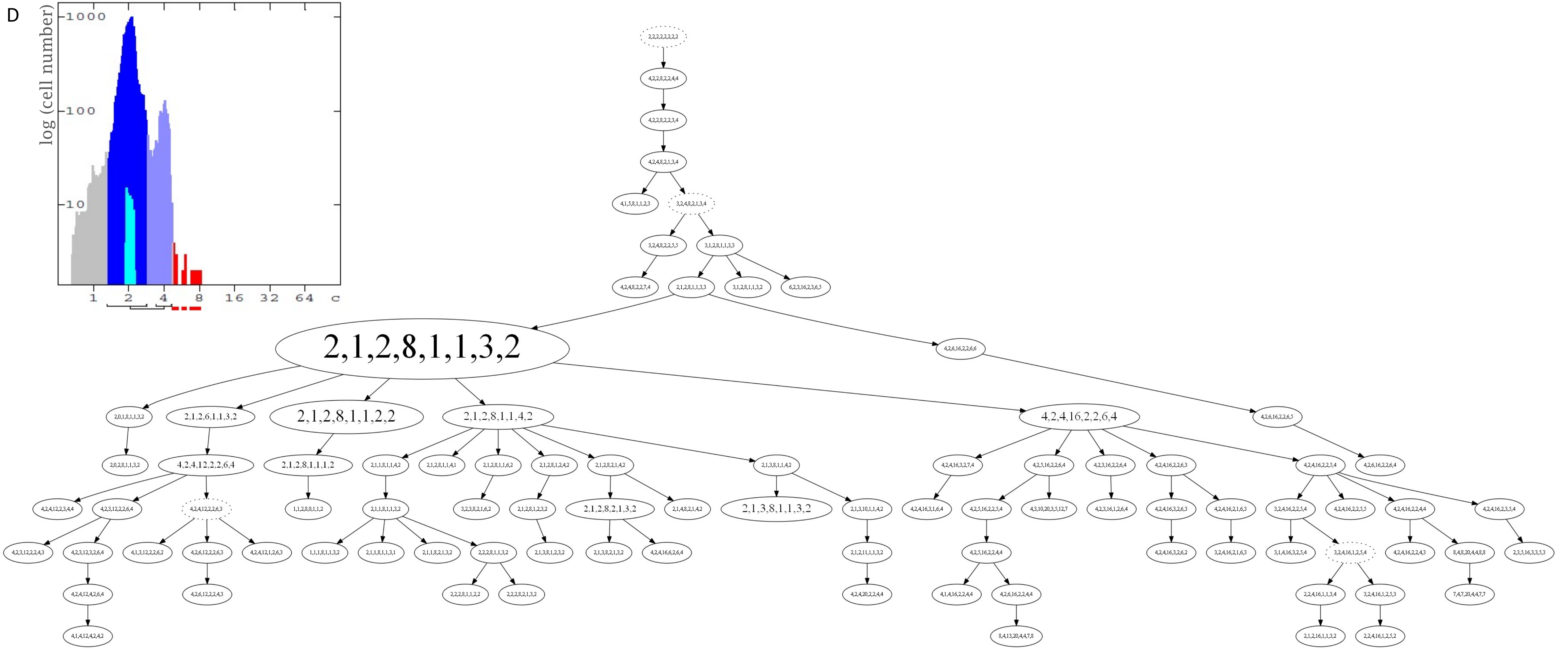

4L	Locus	250 nuclei							Instability Index: 34.8				Average ploidy: 2.6
		55,2%	10,8%	6,4%	2,8%	2,4	2,4	2,4	17,6%	GENE	GAIN	LOSS	AvgSig
	1q									COX2	1%	5%	2,6
	8p									DBC2	0%	98%	1,3
	8q									MYC	12%	7%	2,7
	11q									CCND1	100%	0%	10,1
	16q									CDH1	0%	95%	1,4
	17p									TP53	0%	98%	1,3
	17q									HER2	81%	5%	3,6
	20q									ZNF217	4%	8%	2,6

E

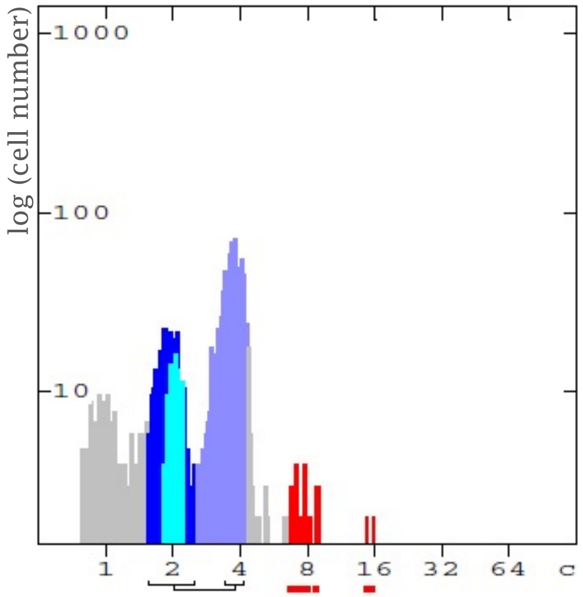

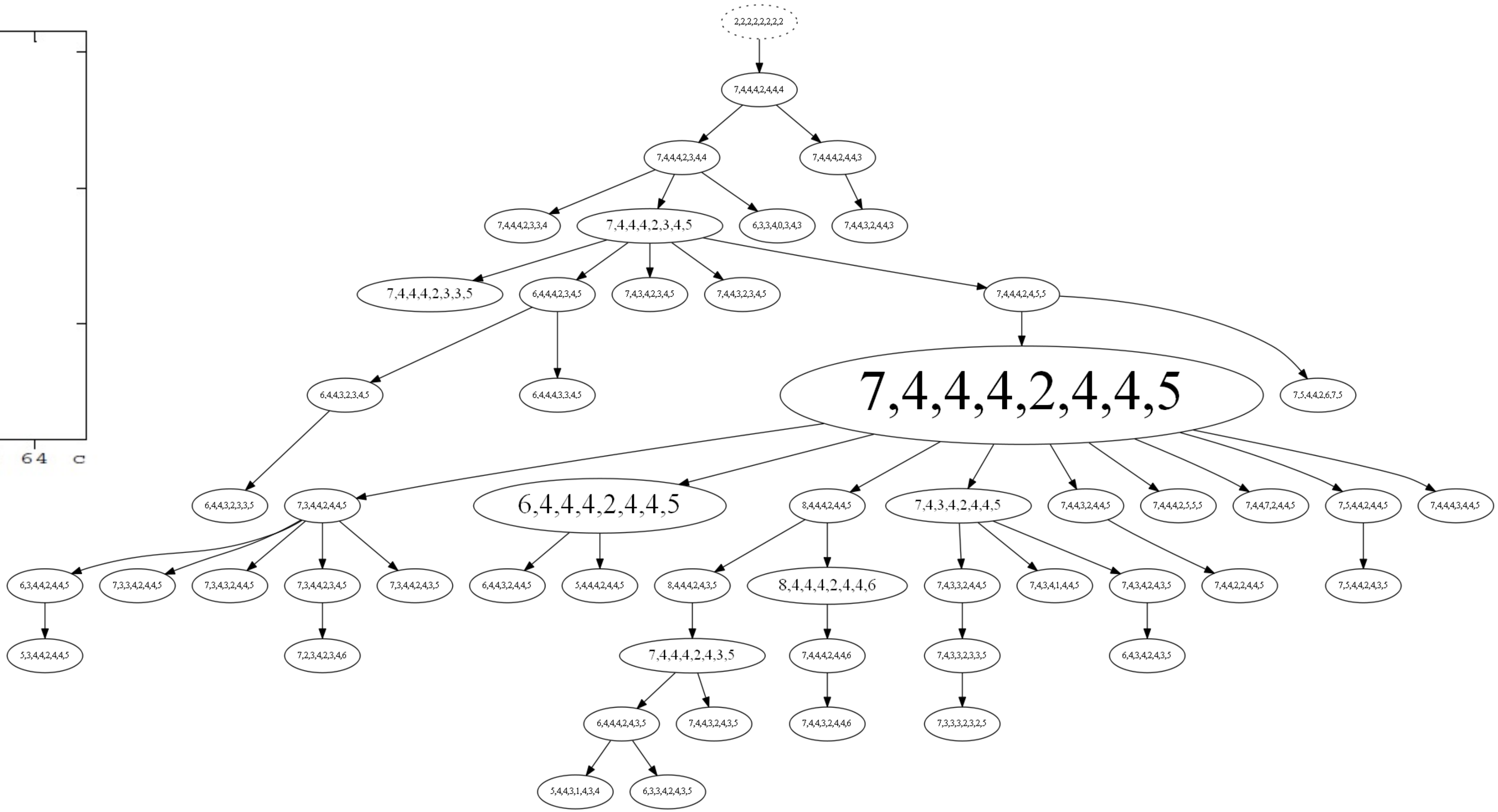

	Locus	250 nuclei					Instability Index: 20.8				Average ploidy: 4,0
6L		76,8%	2,4	2,4	2,0	2,0	14,4%	GENE	GAIN	LOSS	AvgSig
	1q							COX2	100%	0%	6,9
	8p							DBC2	1%	4%	4,0
	8q							MYC	0%	6%	3,9
	11q							CCND1	1%	6%	3,9
	16q							CDH1	0%	100%	2,0
	17p							TP53	1%	7%	3,9
	17q							HER2	2%	7%	3,9
	20q							ZNF217	97%	1%	5,0

F

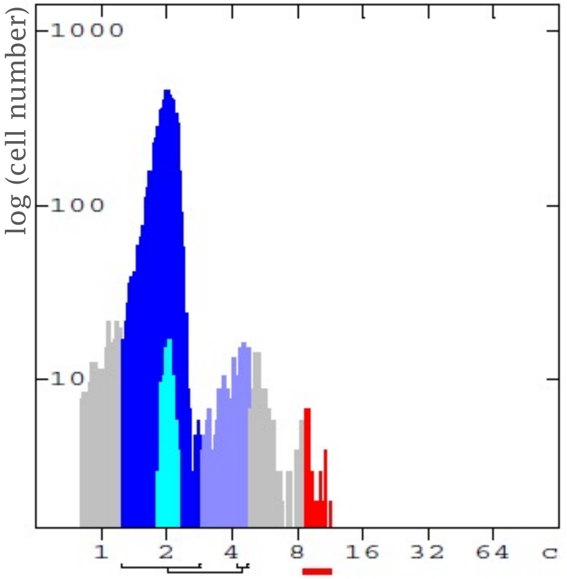

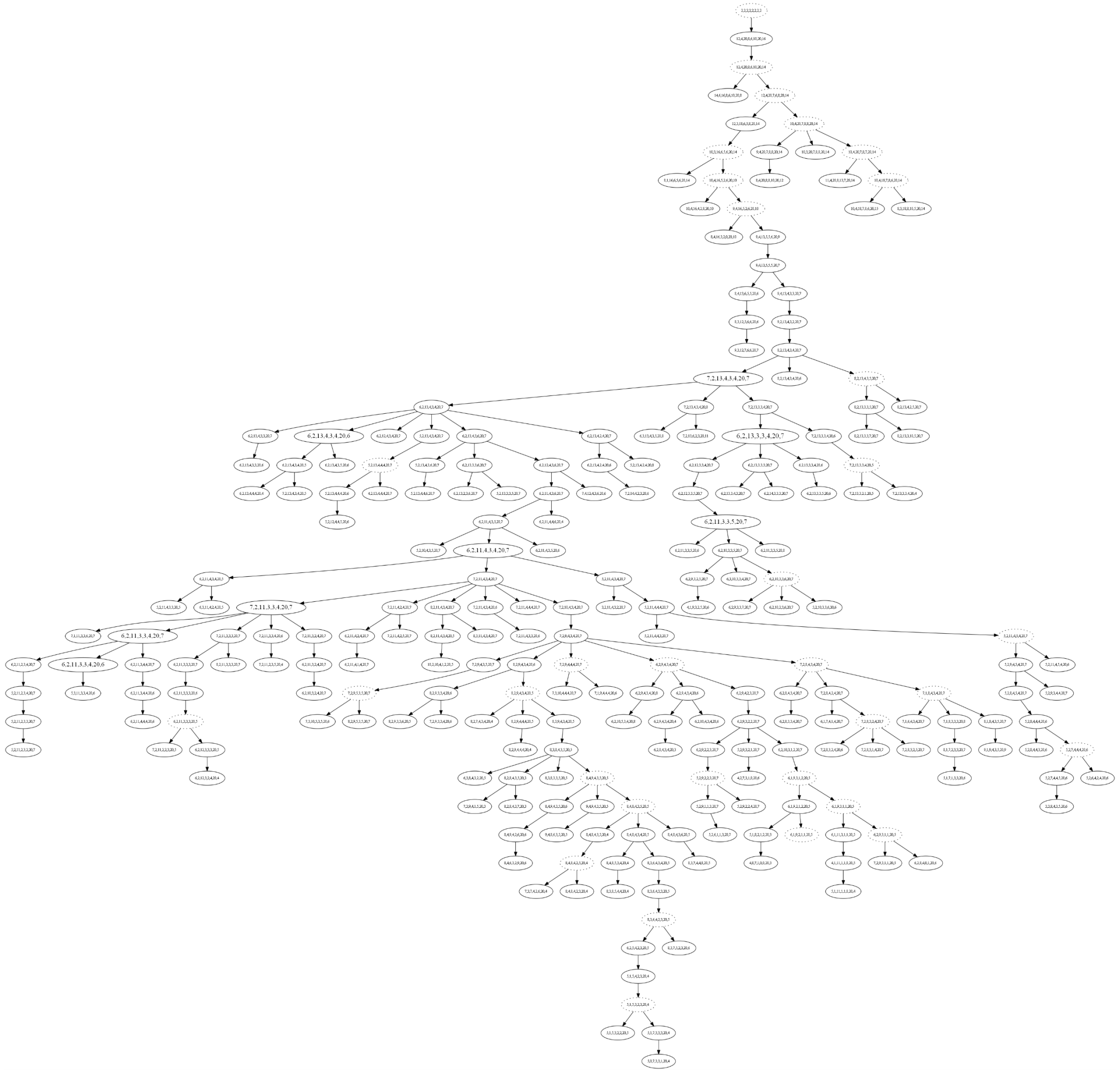

	Locus	250 nuclei												Instability Index: 86.4	Average ploidy: 3.2			
		22,8%	19,2%	8,4%	4,8%	4,4%	4,0%	3,2%	3,2%	2,8%	2,8%	2,0	2,0	20,4%	GENE	GAIN	LOSS	AvgSig
T8	1q														COX2	99%	0%	6,6
	8p														DBC2	5%	86%	2,2
	8q														MYC	100%	0%	10,9
	11q														CCND1	56%	8%	3,7
	16q														CDH1	14%	22%	3,1
	17p														TP53	76%	9%	4,2
	17q														HER2	100%	0%	56,9
	20q														ZNF217	98%	0%	6,6

G

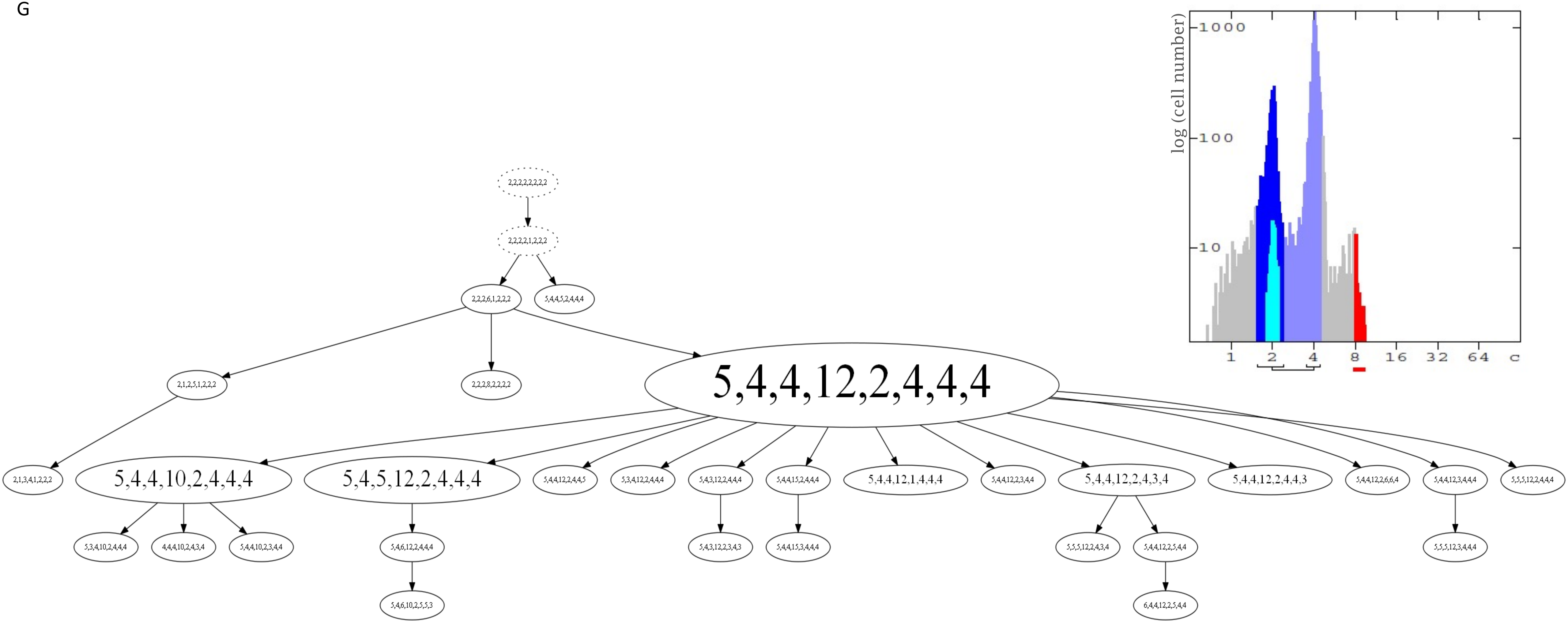

	Locus	250 nuclei			Instability Index: 12.0				Average ploidy: 4,0
		81,6%	6,0%	2,8%	9,6%	GENE	GAIN	LOSS	AvgSig
T6	1q					COX2	98%	0%	5,0
	8p					DBC2	2%	2%	4,0
	8q					MYC	8%	1%	4,1
	11q					CCND1	100%	0%	11,8
	16q					CDH1	0%	100%	2,0
	17p					TP53	2%	1%	4,0
	17q					HER2	1%	4%	3,9
	20q					ZNF217	0%	2%	4,0

H

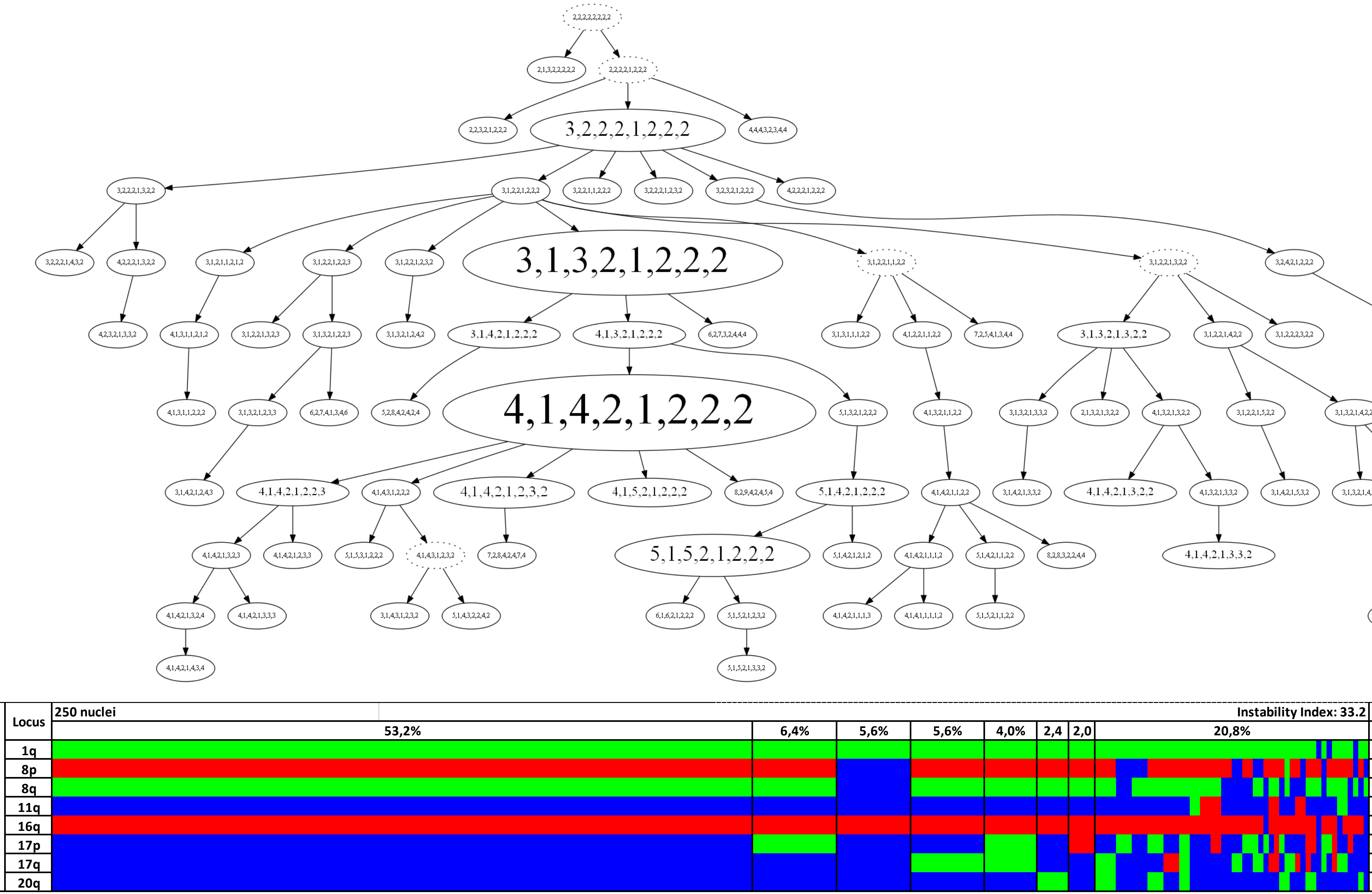

Average ploidy: 2.1

GENE	GAIN	LOSS	AvgSig
COX2	99%	0%	3,8
DBC2	0%	88%	1,2
MYC	88%	0%	3,7
CCND1	2%	3%	2,0
CDH1	0%	98%	1,0
TP53	16%	5%	2,2
HER2	16%	3%	2,2
ZNF217	8%	0%	2,2

I

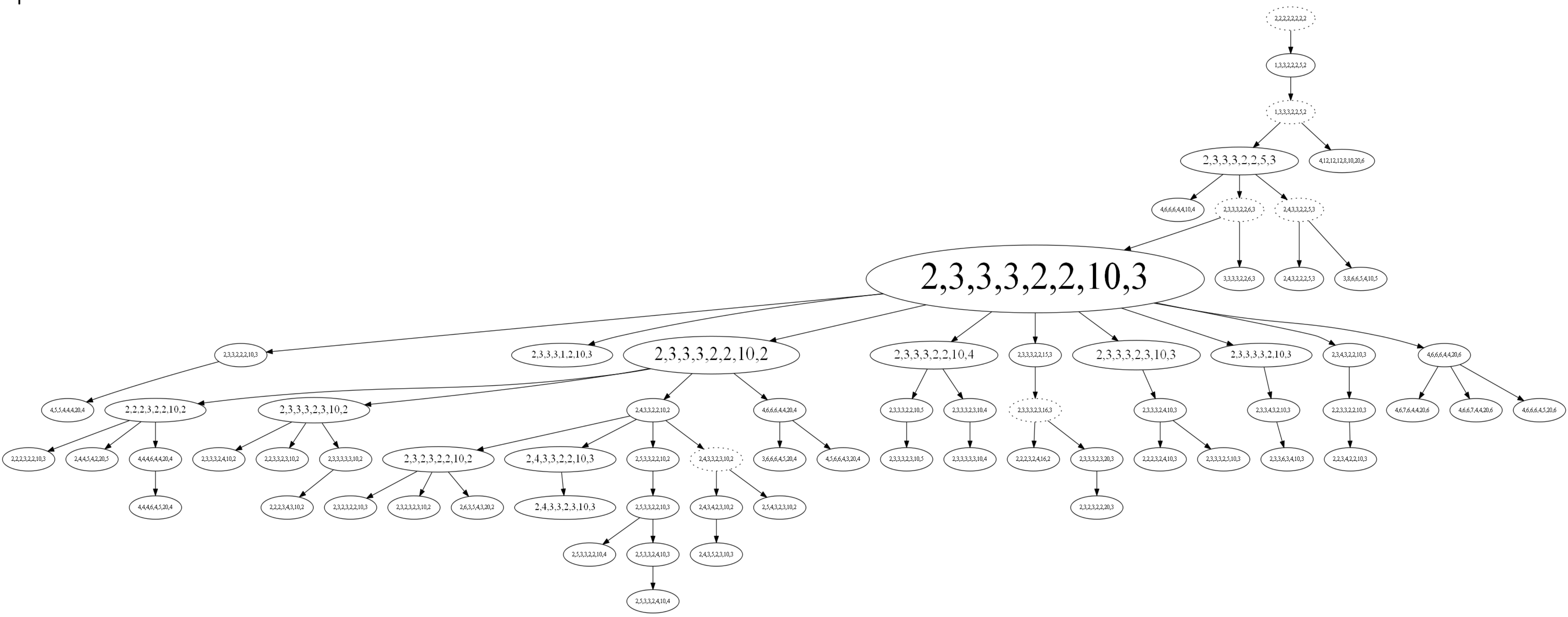

11L	Locus	250 nuclei							Instability Index: 25.6				Average ploidy: 2.1
		59,6%	10,0%	8,8%	3,6%	2,4	2,0	2,0	11,6%	GENE	GAIN	LOSS	AvgSig
	1q									COX2	0%	2%	2,1
	8p									DBC2	94%	0%	3,3
	8q									MYC	92%	0%	3,1
	11q									CCND1	98%	0%	3,2
	16q									CDH1	5%	1%	2,2
	17p									TP53	19%	0%	2,4
	17q									HER2	100%	0%	10,6
	20q									ZNF217	78%	0%	3,0

J

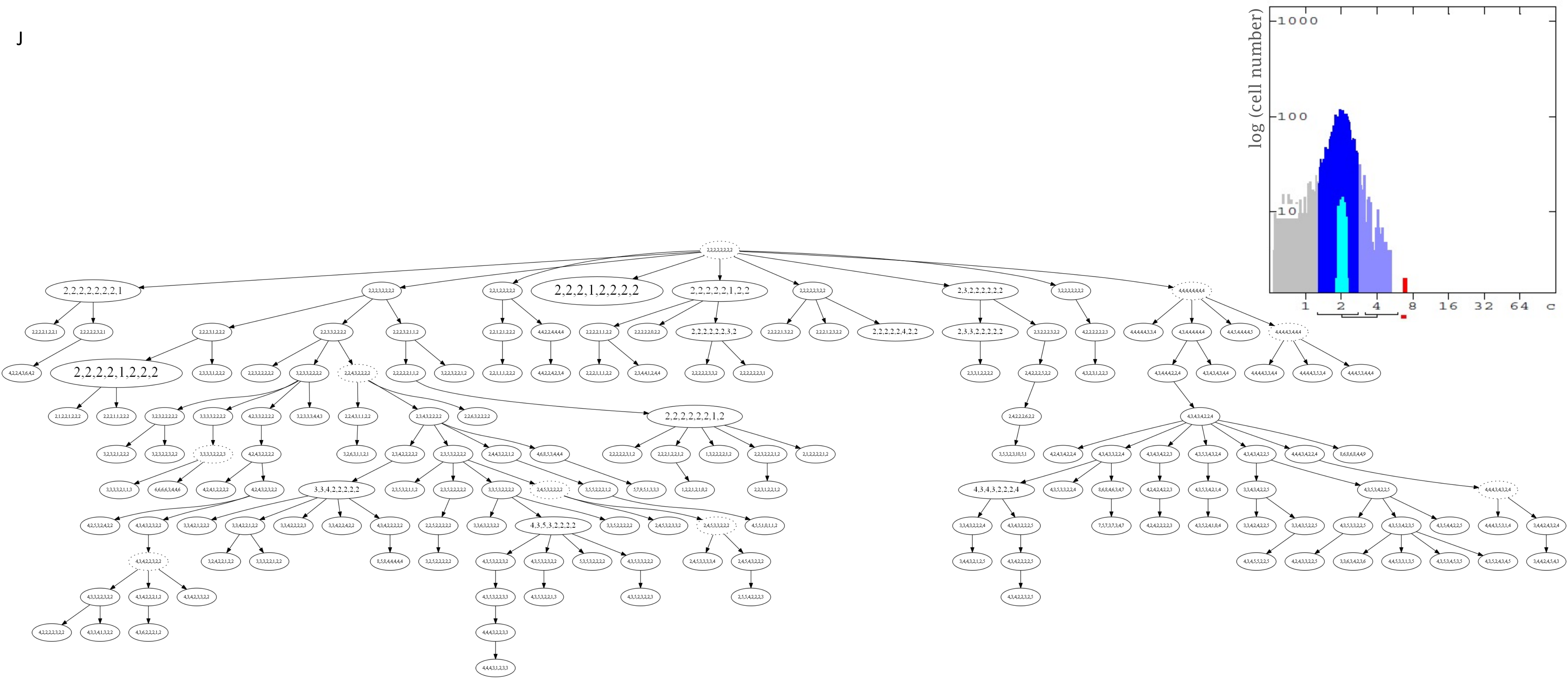

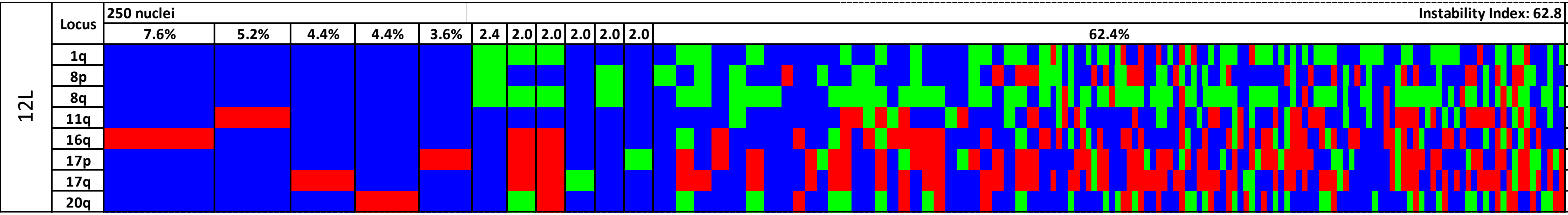

Average ploidy: 2.512

GENE	GAIN	LOSS	AvgSig
COX2	32%	4%	2.9
DBC2	22%	10%	2.7
MYC	48%	3%	3.3
CCND1	11%	21%	2.4
CDH1	8%	33%	2.2
TP53	10%	36%	2.3
HER2	5%	40%	2.1
ZNF217	18%	17%	2.6

K

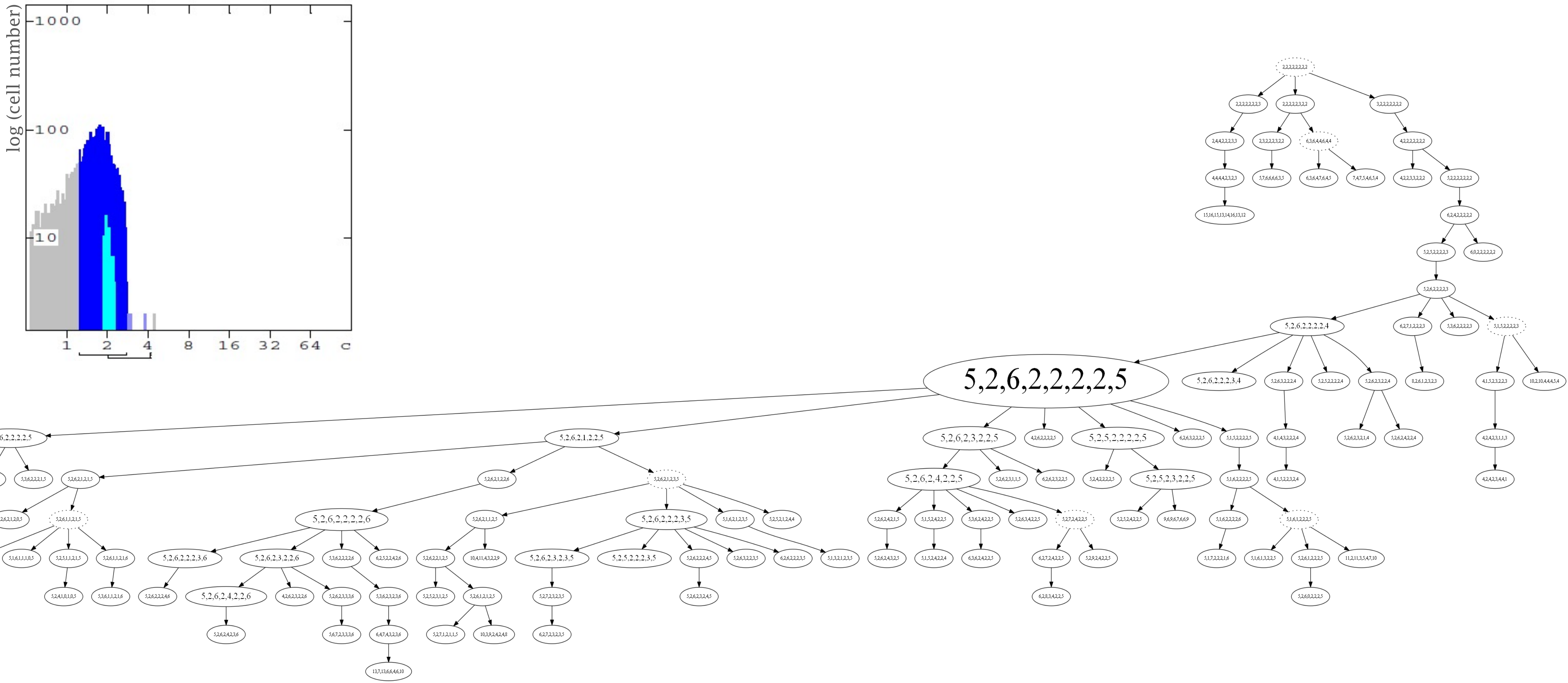

13L

Locus	250 nuclei							Instability Index: 44.8	Average ploidy: 3.084			
	42,8%	12,8%	9,2%	7,6%	2,8%	2,4	2,0	20,4%	GENE	GAIN	LOSS	AvgSig
1q									COX2	98%	1%	5,1
8p									DBC2	3%	88%	2,2
8q									MYC	95%	0%	5,8
11q									CCND1	2%	91%	2,1
16q									CDH1	14%	64%	2,5
17p									TP53	2%	90%	2,2
17q									HER2	4%	78%	2,3
20q									ZNF217	90%	2%	5,0

L

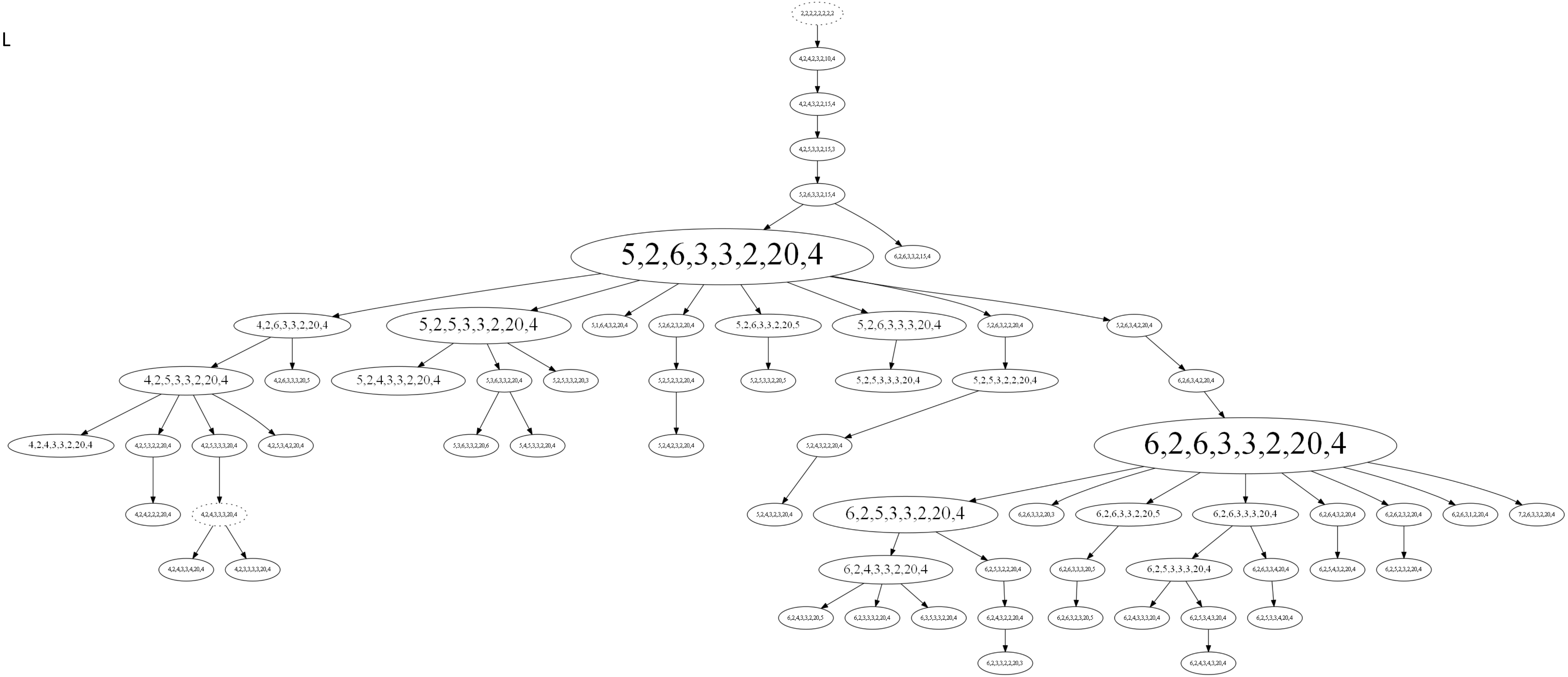

14L	Locus	250 nuclei				Instability Index: 26.4				Average ploidy: 3.0
		71,6%	10,8%	4,0%	2,8%	10,8%	GENE	GAIN	LOSS	AvgSig
	1q						COX2	100%	0%	5,3
	8p						DBC2	0%	98%	2,0
	8q						MYC	99%	0%	5,3
	11q						CCND1	1%	3%	3,0
	16q						CDH1	2%	6%	3,0
	17p						TP53	2%	85%	2,2
	17q						HER2	100%	0%	30,2
	20q						ZNF217	98%	0%	4,0

M

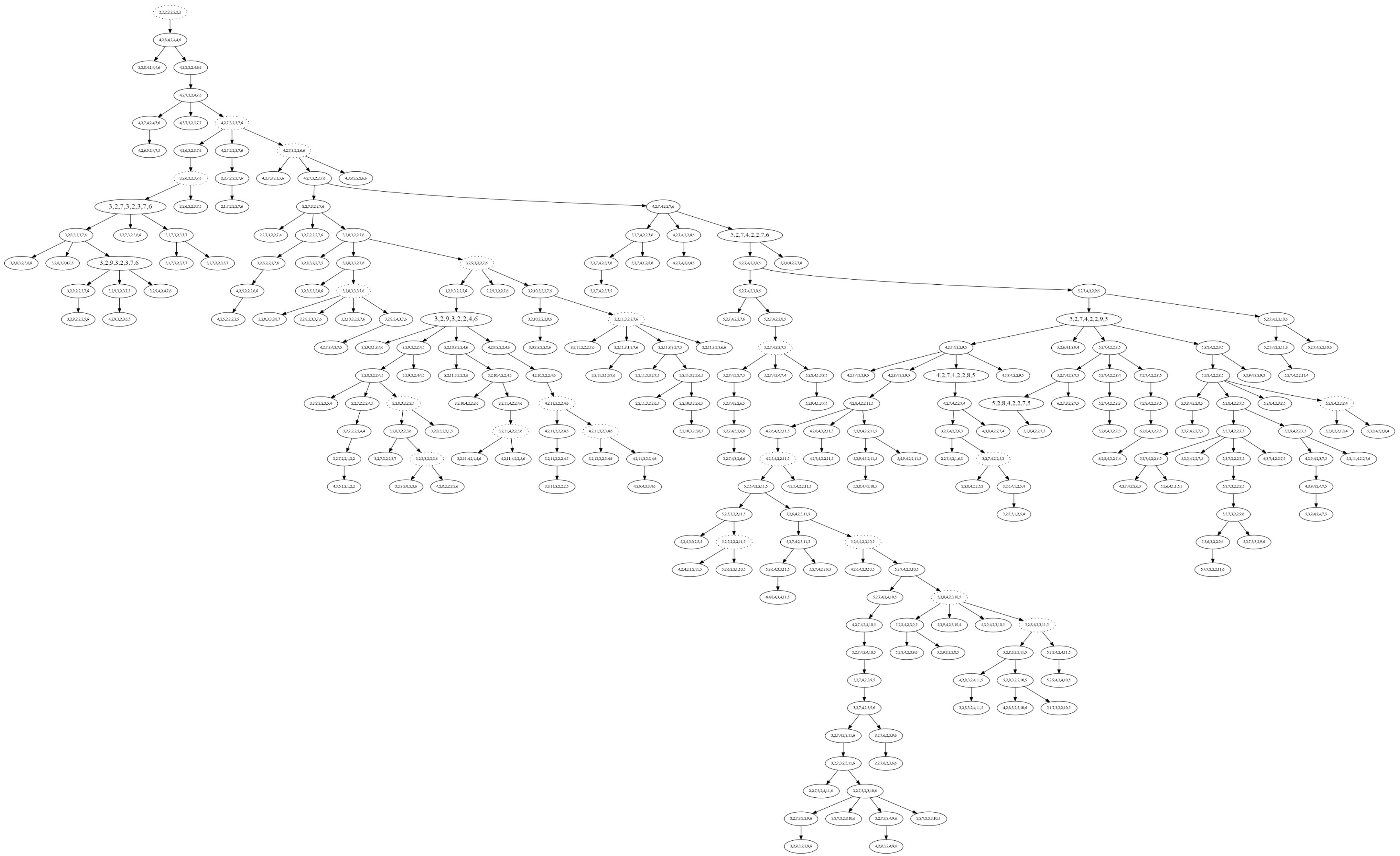

15L	Locus	250 nuclei													Instability Index: 86.8	Average ploidy: 3.0			
		15,2%	10,8%	10,8%	5,2%	4,4%	4,0%	3,2%	3,2%	2,8%	2,8%	2,8%	2,4	2,0	30,4%	GENE	GAIN	LOSS	AvgSig
	1q															COX2	54%	5%	3,9
	8p															DBC2	1%	82%	2,2
	8q															MYC	100%	0%	7,8
	11q															CCND1	50%	8%	3,5
	16q															CDH1	1%	89%	2,1
	17p															TP53	9%	60%	2,5
	17q															HER2	96%	1%	7,3
	20q															ZNF217	96%	1%	5,4

N

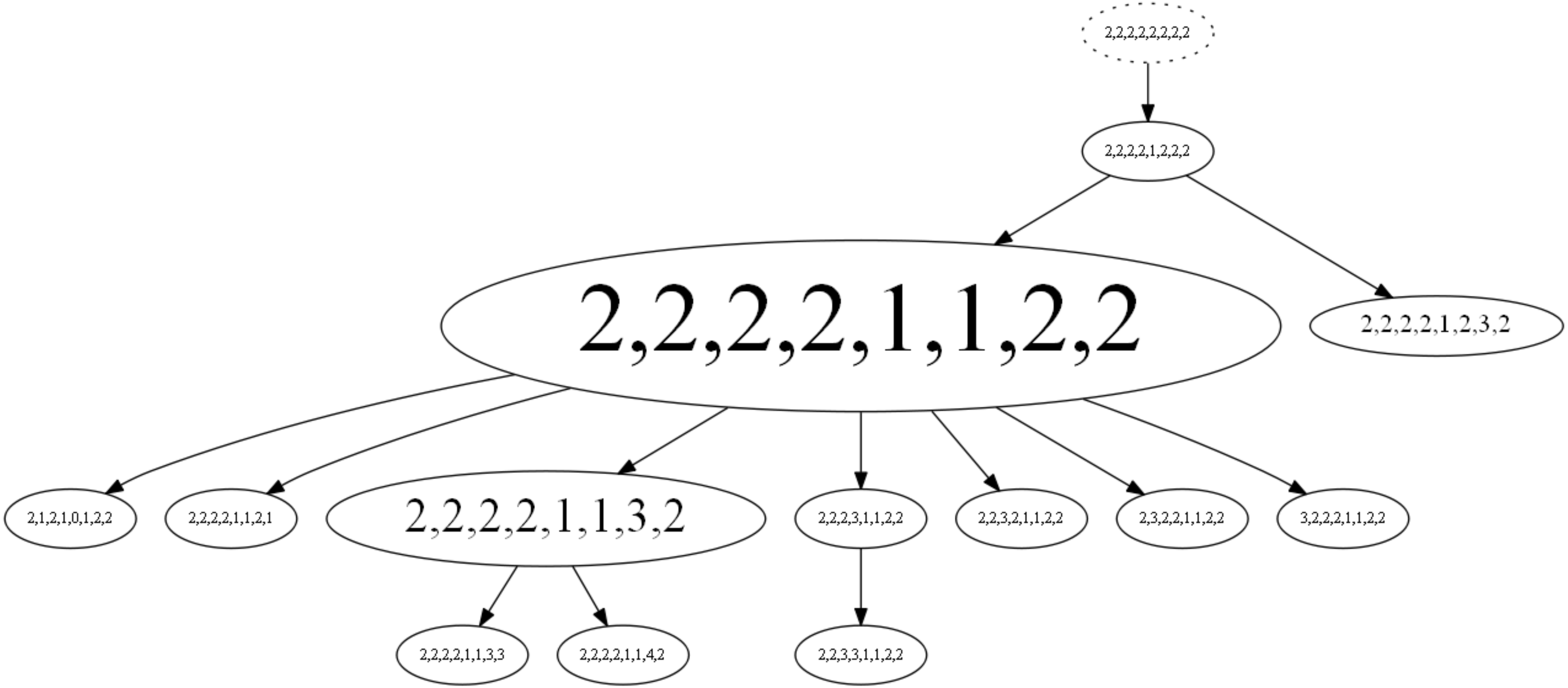

16L

Locus	250 nuclei		Instability Index: 5.2				Average ploidy: 2.0
	88,0%	5,6%	6,4%	GENE	GAIN	LOSS	AvgSig
1q				COX2	0%	0%	2,0
8p				DBC2	1%	0%	2,0
8q				MYC	1%	0%	2,0
11q				CCND1	1%	0%	2,0
16q				CDH1	0%	100%	1,0
17p				TP53	0%	98%	1,0
17q				HER2	7%	0%	2,1
20q				ZNF217	0%	1%	2,0

O

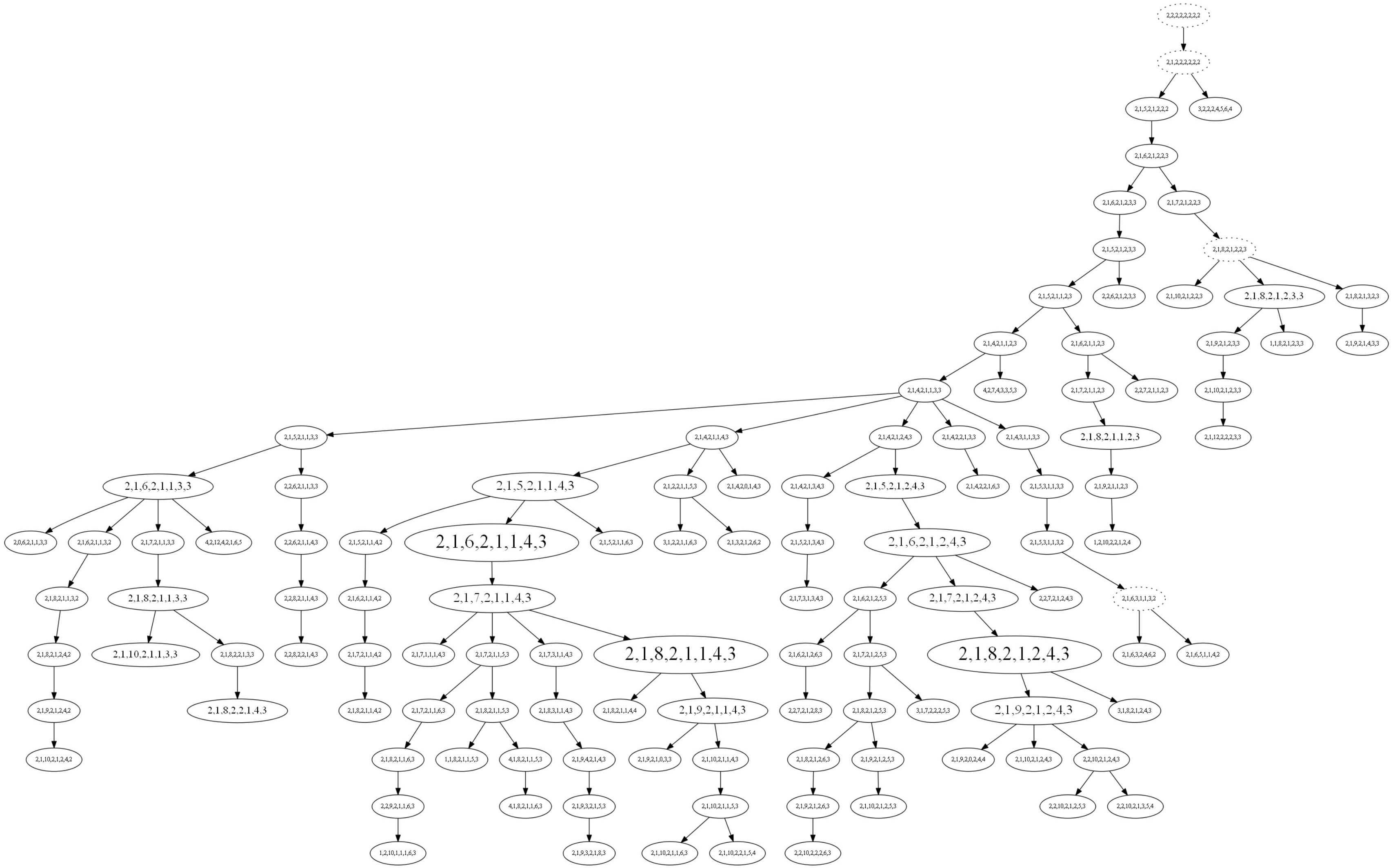

17L	Locus	250 nuclei									Instability Index: 45.2				Average ploidy: 2.1
		38,4%	21,6%	6,4%	5,2%	3,6%	3,2%	2,4	2,0	2,0	15,2%	GENE	GAIN	LOSS	AvgSig
	1q											COX2	2%	8%	2,0
	8p											DBC2	0%	93%	1,1
	8q											MYC	99%	0%	7,2
	11q											CCND1	4%	7%	2,1
	16q											CDH1	0%	92%	1,1
	17p											TP53	3%	67%	1,4
	17q											HER2	92%	0%	3,9
	20q											ZNF217	86%	0%	3,0

P

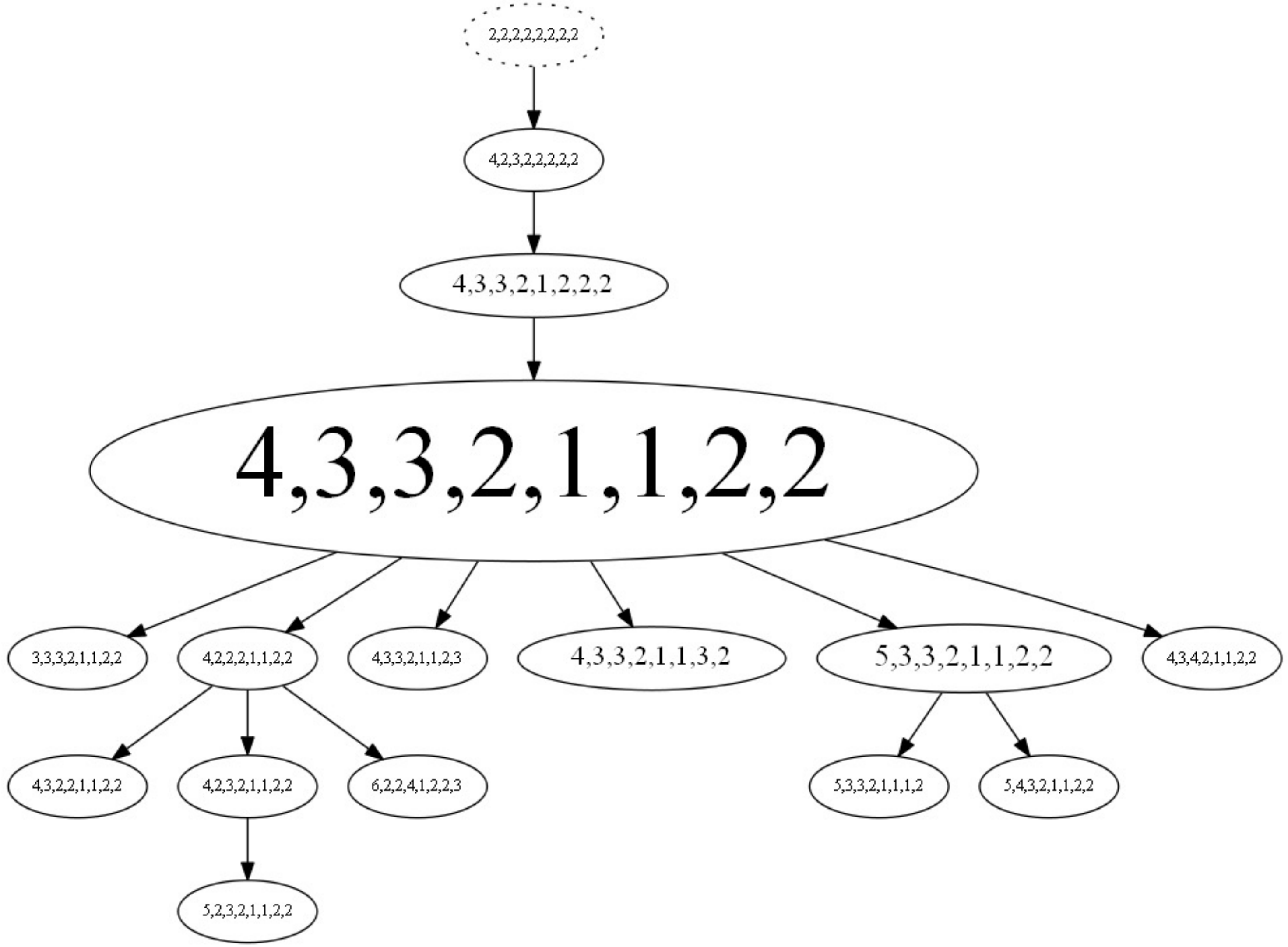

	Locus	250 nuclei	Instability Index: 6.0	Average ploidy: 2.0			
		94,0%	6,0%	GENE	GAIN	LOSS	AvgSig
18L	1q			COX2	100%	0%	4,0
	8p			DBC2	98%	0%	3,0
	8q			MYC	99%	0%	3,0
	11q			CCND1	0%	0%	2,0
	16q			CDH1	0%	100%	1,0
	17p			TP53	0%	98%	1,0
	17q			HER2	1%	0%	2,0
	20q			ZNF217	1%	0%	2,0

Q

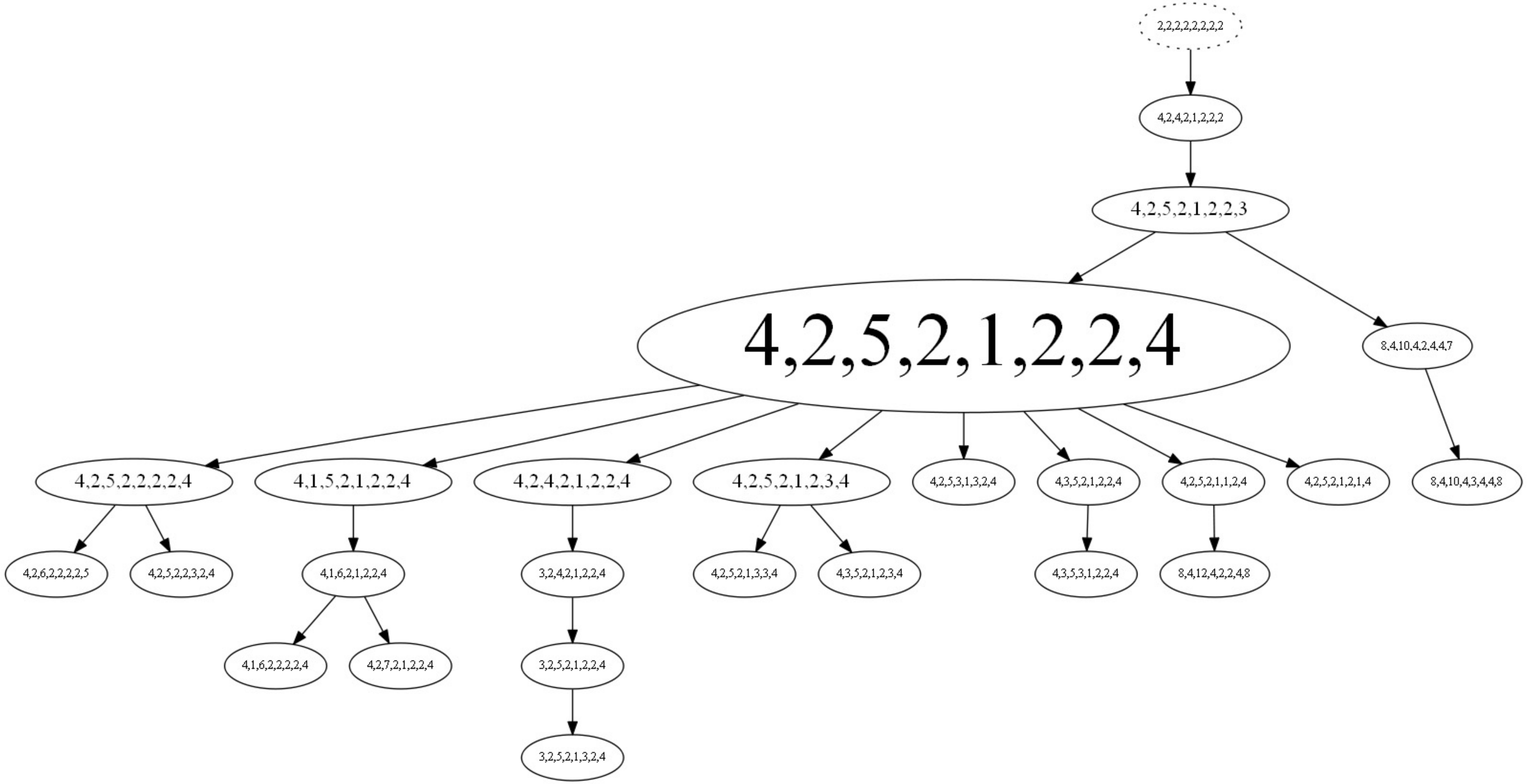

	Locus	250 nuclei		Instability Index: 10.0				Average ploidy: 2.0
		89,2%	2,8%	8,0%	GENE	GAIN	LOSS	AvgSig
19L	1q				COX2	100%	0%	4,0
	8p				DBC2	1%	3%	2,0
	8q				MYC	100%	0%	5,1
	11q				CCND1	1%	0%	2,0
	16q				CDH1	0%	98%	1,0
	17p				TP53	2%	1%	2,0
	17q				HER2	2%	0%	2,0
	20q				ZNF217	100%	0%	4,0

R

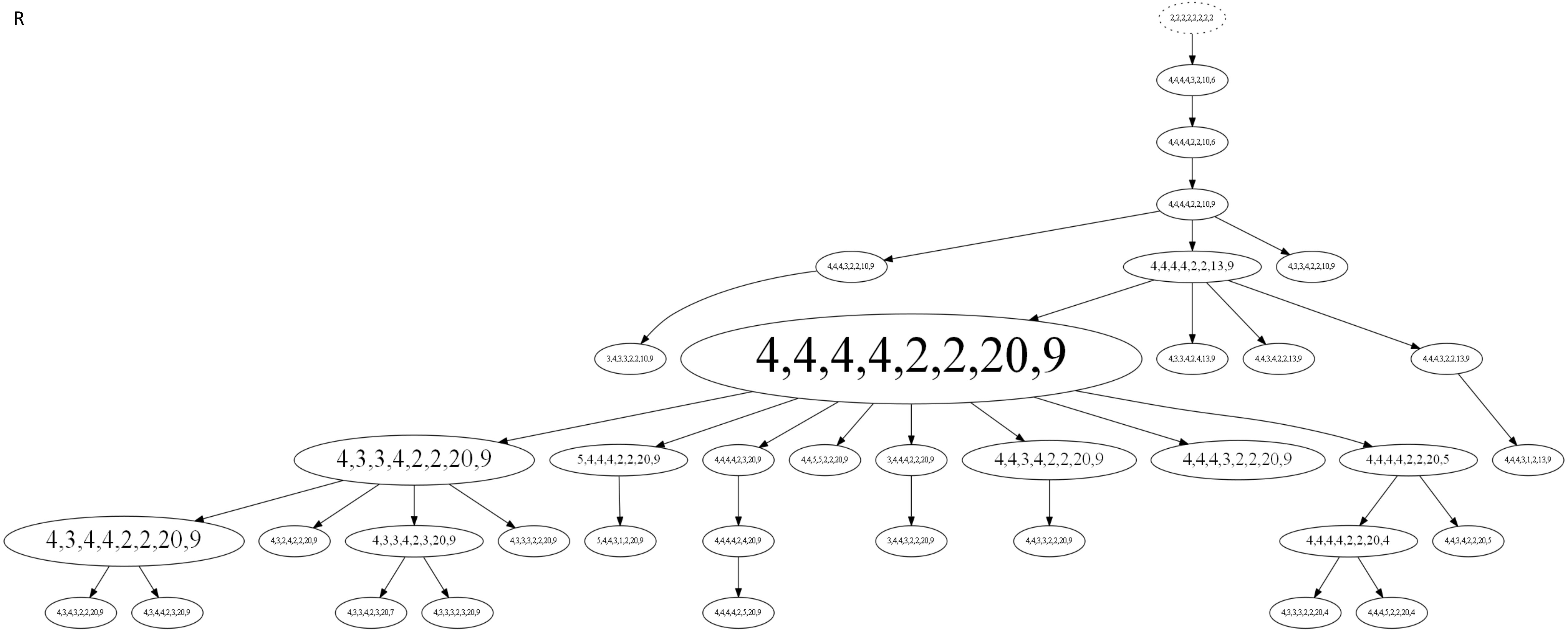

20L	Locus	250 nuclei				Instability Index: 14.8					Average ploidy: 4.0
		61,2%	12,0%	6,8%	5,6%	5,6%	8,8%	GENE	GAIN	LOSS	AvgSig
	1q							COX2	2%	1%	4,0
	8p							DBC2	0%	21%	3,8
	8q							MYC	0%	20%	3,8
	11q							CCND1	1%	9%	3,9
	16q							CDH1	0%	100%	2,0
	17p							TP53	0%	99%	2,1
	17q							HER2	100%	0%	19,5
	20q							ZNF217	98%	0%	8,8

S

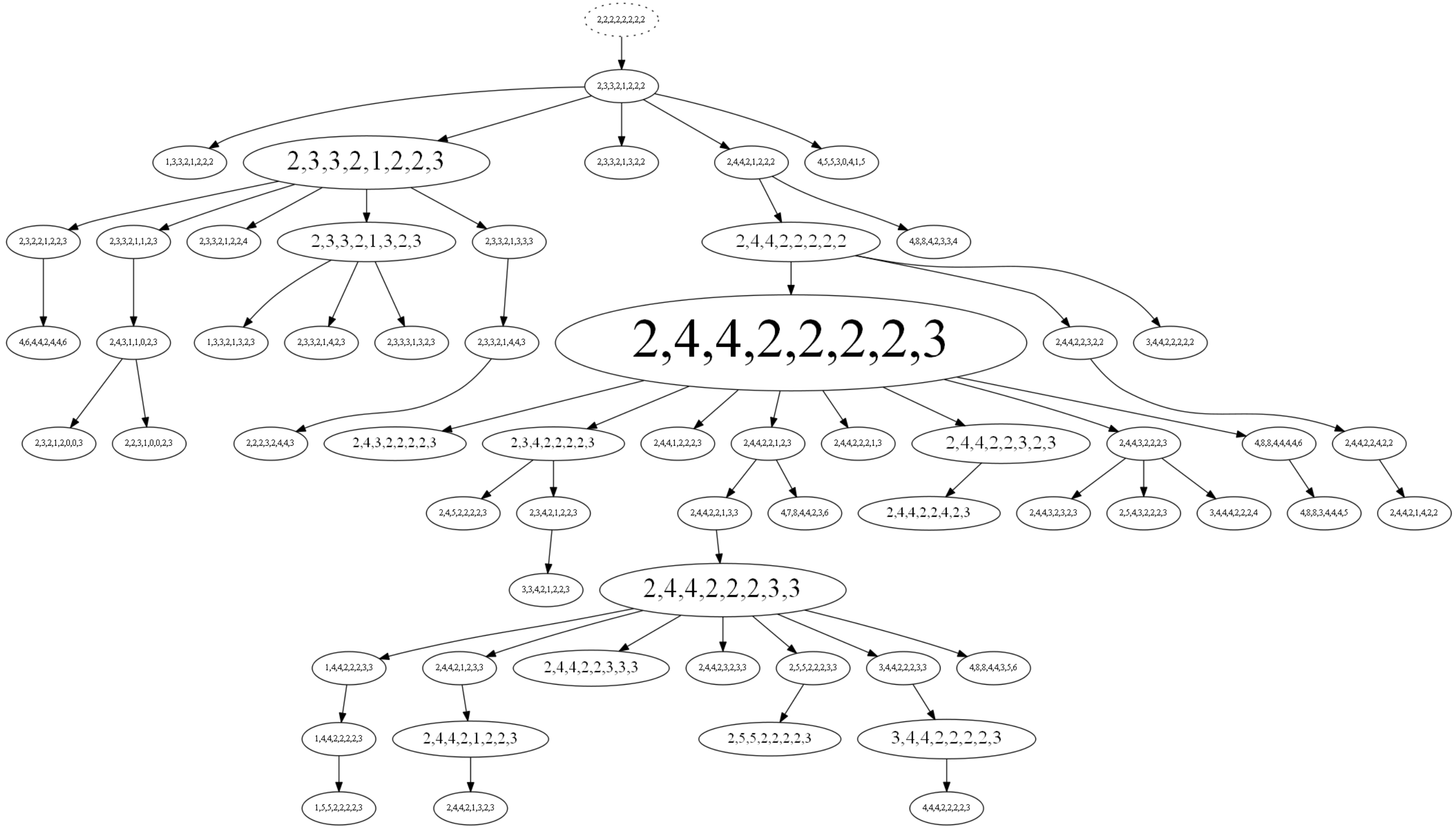

21L

Locus	250 nuclei								Instability Index: 24.0				Average ploidy: 2.1
	44,0%	10,4%	6,0%	5,2%	4,8%	4,8%	3,2%	2,8%	18,8%	GENE	GAIN	LOSS	AvgSig
1q										COX2	6%	2%	2,1
8p										DBC2	99%	0%	3,9
8q										MYC	98%	0%	3,9
11q										CCND1	3%	3%	2,1
16q										CDH1	0%	24%	1,8
17p										TP53	17%	4%	2,2
17q										HER2	13%	2%	2,2
20q										ZNF217	92%	0%	3,0

Supplemental Figure S1
