# Supplementary material for: Single Cell Genetic Profiling of Tumors of Breast Cancer Patients Aged 50 Years and Older Reveals Enormous Intratumor Heterogeneity Independent of Individual Prognosis
Source: Cancers (Basel). 2021 Jul 5;13(13):3366. doi: 10.3390/cancers13133366 (PMC8267950; doi:10.3390/cancers13133366)
Supplement: Supplementary file 1 [file cancers-13-03366-s001.zip › cancers-1245840-SI/Supplementary_Files/Supplemental Figures/Supplemental Figure S2.pdf]

A

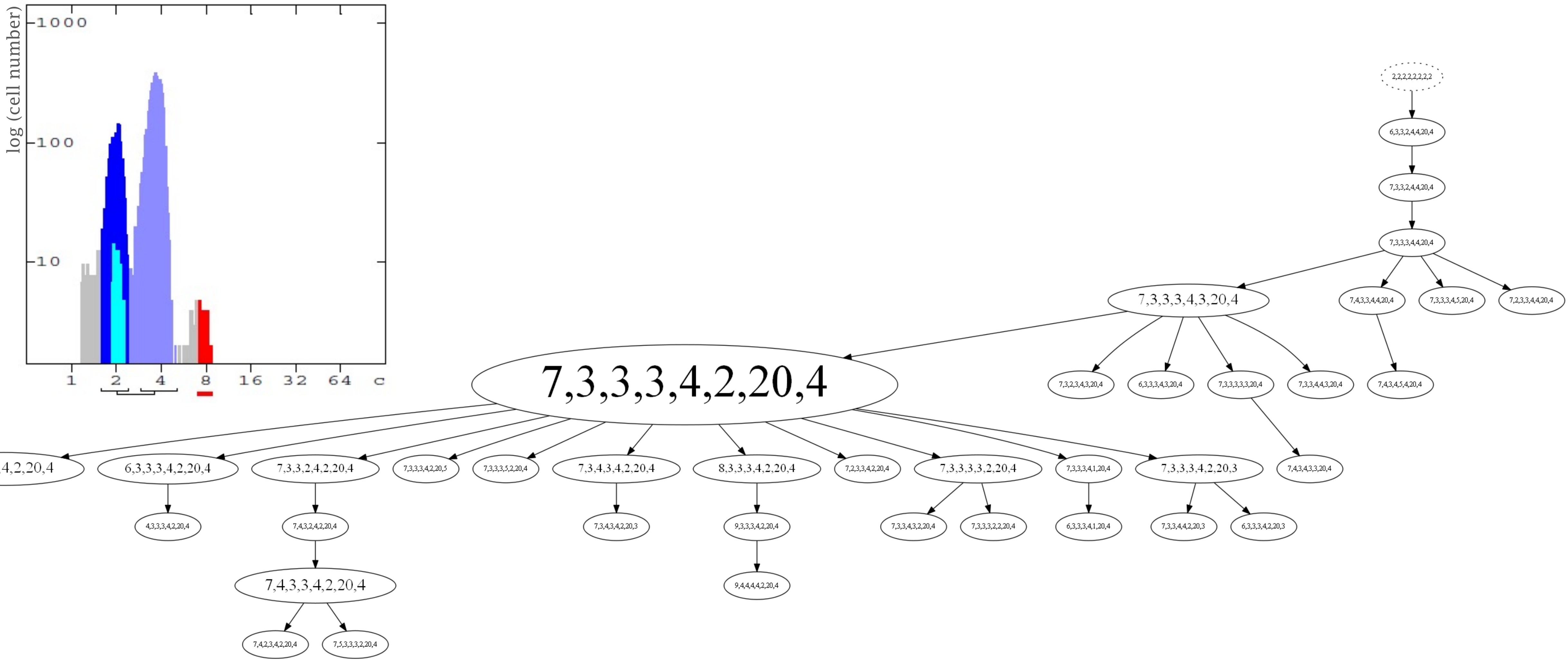

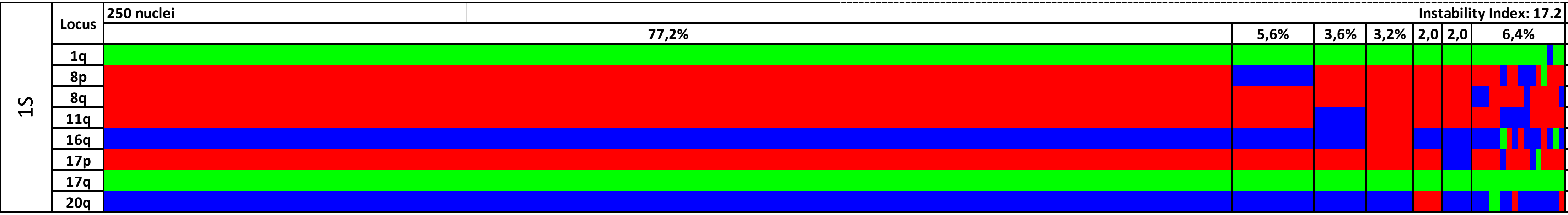

Average ploidy: 4.0

Locus	GENE	GAIN	LOSS	AvgSig
1q	COX2	100%	0%	7,0
8p	DBC2	0%	92%	3,1
8q	MYC	0%	98%	3,0
11q	CCND1	0%	94%	3,0
16q	CDH1	1%	4%	4,0
17p	TP53	0%	97%	2,1
17q	HER2	100%	0%	29,7
20q	ZNF217	1%	3%	4,0

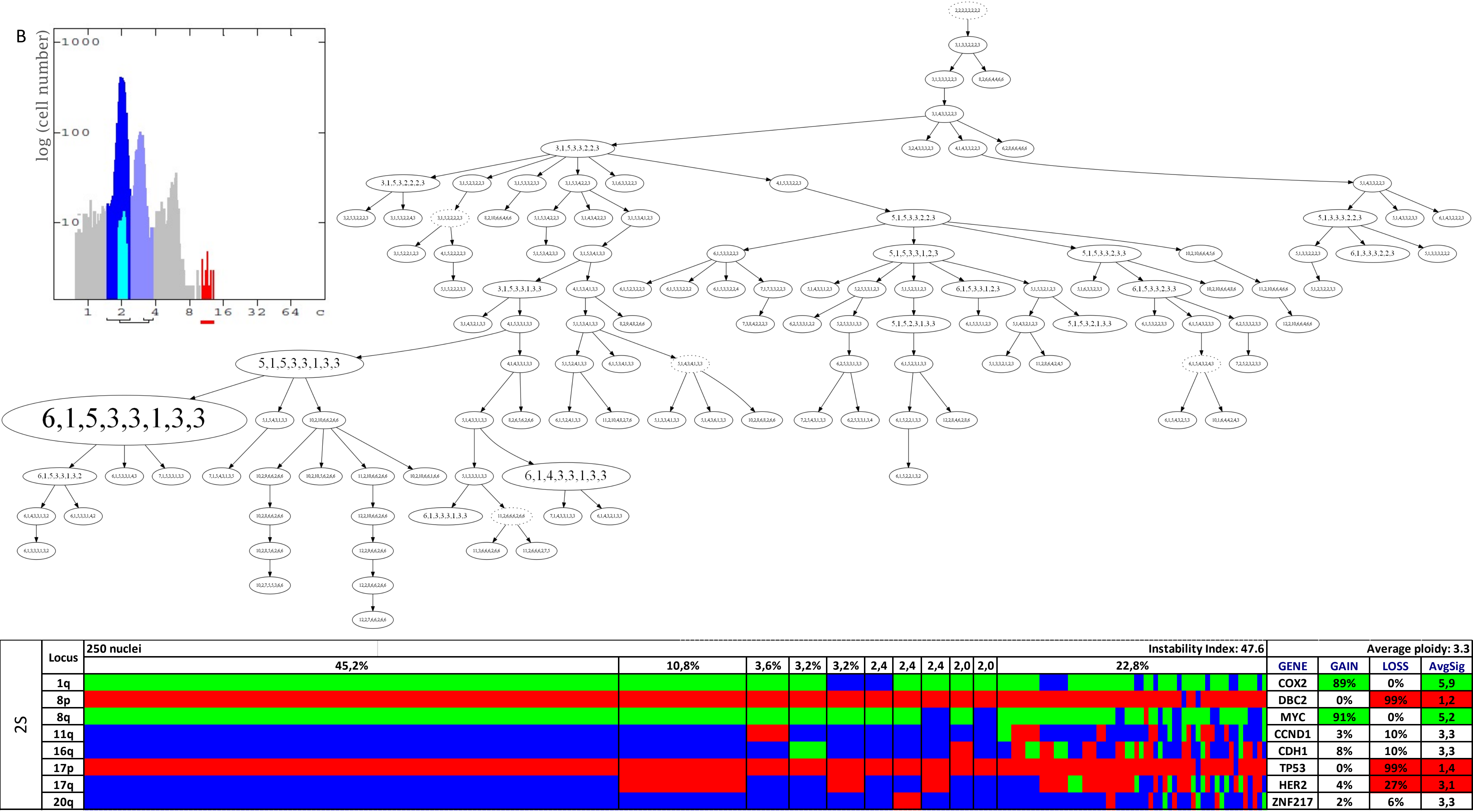

B
log (cell number)
1000
100
10
1
2
4
8
16
32
64
c
2,2,2,2,2,2,2,2
3,1,3,3,2,2,2,3
3,1,5,3,3,2,2,3
5,1,5,3,3,2,2,3
5,1,3,3,3,2,2,3
3,1,5,3,2,2,2,3
5,1,5,3,3,1,2,3
5,1,5,3,3,2,3,3
6,1,3,3,3,2,2,3
3,1,5,3,3,1,3,3
6,1,5,3,3,1,2,3
6,1,5,3,3,2,3,3
5,1,5,2,3,1,3,3
5,1,5,3,2,1,3,3
5,1,5,3,3,1,3,3
6,1,5,3,3,1,3,3
6,1,5,3,3,1,3,2
6,1,4,3,3,1,3,3
6,1,3,3,3,1,3,3
2S
Locus
250 nuclei
Instability Index: 47.6
Average ploidy: 3.3
45,2%
10,8%
3,6%
3,2%
3,2%
2,4
2,4
2,4
2,0
2,0
22,8%
1q
8p
8q
11q
16q
17p
17q
20q
GENE | GAIN | LOSS | AvgSig
COX2 | 89% | 0% | 5,9
DBC2 | 0% | 99% | 1,2
MYC | 91% | 0% | 5,2
CCND1 | 3% | 10% | 3,3
CDH1 | 8% | 10% | 3,3
TP53 | 0% | 99% | 1,4
HER2 | 4% | 27% | 3,1
ZNF217 | 2% | 6% | 3,3

C

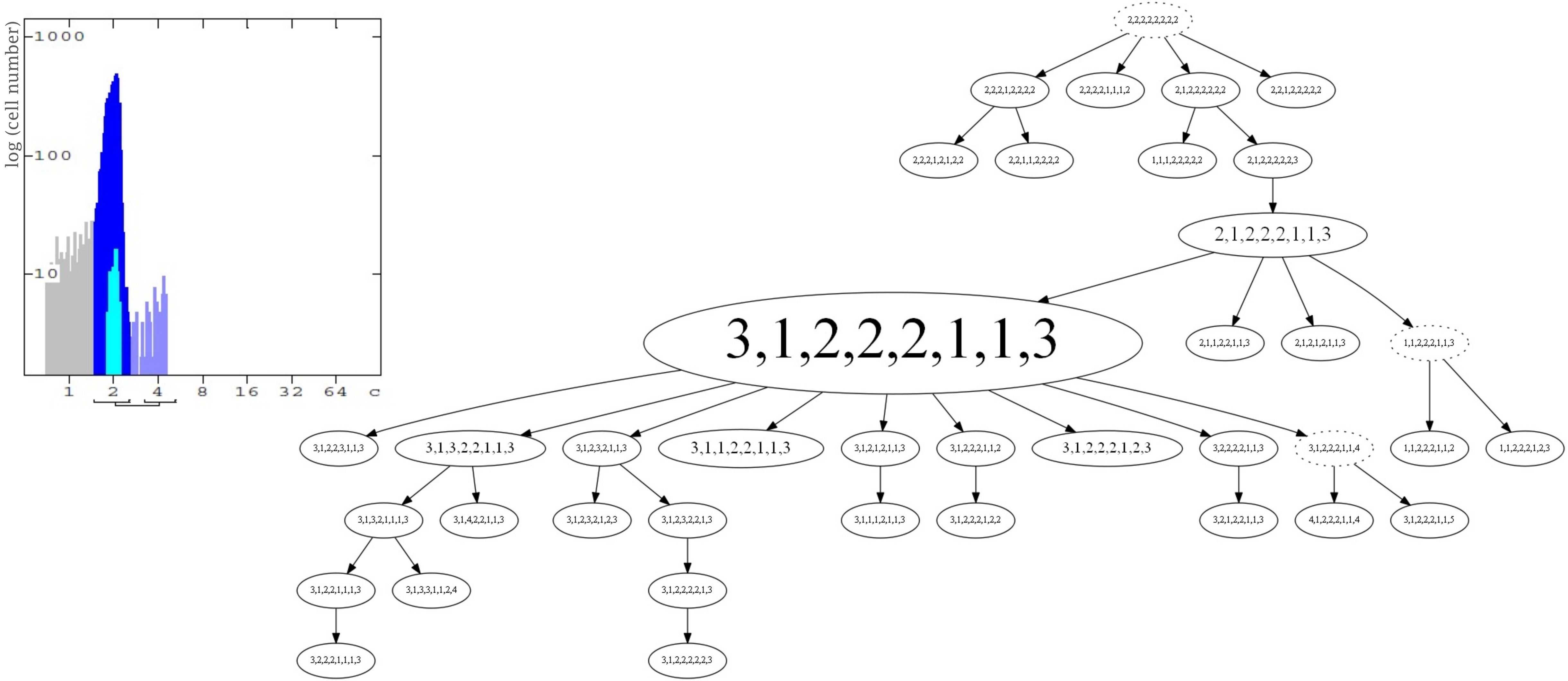

3S	Locus	250 nuclei				Instability Index: 14.4	Average ploidy: 2.0			
		76,0%	4,4%	2,4	2,0	15,2%	GENE	GAIN	LOSS	AvgSig
	1q						COX2	90%	1%	2,9
	8p						DBC2	0%	97%	1,0
	8q						MYC	2%	5%	2,0
	11q						CCND1	2%	3%	2,0
	16q						CDH1	1%	2%	2,0
	17p						TP53	0%	96%	1,0
	17q						HER2	0%	93%	1,1
	20q						ZNF217	96%	0%	3,0

D

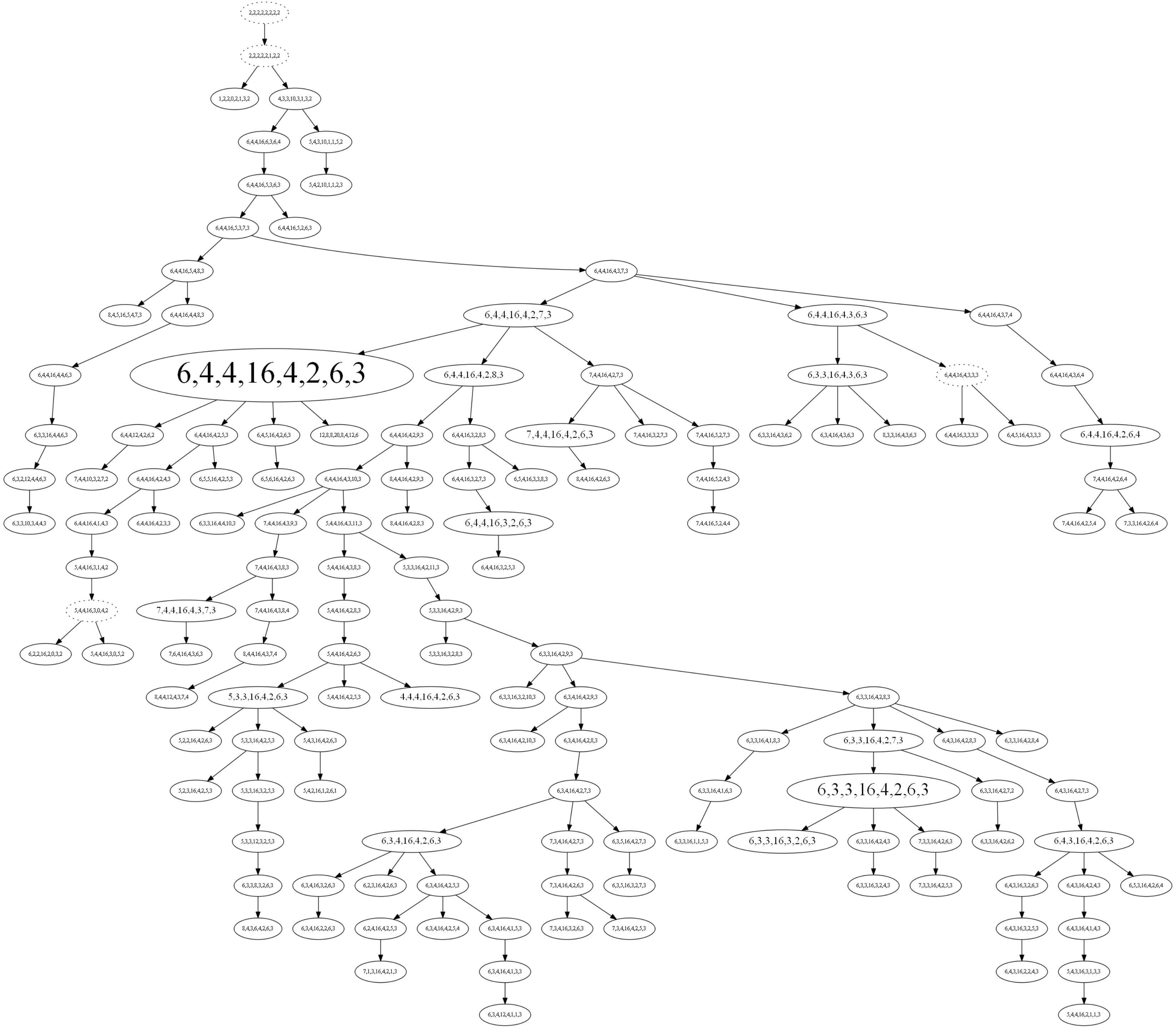
6,4,4,16,4,2,7,3
6,4,4,16,4,3,6,3
6,4,4,16,4,2,6,3
6,4,4,16,4,2,8,3
6,3,3,16,4,3,6,3
7,4,4,16,4,2,6,3
6,4,4,16,4,2,6,4
6,4,4,16,3,2,6,3
7,4,4,16,4,3,7,3
5,3,3,16,4,2,6,3
4,4,4,16,4,2,6,3
6,3,3,16,4,2,7,3
6,3,3,16,4,2,6,3
6,3,4,16,4,2,6,3
6,3,3,16,3,2,6,3
6,4,3,16,4,2,6,3

E

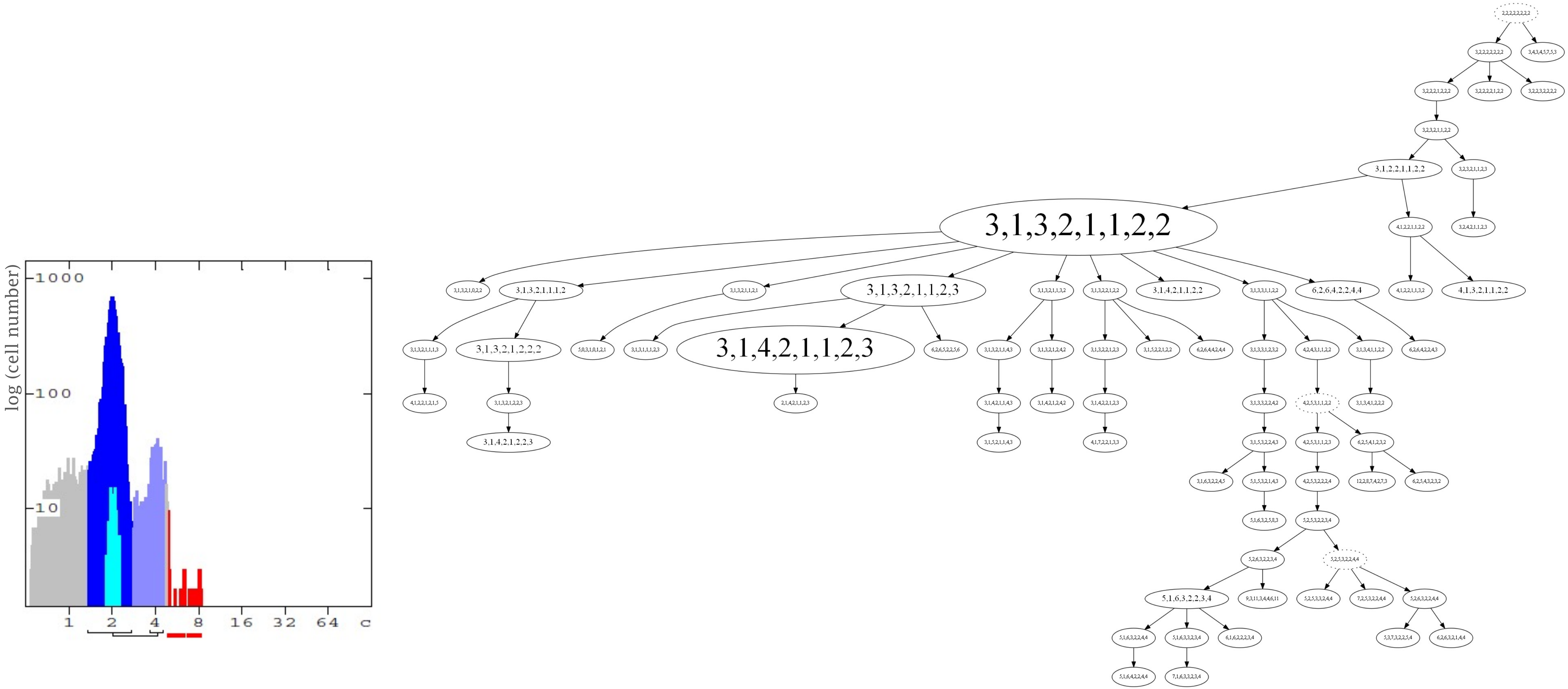

5S	Locus	250 nuclei						Instability Index: 28.4	Average ploidy: 2.3			
		36,0%	29,6%	3,6%	3,2%	2,4	2,0	23,2%	GENE	GAIN	LOSS	AvgSig
	1q								COX2	98%	0%	3,4
	8p								DBC2	0%	96%	1,1
	8q								MYC	95%	0%	3,6
	11q								CCND1	5%	8%	2,2
	16q								CDH1	0%	95%	1,2
	17p								TP53	1%	89%	1,2
	17q								HER2	7%	7%	2,3
	20q								ZNF217	39%	4%	2,6

F

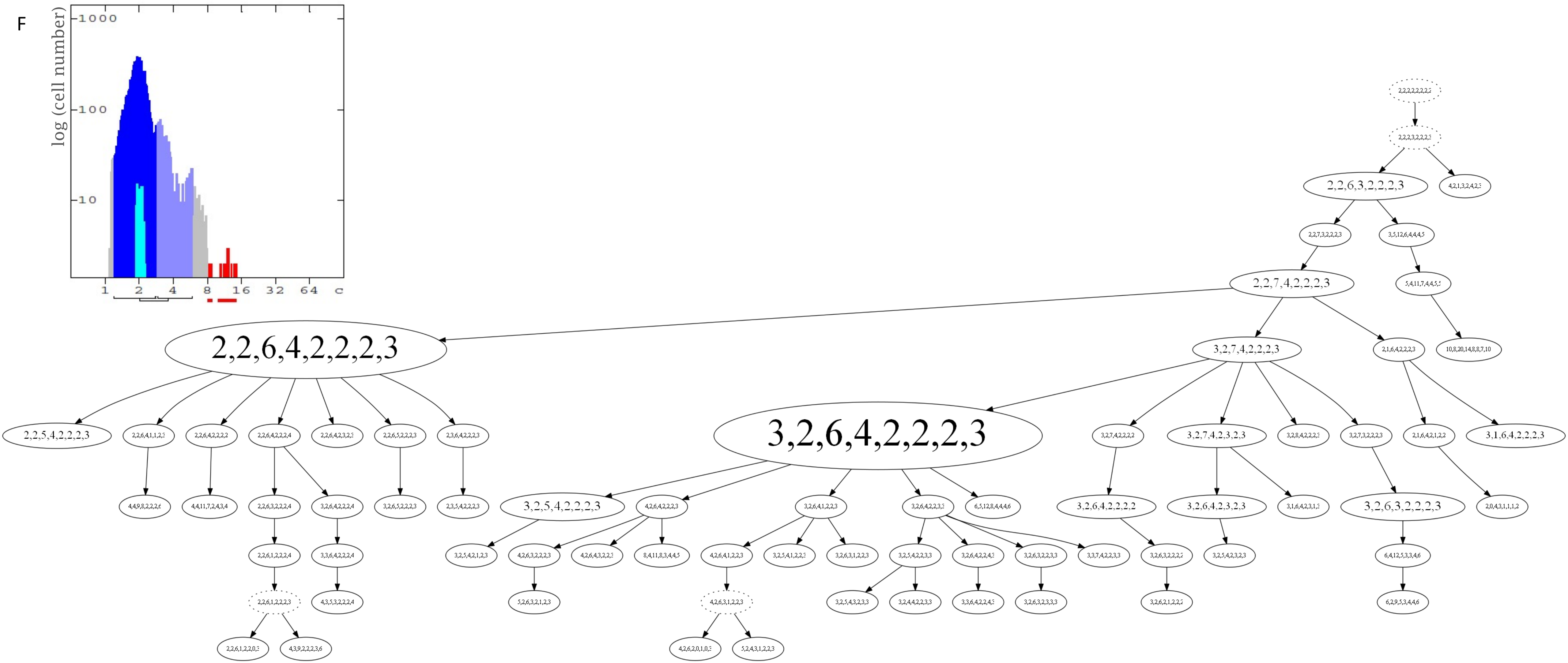

S9	Locus	250 nuclei						Instability Index: 27.2	Average ploidy: 2.1			
		48,4%	27,2%	2,8%	2,8%	2,4	2,0	14,4%	GENE	GAIN	LOSS	AvgSig
	1q								COX2	67%	0%	2,8
	8p								DBC2	4%	3%	2,1
	8q								MYC	100%	0%	6,2
	11q								CCND1	98%	1%	4,0
	16q								CDH1	1%	6%	2,0
	17p								TP53	4%	3%	2,1
	17q								HER2	5%	3%	2,1
	20q								ZNF217	96%	0%	3,1

G

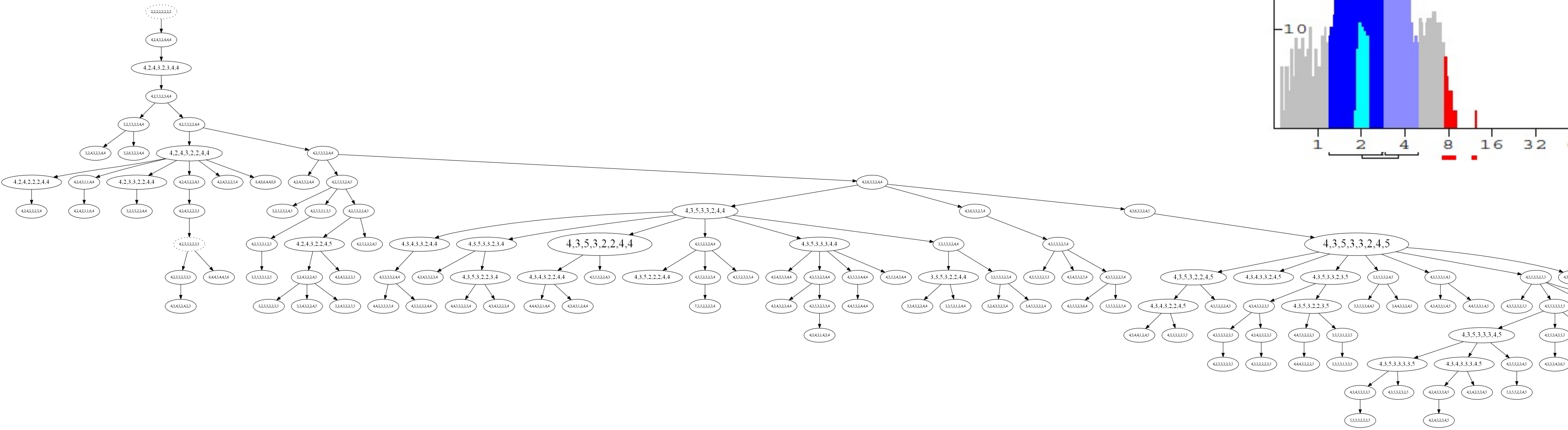

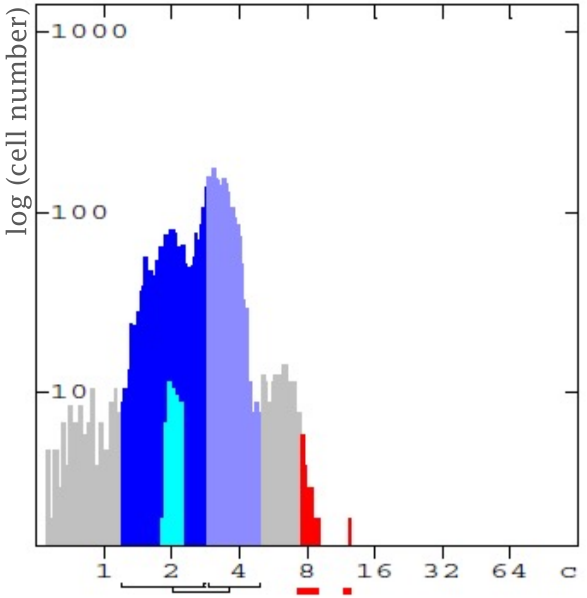

7S	Locus	250 nuclei									Instability Index: 51.2	Average ploidy: 3.0			
		18,4%	16,8%	7,2%	6,8%	4,0%	3,6%	2,4	2,4	2,4	2,0	34,0%	GENE	GAIN	LOSS	AvgSig
	1q												COX2	88%	0%	3,9
	8p												DBC2	5%	21%	2,8
	8q												MYC	94%	0%	4,6
	11q												CCND1	2%	9%	2,9
	16q												CDH1	2%	53%	2,5
	17p												TP53	4%	76%	2,2
	17q												HER2	80%	3%	3,9
	20q												ZNF217	97%	0%	4,5

H

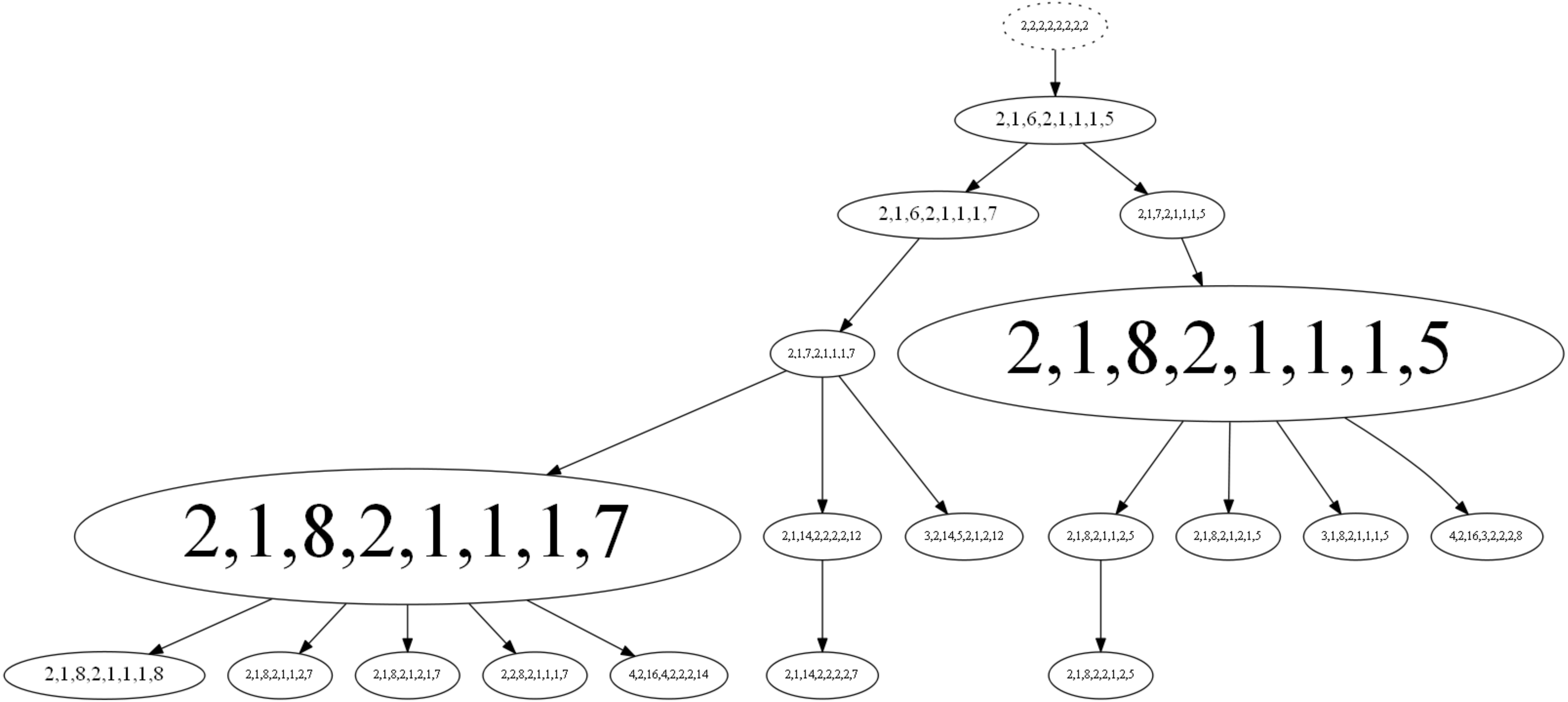
2,2,2,2,2,2,2,2
2,1,6,2,1,1,1,5
2,1,6,2,1,1,1,7
2,1,7,2,1,1,1,5
2,1,7,2,1,1,1,7
2,1,8,2,1,1,1,5
2,1,8,2,1,1,1,7
2,1,14,2,2,2,2,12
3,2,14,5,2,1,2,12
2,1,8,2,1,1,2,5
2,1,8,2,1,2,1,5
3,1,8,2,1,1,1,5
4,2,16,3,2,2,2,8
2,1,8,2,1,1,1,8
2,1,8,2,1,1,2,7
2,1,8,2,1,2,1,7
2,2,8,2,1,1,1,7
4,2,16,4,2,2,2,14
2,1,14,2,2,2,2,7
2,1,8,2,2,1,2,5

I

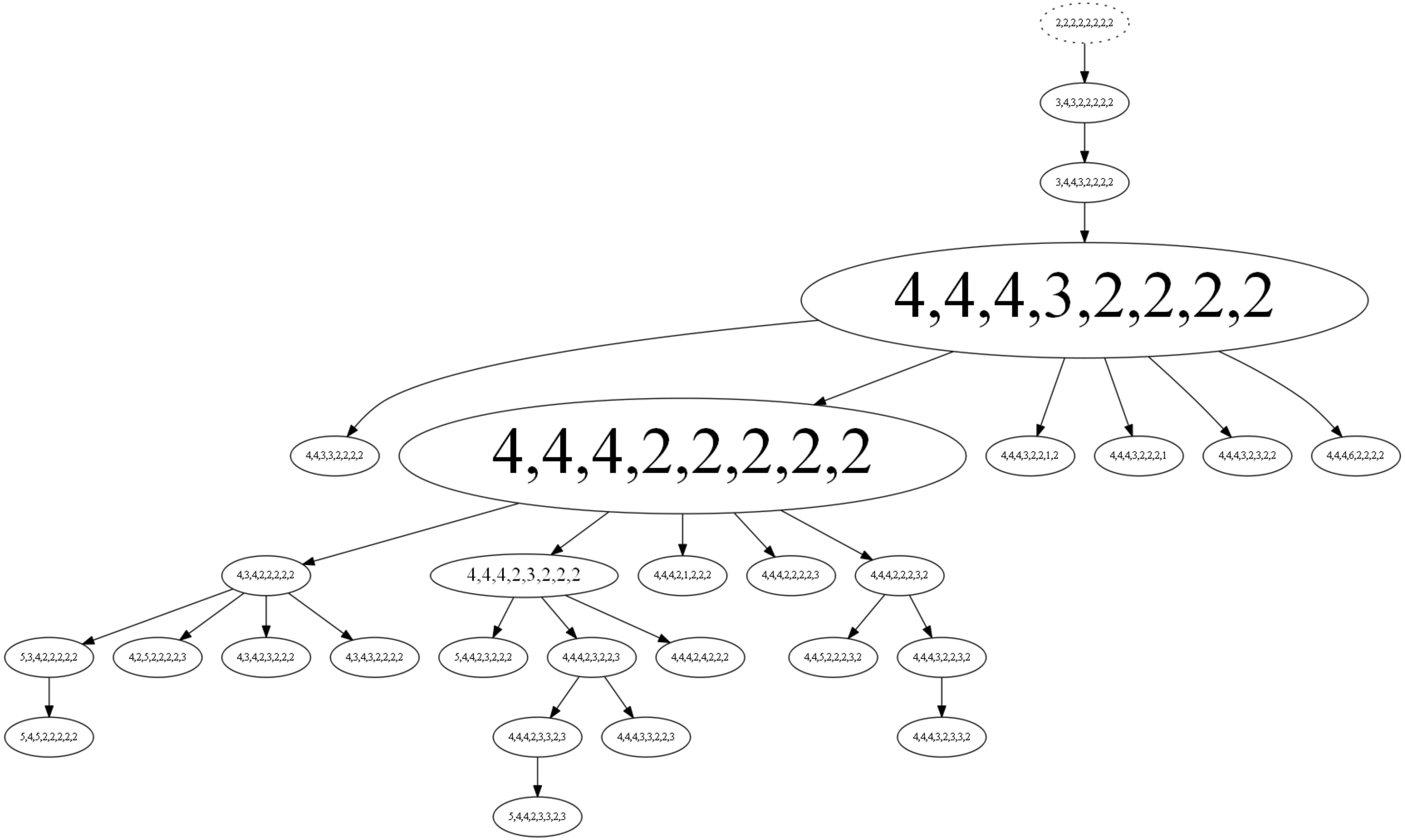

S6	Locus	250 nuclei			Instability Index: 11.2	Average ploidy: 3.0			
		58,0%	29,6%	2,8%	9,6%	GENE	GAIN	LOSS	AvgSig
	1q					COX2	99%	0%	4,0
	8p					DBC2	98%	0%	4,0
	8q					MYC	99%	0%	4,0
	11q					CCND1	0%	38%	2,6
	16q					CDH1	0%	94%	2,1
	17p					TP53	0%	98%	2,0
	17q					HER2	0%	98%	2,0
	20q					ZNF217	0%	97%	2,0

J

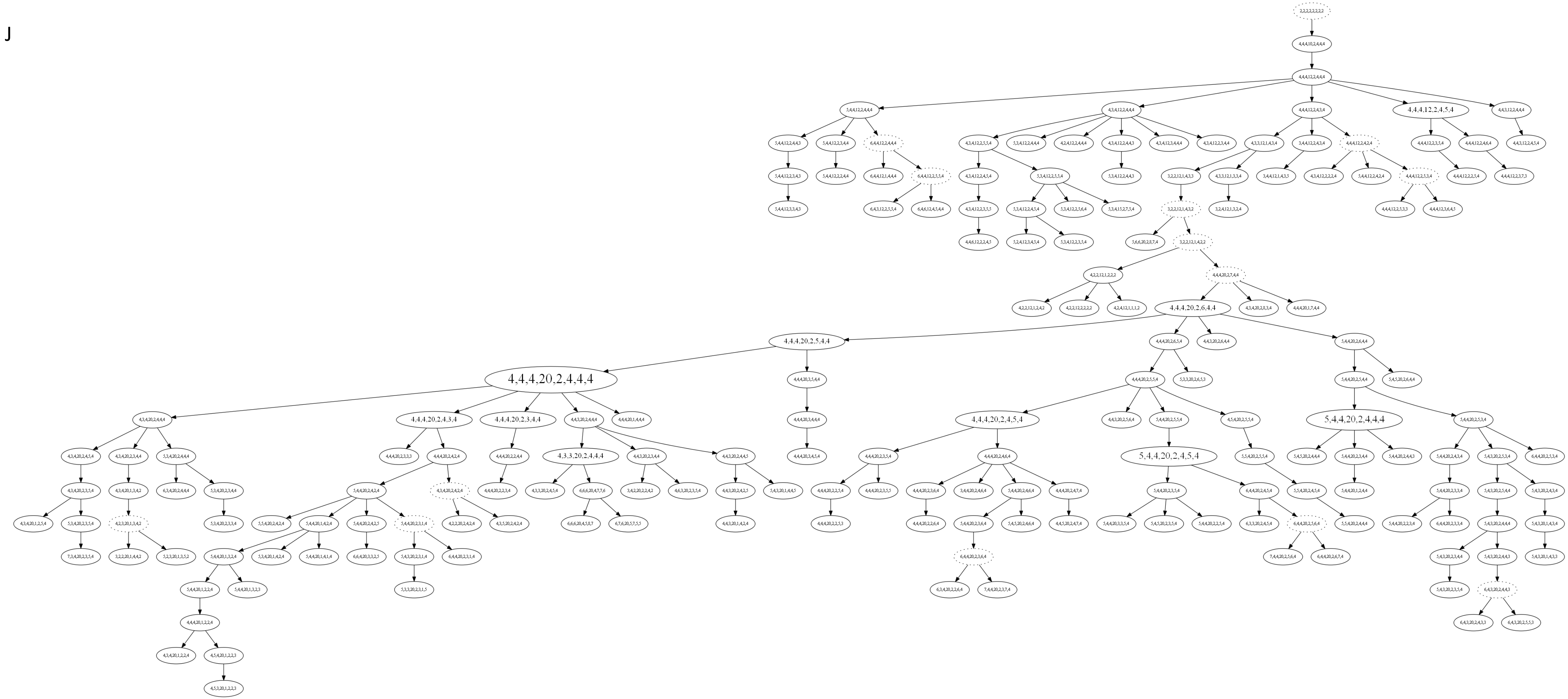

10S	Locus	250 nuclei													Instability Index: 68.8				Average ploidy: 4,0
		9,6%	6,8%	5,2%	4,8%	3,2%	3,2%	2,8%	2,8%	2,4	2,4	2,4	2,0	2,0	2,0	48,4%	GENE	GAIN	LOSS	AvgSig
	1q																COX2	49%	2%	4,5
	8p																DBC2	5%	21%	3,8
	8q																MYC	4%	16%	3,9
	11q																CCND1	100%	0%	18,4
	16q																CDH1	0%	99%	1,9
	17p																TP53	17%	29%	3,9
	17q																HER2	36%	23%	4,1
	20q																ZNF217	5%	10%	3,9

K

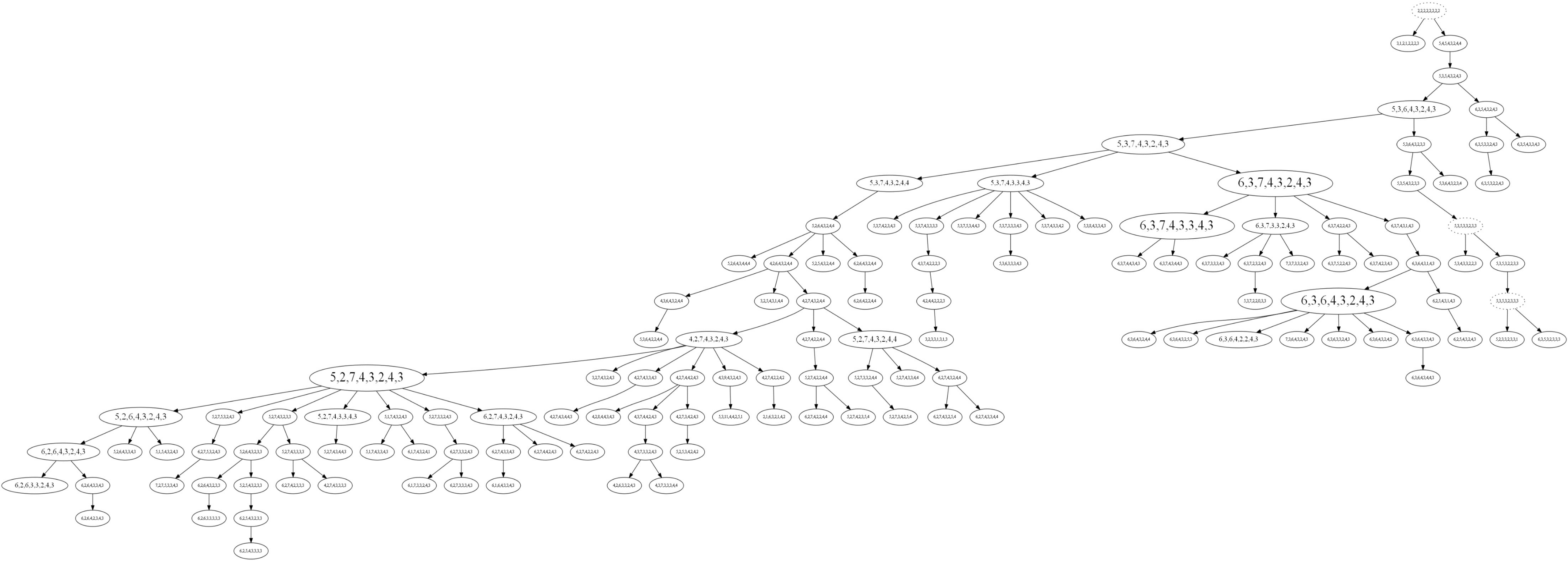

	Locus	250 nuclei										Instability Index: 50.0				Average ploidy: 3,0
11S		20,8%	17,2%	9,6%	6,4%	5,2%	4,0%	3,2%	3,2%	2,4	2,0	2,0	24,0%	GENE	GAIN	LOSS	AvgSig
	1q													COX2	98%	0%	5,4
	8p													DBC2	0%	50%	2,5
	8q													MYC	99%	0%	6,5
	11q													CCND1	84%	1%	3,8
	16q													CDH1	4%	11%	2,9
	17p													TP53	2%	72%	2,3
	17q													HER2	90%	2%	3,9
	20q													ZNF217	17%	3%	3,1

L

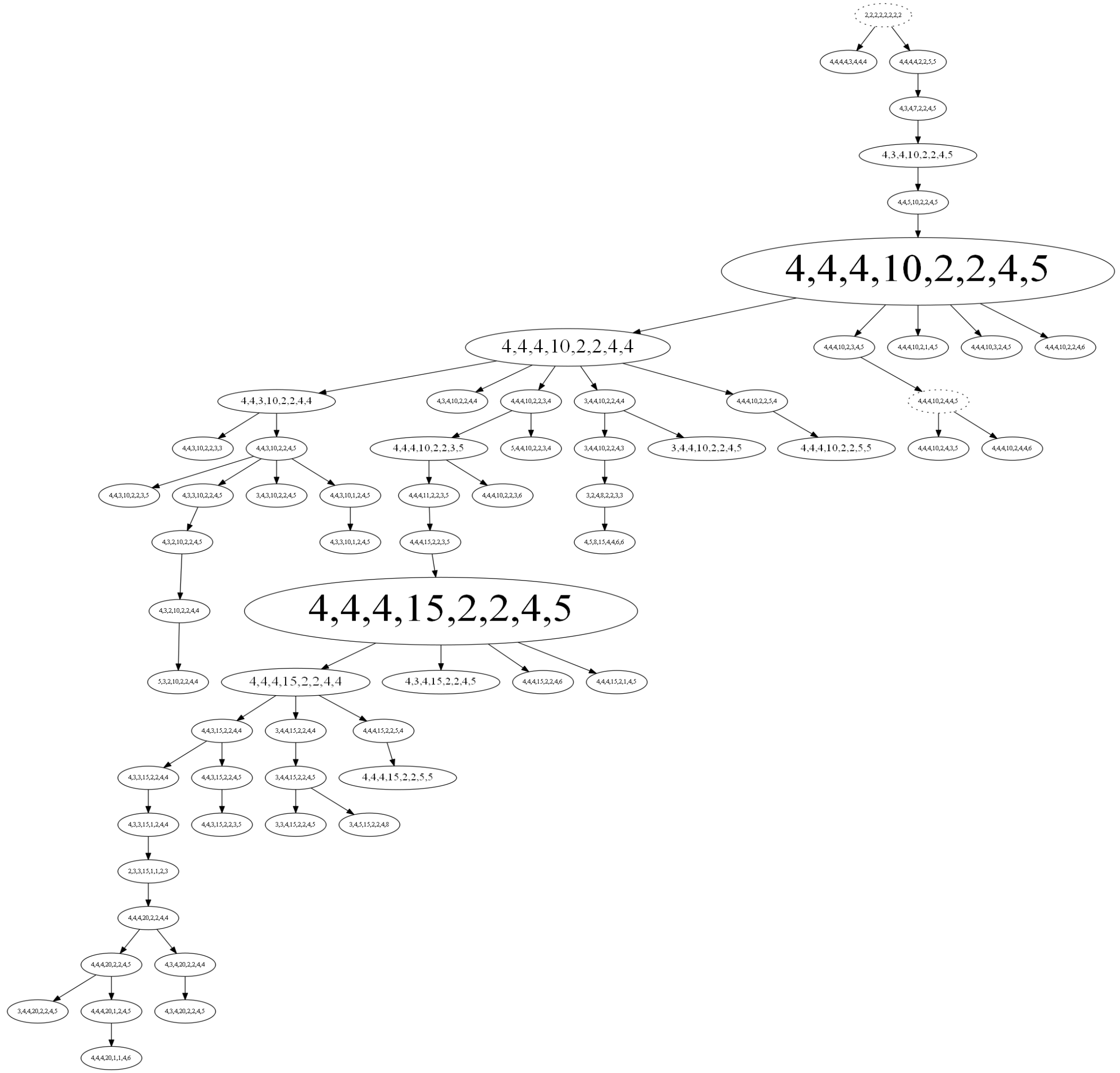

250 nuclei — Instability Index: 24.8 — Average ploidy: 4,0

Clone frequencies: 60,0% | 11,2% | 3,6% | 3,6% | 3,2% | 2,4 | 2,0 | 14,0%

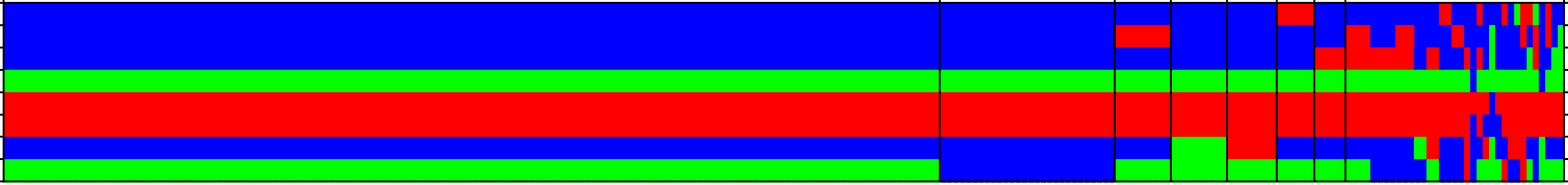

12S Locus	GENE	GAIN	LOSS	AvgSig
1q	COX2	1%	5%	3,9
8p	DBC2	1%	8%	3,9
8q	MYC	2%	8%	3,9
11q	CCND1	99%	0%	12,1
16q	CDH1	0%	100%	2,0
17p	TP53	0%	98%	2,0
17q	HER2	5%	6%	4,0
20q	ZNF217	81%	1%	4,8

M

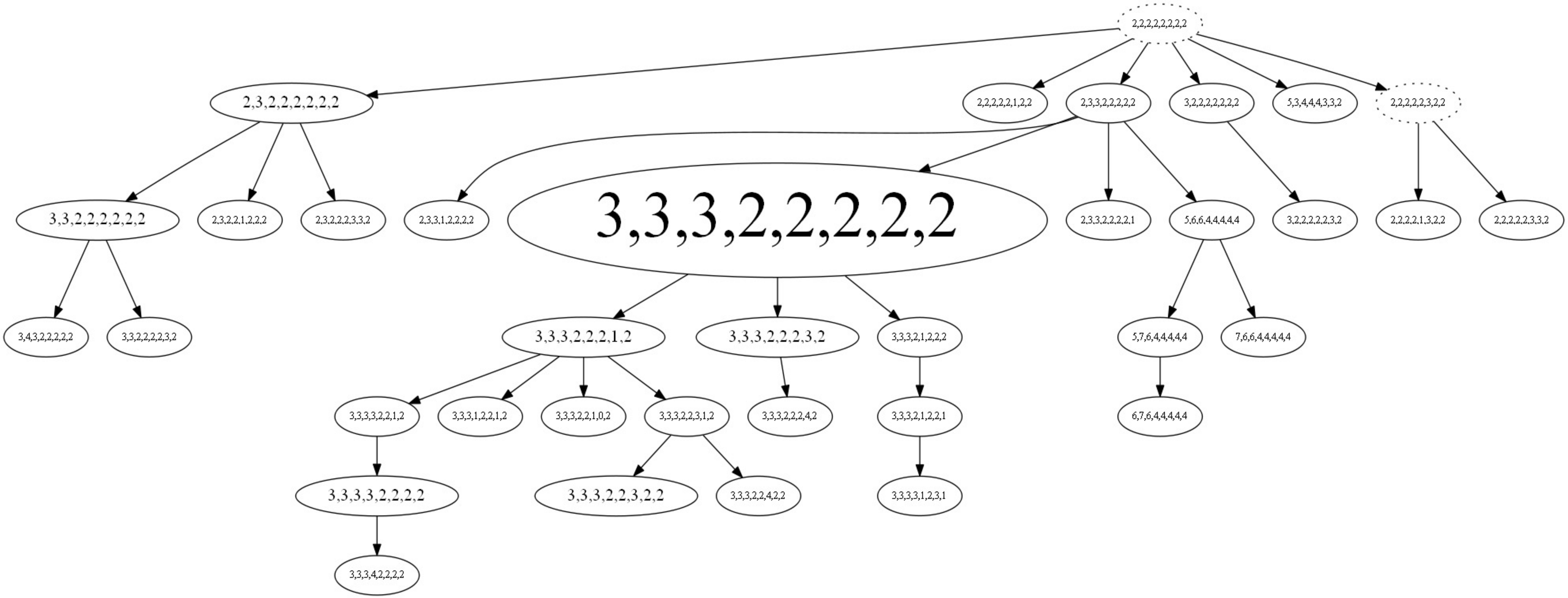

14S	Locus	250 nuclei				Instability Index: 13.6				Average ploidy: 2.0
		80,8%	2,8%	2,4	2,0	12,0%	GENE	GAIN	LOSS	AvgSig
	1q						COX2	94%	0%	3,0
	8p						DBC2	98%	0%	3,0
	8q						MYC	94%	0%	3,0
	11q						CCND1	4%	1%	2,1
	16q						CDH1	0%	2%	2,0
	17p						TP53	4%	1%	2,1
	17q						HER2	5%	3%	2,1
	20q						ZNF217	0%	1%	2,0

N

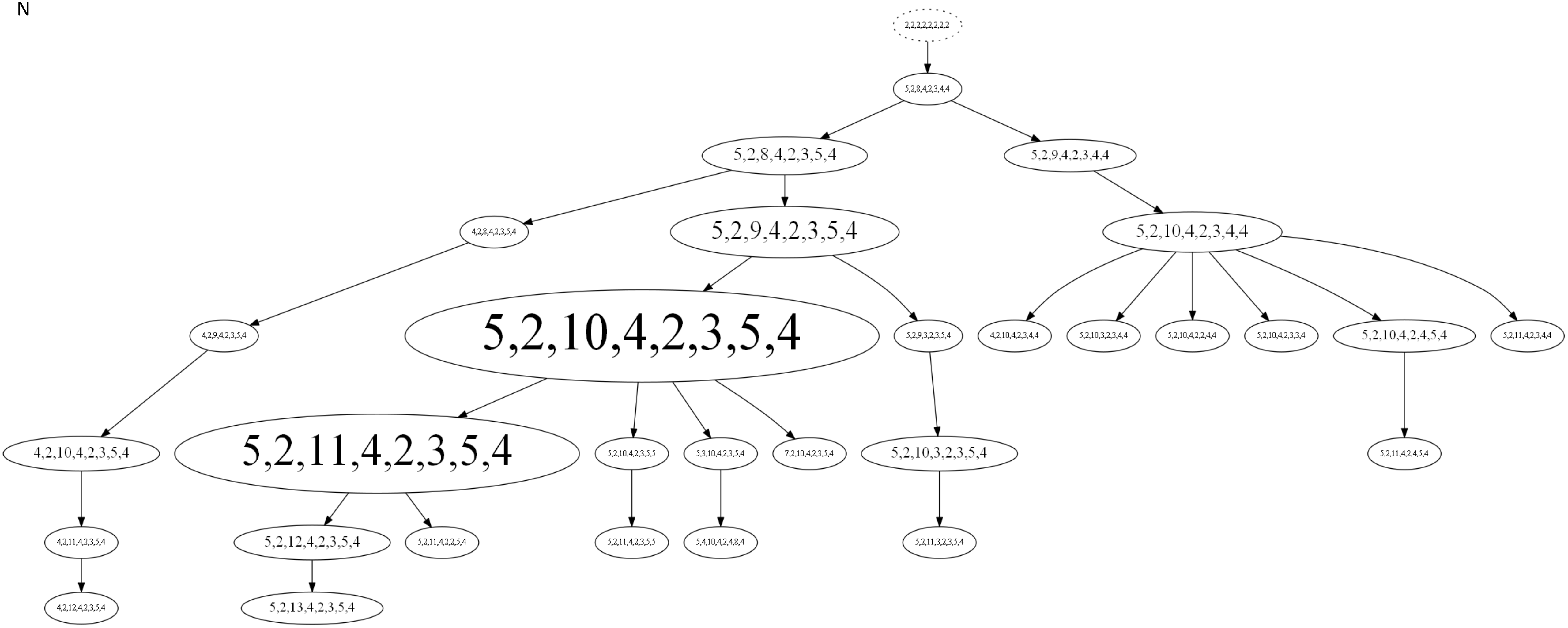

15S	Locus	250 nuclei			Instability Index: 12.0			Average ploidy: 4.0			
		79,6%	7,6%	4,4%	4,0%	2,0	2,4	GENE	GAIN	LOSS	AvgSig
	1q							COX2	95%	0%	5,0
	8p							DBC2	0%	100%	2,0
	8q							MYC	100%	0%	10,0
	11q							CCND1	0%	4%	4,0
	16q							CDH1	0%	100%	2,0
	17p							TP53	0%	98%	3,0
	17q							HER2	91%	0%	4,9
	20q							ZNF217	1%	0%	4,0

O

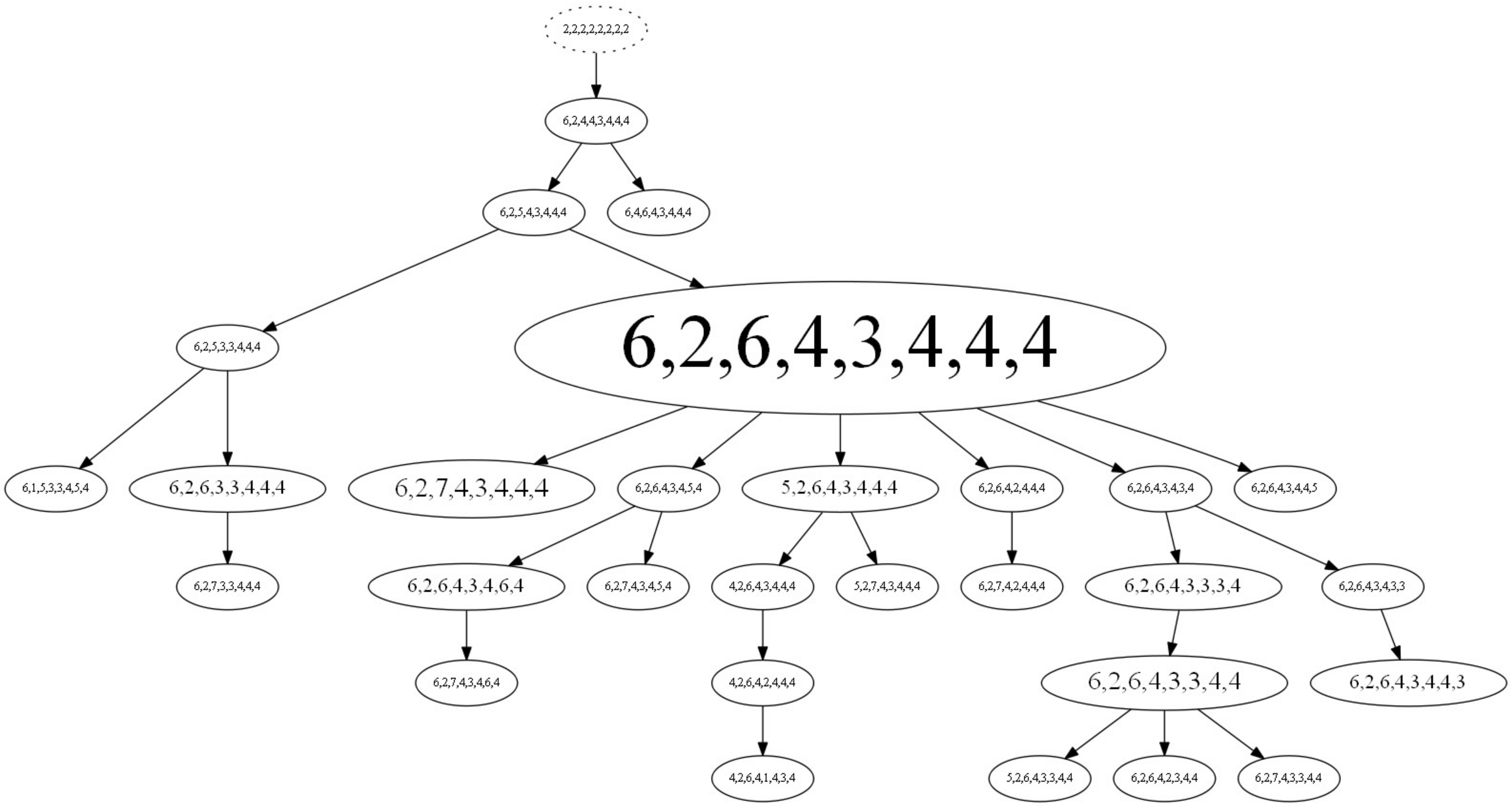

16S	Locus	250 nuclei					Instability Index: 11.6				Average ploidy: 4.0
		82,0%	4,8%	3,2%	2,8%	2,0	5,2%	GENE	GAIN	LOSS	AvgSig
	1q							COX2	99%	0%	5,9
	8p							DBC2	0%	100%	2,0
	8q							MYC	99%	0%	6,1
	11q							CCND1	0%	3%	4,0
	16q							CDH1	0%	100%	3,0
	17p							TP53	0%	6%	3,9
	17q							HER2	4%	2%	4,0
	20q							ZNF217	0%	2%	4,0

P

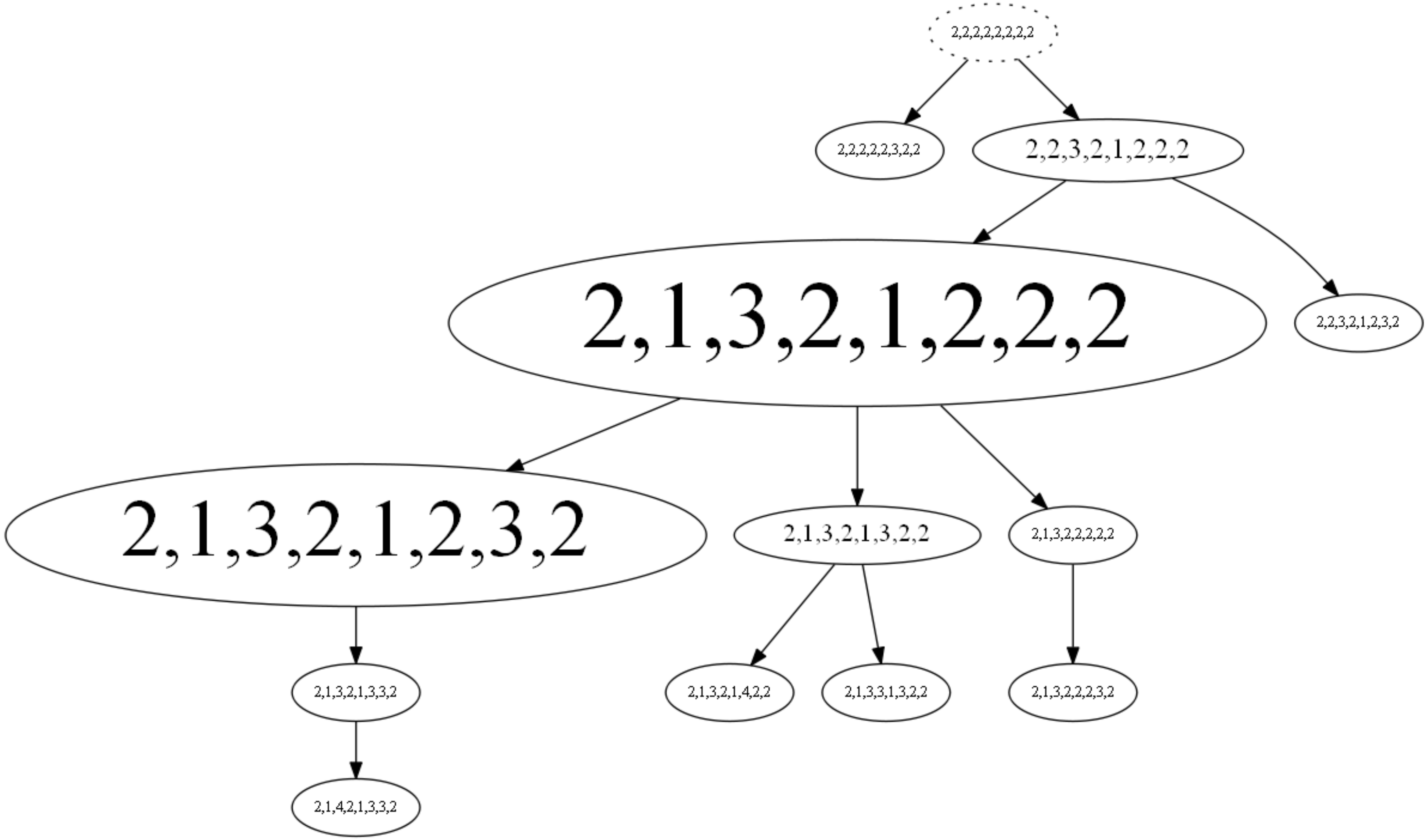

17S	Locus	250 nuclei			Instability Index: 4.8				Average ploidy: 2.0
		78,8%	13,6%	2,4	5,2%	GENE	GAIN	LOSS	AvgSig
	1q					COX2	0%	0%	2,0
	8p					DBC2	0%	96%	1,0
	8q					MYC	100%	0%	3,0
	11q					CCND1	0%	0%	2,0
	16q					CDH1	0%	99%	1,0
	17p					TP53	4%	0%	2,0
	17q					HER2	16%	0%	2,2
	20q					ZNF217	0%	0%	2,0

Q

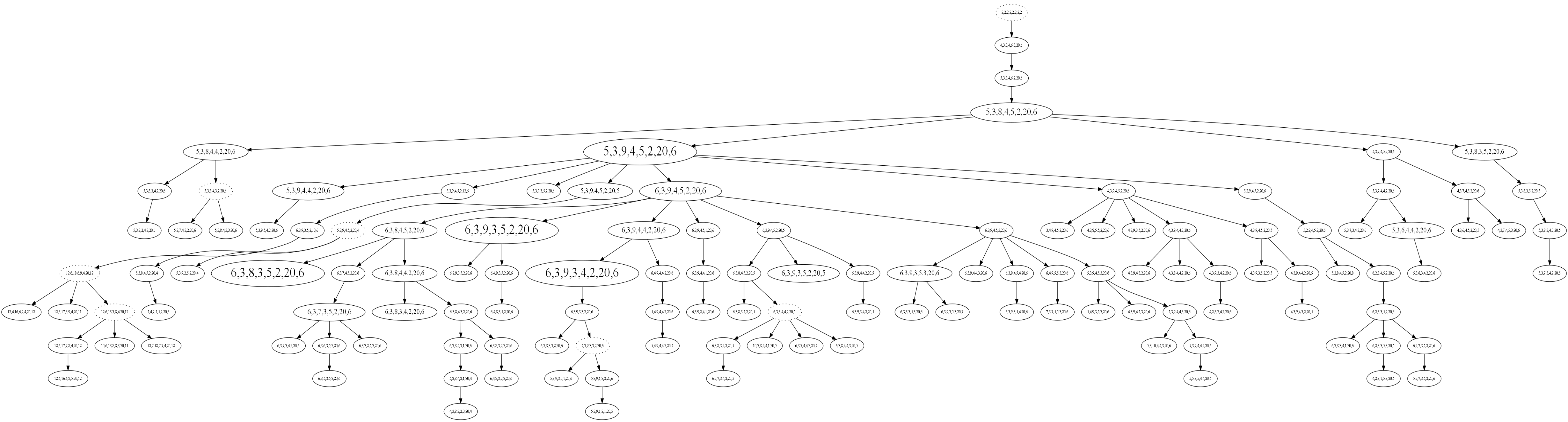

18S	Locus	250 nuclei						Instability Index: 60.0	Average ploidy: 4.1			
		30,4%	21,6%	14,8%	12,0%	3,2%	3,2%	14,8%	GENE	GAIN	LOSS	AvgSig
	1q								COX2	91%	0%	5,6
	8p								DBC2	0%	95%	3,0
	8q								MYC	100%	0%	8,6
	11q								CCND1	2%	50%	3,6
	16q								CDH1	61%	7%	4,6
	17p								TP53	0%	98%	2,1
	17q								HER2	100%	0%	36,5
	20q								ZNF217	98%	0%	6,0

Supplemental Figure S2
