# Supplementary material for: Single Cell Genetic Profiling of Tumors of Breast Cancer Patients Aged 50 Years and Older Reveals Enormous Intratumor Heterogeneity Independent of Individual Prognosis
Source: Cancers (Basel). 2021 Jul 5;13(13):3366. doi: 10.3390/cancers13133366 (PMC8267950; doi:10.3390/cancers13133366)
Supplement: Supplementary file 1 [file cancers-13-03366-s001.zip › cancers-1245840-SI/Supplementary_Files/Supplemental Figures/Supplemental Figure S6.pdf]

A

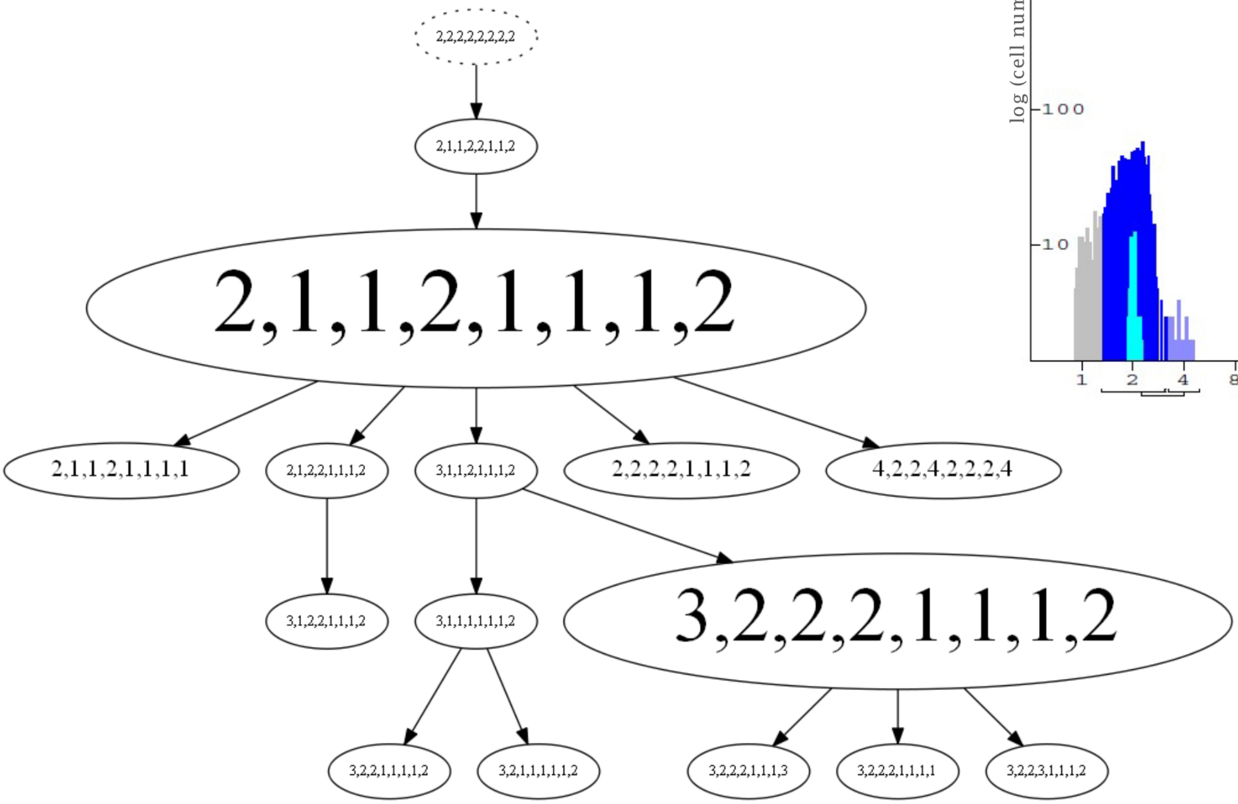

B

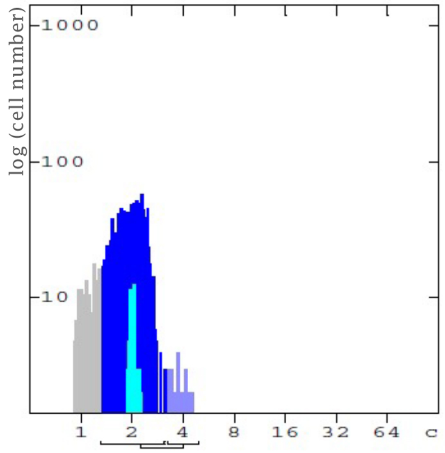

C

13S

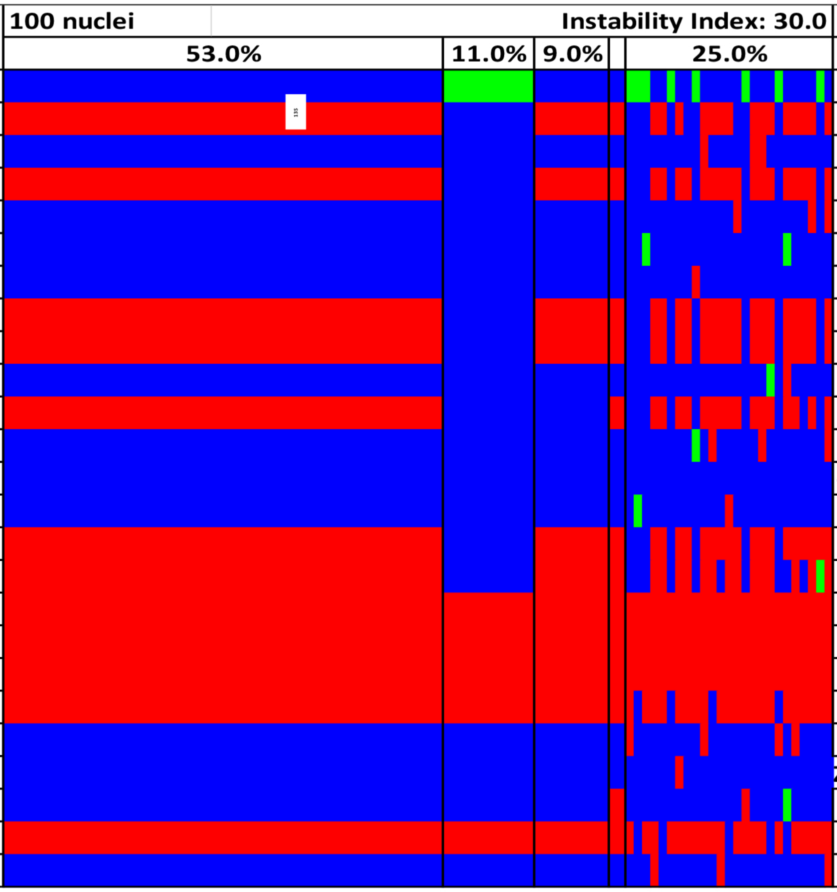

Average ploidy 1.6

Locus	GENE	GAIN	LOSS	AvgSig
1q	COX2	19%	0%	2.2
cen2	CCP2	0%	79%	1.2
cen3	CCP3	0%	3%	2.0
cen4	CCP4	0%	81%	1.2
5q	CCNB1	0%	3%	2.0
cen6	CCP6	2%	0%	2.0
cen7	CCP7	0%	1%	2.0
8p	DBC2	0%	81%	1.2
8q	MYC	0%	81%	1.2
cen9	CCP9	1%	1%	2.0
cen10	CCP10	0%	71%	1.3
cen11	CCP11	1%	3%	2.0
11q	CCND1	0%	0%	2.0
cen12	CCP12	1%	1%	2.0
13q	RB1	0%	82%	1.2
cen15	CCP15	1%	78%	1.2
16q	CDH1	0%	100%	1.0
17p	TP53	0%	100%	1.0
17q	HER2	0%	100%	1.0
cen18	CCP18	0%	96%	1.0
19q	CCNE1	0%	4%	2.0
20q	ZNF217	0%	1%	2.0
21q	DSCR8	1%	3%	2.0
22q	NF2	0%	95%	1.1
cenX	CCPX	0%	3%	2.0

Supplemental Figure S6
