# Supplementary material for: Single Cell Genetic Profiling of Tumors of Breast Cancer Patients Aged 50 Years and Older Reveals Enormous Intratumor Heterogeneity Independent of Individual Prognosis
Source: Cancers (Basel). 2021 Jul 5;13(13):3366. doi: 10.3390/cancers13133366 (PMC8267950; doi:10.3390/cancers13133366)
Supplement: Supplementary file 1 [file cancers-13-03366-s001.zip › cancers-1245840-SI/Supplementary_Files/Supplemental Tables/S1-4 Supplemental Tables.pdf]

Supplemental Tables S1-4

Supplemental Table S1. Clinicopathological features of each sample of the breast cancer cohort (n=39) separated into the group "long survival patients versus short survival patients" and sorted by sample ID with corresponding p-values.

Table S1.		Clinical data											
	Sample ID	Age at diagnosis (years)	Survival (years)	pT-status	pN-status	pM-status	ER-status	PR-status	Her2neu-status	Ki67 (% stained cells)	Intrinsic subtype	Ploidy (by FISH)	Av. Ploidy (by FISH)
Long Survival Samples	1L	55	19.1	pT2	pN0	pM0	pos	pos	neg	< 5	Luminal A	diploid	2
	2L	55	19.6	ND	ND	pM0	pos	pos	neg	< 5	Luminal A	diploid	2.1
	3L	56	20.5	ND	ND	pM0	pos	pos	neg	20	Luminal B, HER2 negative	aneuploid	4.6
	4L	68	19.5	ND	ND	pM0	pos	pos	neg	< 5	Luminal A	aneuploid	2.6
	5L	73	15.7	pT2	pN0	pM0	pos	pos	neg	< 5	Luminal A	aneuploid	4
	6L	78	17.0	pT4	ND	pM0	neg	neg	neg	< 5	Triple negative	aneuploid	4
	7L	66	19.3	pT1	pN0	pM0	pos	neg	neg	< 5	Luminal A	diploid	2
	8L	70	20.5	pT2	pN0	pM0	neg	neg	pos	< 5	Her2 positive	aneuploid	3.2
	9L	77	13.2	pT2	pN1	pM0	pos	neg	neg	< 5	Luminal A	aneuploid	4
	10L	85	13.9	pT1	pN1	pM0	pos	pos	neg	5	Luminal A	diploid	2.1
	11L	71	19.6	pT2	pN1	pM0	pos	pos	pos	5	Luminal B, HER2 positive	diploid	2.1
	12L	60	19.5	pT2	pN2	pM0	neg	neg	neg	< 5	Triple negative	aneuploid	2.5
	13L	55	20.5	pT1	pN1	pM0	neg	neg	neg	< 5	Triple negative	aneuploid	3.1
	14L	61	17.6	pT2	pN0	pM0	neg	neg	pos	< 5	Her2 positive	aneuploid	3
	15L	72	20.5	pT4	ND	pM0	pos	pos	neg	10	Luminal A	aneuploid	3
	16L	71	20.6	pT1	pN0	pM0	pos	ND	neg	< 5	Luminal A	diploid	2
	17L	56	20.7	pT2	pN1	pM0	pos	ND	neg	< 5	Luminal A	diploid	2.1
	18L	59	21.5	pT2	pN1	pM0	pos	pos	neg	< 5	Luminal A	diploid	2
	19L	51	20.5	pT2	pN1	pM0	pos	pos	neg	5	Luminal A	diploid	2
	20L	50	21.5	pT1	pN0	pM0	pos	neg	neg	< 5	Luminal A	aneuploid	4
	21L	51	19.5	pT2	pN1	pM0	pos	pos	neg	5	Luminal A	diploid	2.1
Short Survival Samples	1S	59	3.3	pT2	pN0	M0	neg	neg	pos	< 5	Her2 positive	aneuploid	4
	2S	81	1.6	pT2	pN0	M0	neg	neg	neg	60	Triple negative	aneuploid	3.3
	3S	74	2.1	ND	ND	M0	pos	ND	neg	5	Luminal A	diploid	2
	4S	67	0.8	ND	ND	M0	pos	pos	neg	< 5	Luminal A	aneuploid	4
	5S	82	3.0	pT4	pN1	pM0	pos	pos	neg	5	Luminal A	aneuploid	2.3
	6S	55	0.2	ND	pN1	pM0	neg	neg	neg	5	Triple negative	diploid	2.1
	7S	82	2.0	ND	ND	pM0	pos	pos	neg	5	Luminal A	aneuploid	3
	8S	53	2.9	pT3	pN1	pM0	pos	pos	neg	20	Luminal B, HER2 negative	diploid	2
	9S	62	0.5	pT2	pN1	pM0	neg	neg	neg	80	Triple negative	aneuploid	3
	10S	73	3.4	pT4	ND	pM0	pos	pos	neg	< 5	Luminal A	aneuploid	4
	11S	78	1.6	pT4	pN1	pM0	neg	neg	neg	40	Triple negative	aneuploid	3
	12S	77	1.8	pT4	pN1	pM0	pos	pos	neg	5	Luminal A	aneuploid	4
	13S	58	1	pT2	pN1	pM1	pos	pos	neg	< 5	Luminal A	diploid	2
	14S	58	1.2	pT2	pN1	pM1	neg	pos	pos	10	Luminal B, HER2 positive	diploid	2
	15S	69	4.8	pT4	pN0	pM0	pos	pos	pos	< 5	Luminal B, HER2 positive	aneuploid	4
	16S	84	3.7	ND	ND	pM0	pos	pos	neg	< 5	Luminal A	aneuploid	4
	17S	84	4.5	pT2	pN2	pM0	pos	pos	neg	< 5	Luminal A	diploid	2
	18S	50	4.1	pT3	pN1	pM0	neg	neg	pos	< 5	HER2 positive	aneuploid	4.1

	av. age at diagnosis (years)	av. Survival (years)	pT-status	pN-status	pM-status	ER-status	PR-status	Her2neu-status	Ki67	Intrinsic subtype
Overview Long Survival	63.8	19.0	16 T1/2, 2 T3/4, 3 ND	7 pN0, 9 pN1/2, 5 ND	21 pM0	16/21	11/19	3/21	20 low (0-20%), 1 high (>20%)	14 Luminal A, 2 Luminal B, 3 Triple negative, 2 Her2 positive
Overview Short Survival	69.2	2.4	6 T1/2, 7 T3/4, 5 ND	3 pN0, 10 pN1/2, 5 ND	16 pM0, 2 pM1	11/18	11/17	4/18	14 low (0-20%), 4 high (>20%)	9 Luminal A, 3 Luminal B, 4 Triple negative, 2 Her2 positive
p-value calculation	av. age vs av. age		T1/T2 vs T3/T4	N0 vs N1/N2	pM0 vs pM1	ER-status neg vs pos	PR-status neg vs pos	HER2/neu-status neg vs pos	Ki67 expression low vs high	Intrinsic subtype
p-values	0.135[2]		0.017[1]	0.0434[1]	0.207[1]	0.488[1]	0.742[1]	0.683[1]	0.162[1]	Luminal A 0.342[1]<br>Luminal B 0.646[1]<br>Triple neg 0.683[1]<br>Her2positive 1[1]

[1] Fisher exact test: [2] Student t test

ND, not determined; av., average; vs, versus.

Supplemental Table S2. Clinicopathological features of each sample of the breast cancer cohort (n=39) separated into the group "diploid versus aneuploid samples" and sorted by survival time with corresponding p-values.

Table S2.		Clinical data										
	Sample ID	Age at diagnosis (years)	Survival (years)	pT-status	pN-status	pM-status	ER-status	PR-status	Her2neu-status	Ki67 (% stained cells)	Intrinsic subtype	
Diploid Samples	1L	55	19.1	pT2	pN0	pM0	pos	pos	neg	< 5	Luminal A	Long Survival
	2L	55	19.6	ND	ND	pM0	pos	pos	neg	< 5	Luminal A	
	7L	66	19.3	pT1	pN0	pM0	pos	neg	neg	< 5	Luminal A	
	10L	85	13.9	pT1	pN1	pM0	pos	pos	neg	5	Luminal A	
	11L	71	19.6	pT2	pN1	pM0	pos	pos	pos	5	Luminal B, HER2 positive	
	16L	71	20.5	pT1	pN0	pM0	pos	ND	neg	< 5	Luminal A	
	17L	56	20.6	pT2	pN1	pM0	pos	ND	neg	< 5	Luminal A	
	18L	59	21.5	pT2	pN1	pM0	pos	pos	neg	< 5	Luminal A	
	19L	51	20.5	pT2	pN1	pM0	pos	pos	neg	5	Luminal A	
	21L	51	19.5	pT2	pN1	pM0	pos	pos	neg	5	Luminal A	
	3S	74	2.1	ND	ND	pM0	pos	ND	neg	5	Luminal A	Short Survival
	6S	55	0.2	ND	pN1	pM0	neg	neg	neg	5	Triple negative	
	8S	53	2.9	pT3	pN1	pM0	pos	pos	neg	20	Luminal B, HER2 negative	
	13S	58	1	pT2	pN1	pM1	pos	pos	neg	< 5	Luminal A	
	14S	58	1.2	pT2	pN1	pM1	neg	pos	pos	10	Luminal B, HER2 positive	
	17S	84	4.5	pT2	pN2	pM0	pos	pos	neg	< 5	Luminal A	
Aneuploid Samples	3L	56	20.5	ND	ND	pM0	pos	pos	neg	20	Luminal B, HER2 negative	Long Survival
	4L	68	19.5	ND	ND	pM0	pos	pos	neg	< 5	Luminal A	
	5L	73	15.7	pT2	pN0	pM0	pos	pos	neg	< 5	Luminal A	
	6L	78	17.0	pT4	ND	pM0	neg	neg	neg	< 5	Triple negative	
	8L	70	20.5	pT2	pN0	pM0	neg	neg	pos	< 5	Her2 positive	
	9L	77	13.2	pT2	pN1	pM0	pos	neg	neg	< 5	Luminal A	
	12L	60	19.5	pT2	pN2	pM0	neg	neg	neg	< 5	Triple negative	
	13L	55	20.5	pT1	pN1	pM0	neg	neg	neg	< 5	Triple negative	
	14L	61	17.6	pT2	pN0	pM0	neg	neg	pos	< 5	Her2 positive	
	15L	72	20.5	pT4	ND	pM0	pos	pos	neg	10	Luminal A	
	20L	50	21.5	pT1	pN0	pM0	pos	neg	neg	< 5	Luminal A	
	1S	59	3.3	pT2	pN0	pM0	neg	neg	pos	< 5	Her2 positive	Short Survival
	2S	81	1.6	pT2	pN0	pM0	neg	neg	neg	60	Triple negative	
	4S	67	0.8	ND	ND	pM0	pos	pos	neg	< 5	Luminal A	
	5S	82	3.0	pT4	pN1	pM0	pos	pos	neg	5	Luminal A	
	7S	82	2.0	ND	ND	pM0	pos	pos	neg	5	Luminal A	
	9S	62	0.5	pT2	pN1	pM0	neg	neg	neg	80	Triple negative	
	10S	73	3.4	pT4	ND	pM0	pos	pos	neg	< 5	Luminal A	
	11S	78	1.6	pT4	pN1	pM0	neg	neg	neg	40	Triple negative	
	12S	77	1.8	pT4	pN1	pM0	pos	pos	neg	5	Luminal A	
	15S	69	4.8	pT4	pN0	pM0	pos	pos	pos	< 5	Luminal B, HER2 positive	
	16S	84	3.7	ND	ND	pM0	pos	pos	neg	< 5	Luminal A	
	18S	50	4.1	pT3	pN1	pM0	neg	neg	pos	< 5	HER2 positive	

	av. age at diagnosis (years)	av. survival (years)	pT-status	pN-status	pM-status	ER-status	PR-status	Her2neu-status	Ki67	Intrinsic subtype
Overview Diploid Samples	62.6	12.8	12 T1/2, 1 T3/4, 3 ND	3 pN0, 11 pN1/2, 3 DN	14 pM0, 2 pM1	14/16	11/13	2/16	14 low (0-20%), 1 high (>20%)	12 Luminal A, 3 Luminal B, 1 Triple negative
Average Aneuploid Samples	68.9	10.3	10 T1/2, 8 T3/T4, 5 ND	7 pN0, 8 pN1/2, 8 ND	23 pM0	13/23	11/23	5/23	20 low (0-20%), 4 high (>20%)	11 Luminal A, 2 Luminal B, 6 Triple negative, 4 Her2 positive
p-value calculation	av. age vs av. age	av. survival (years)	T1/T2 vs T3/T4	N0 vs N1/N2	pM0 vs pM1	ER-status neg vs pos	PR-status neg vs pos	HER2/neu-status neg vs pos	Ki67 expression low vs high	Intrinsic subtype
p-values	0.088[2]	0.734[2]	0.045[1]	0.245[1]	0.162[1]	0.076[1]	0.039[1]	0.678[1]	0.631[1]	Luminal A 0.111[1]<br>Luminal B 0.385[1]<br>Triple neg 0.206[1]<br>Her2positive 0.129[1]

[1] Fisher exact test; [2] Student t test

ND, not determined; av., average; vs, versus.

Supplemental Table S3. Clinicopathological features of each sample of the breast cancer cohort (n=39) separated into the group "samples with a low instability index versus samples with a high instability index" and sorted by ploidy with corresponding p-values.

Table S3.		Clinical data											
	Sample ID	Age at diagnosis (years)	Survival (years)	pT-status	pN-status	pM-status	ER-status	PR-status	Her2neu-status	Ki67 (% stained cells)	Intrinsic subtype	Ploidy (by FISH)	
Low Instability Index (<25) Samples	7L	66	19.3	pT1	pN0	pM0	pos	neg	neg	< 5	Luminal A	diploid	Diploid Samples
	17S	84	4.5	pT2	pN2	pM0	pos	pos	neg	< 5	Luminal A	diploid	
	16L	71	20.5	pT1	pN0	pM0	pos	ND	neg	< 5	Luminal A	diploid	
	18L	59	21.5	pT2	pN1	pM0	pos	pos	neg	< 5	Luminal A	diploid	
	13S	58	1.0	pT2	pN1	pM1	pos	pos	neg	< 5	Luminal A	diploid	
	8S	53	2.9	pT3	pN1	pM0	pos	pos	neg	20	Luminal B, HER2 negative	diploid	
	19L	51	20.5	pT2	pN1	pM0	pos	pos	neg	5	Luminal A	diploid	
	2L	55	19.6	ND	ND	pM0	pos	pos	neg	< 5	Luminal A	diploid	
	1L	55	19.1	pT2	pN0	pM0	pos	pos	neg	< 5	Luminal A	diploid	
	14S	58	1.2	pT2	pN1	pM1	neg	pos	pos	10	Luminal B, HER2 positive	diploid	
	3S	74	2.1	ND	ND	pM0	pos	ND	neg	5	Luminal A	diploid	
	21L	51	19.5	pT2	pN1	pM0	pos	pos	neg	5	Luminal A	diploid	
	9S	62	0.5	pT2	pN1	pM0	neg	neg	neg	80	Triple negative	aneuploid	Aneuploid Samples
	16S	84	3.7	ND	ND	pM0	pos	pos	neg	< 5	Luminal A	aneuploid	
	9L	77	13.2	pT2	pN1	pM0	pos	neg	neg	< 5	Luminal A	aneuploid	
	15S	69	4.8	pT4	pN0	pM0	pos	pos	pos	< 5	Luminal B, HER2 positive	aneuploid	
	20L	50	21.5	pT1	pN0	pM0	pos	neg	neg	< 5	Luminal A	aneuploid	
	1S	59	3.3	pT2	pN0	pM0	neg	neg	pos	< 5	Her2 positive	aneuploid	
	6L	78	17.0	pT4	ND	pM0	neg	neg	neg	< 5	Triple negative	aneuploid	
	12S	77	1.8	pT4	pN1	pM0	pos	pos	neg	5	Luminal A	aneuploid	
High Instability Index (>25) Samples	11L	71	19.6	pT2	pN1	pM0	pos	pos	pos	5	Luminal B, HER2 positive	diploid	Diploid
	6S	55	0.2	ND	pN1	pM0	neg	neg	neg	5	Triple negative	diploid	
	10L	85	13.9	pT1	pN1	pM0	pos	pos	neg	5	Luminal A	diploid	
	17L	56	20.5	pT2	pN1	pM0	pos	ND	neg	< 5	Luminal A	diploid	
	14L	61	17.6	pT2	pN0	pM0	neg	neg	pos	< 5	Her2 positive	aneuploid	Aneuploid Samples
	5S	82	3.0	pT4	pN1	pM0	pos	pos	neg	5	Luminal A	aneuploid	
	4L	68	19.5	ND	ND	pM0	pos	pos	neg	< 5	Luminal A	aneuploid	
	13L	55	20.5	pT1	pN1	pM0	neg	neg	neg	< 5	Triple negative	aneuploid	
	5L	73	15.7	pT2	pN0	pM0	pos	pos	neg	< 5	Luminal A	aneuploid	
	2S	81	1.6	pT2	pN0	pM0	neg	neg	neg	60	Triple negative	aneuploid	
	11S	78	1.6	pT4	pN1	pM0	neg	neg	neg	40	Triple negative	aneuploid	
	7S	82	2.0	ND	ND	pM0	pos	pos	neg	5	Luminal A	aneuploid	
	4S	67	0.8	ND	ND	pM0	pos	pos	neg	< 5	Luminal A	aneuploid	
	18S	50	4.1	pT3	pN1	pM0	neg	neg	pos	< 5	HER2 positive	aneuploid	
	12L	60	19.5	pT2	pN2	pM0	neg	neg	neg	< 5	Triple negative	aneuploid	
	10S	73	3.4	pT4	ND	pM0	pos	pos	neg	< 5	Luminal A	aneuploid	
	3L	56	20.5	ND	ND	pM0	pos	pos	neg	20	Luminal B, HER2 negative	aneuploid	
	8L	70	20.5	pT2	pN0	pM0	neg	neg	pos	< 5	Her2 positive	aneuploid	
	15L	72	20.5	pT4	ND	pM0	pos	pos	neg	10	Luminal A	aneuploid	

	av. age at diagnosis (years)	av. Survival (years)	pT-status	pN-status	pM-status	ER-status	PR-status	Her2neu-status	Ki67	Intrinsic subtype
Overview low Instability Index	64.6	10.9	13 pT1/2, 4 pT3/4, 3 ND	6 pN0, 10 pN1, 4 ND	18 pM0, 2 pM1	16/20	12/18	3/20	2 high (>20%), 18 low (0-20%)	14 Luminal A, 3 Luminal B, 2 Triple negative, 1 Her2 positive
Overview high Instability Index	68.2	11.8	9 pT1/2, 5 pT3/4, 5 ND	4 pN0, 9 pN1/2, 6 ND	19 pM0	11/19	10/18	4/19	3 high (>20%), 16 low (0-20%)	9 Luminal A, 2 Luminal B, 5 Triple negative, 3 Her2 positive
p-value calculation	av. age	av. survival (years)	T1/T2 vs T3/T4	N0 vs N1/N2	pM0 vs pM1	ER-status neg vs pos	PR-status neg vs pos	HER2/neu-status neg vs pos	Ki67 expression low vs high	Intrinsic subtype
p-values	0.315[2]	0.734[2]	0.693[1]	1[1]	0.487[1]	0.176[1]	0.733[1]	0.695[1]	0.661[1]	Luminal A 0.2[1]<br>Luminal B 1[1]<br>Triple neg 0.235[1]<br>Her2positive 0.342[1]

[1] Fisher exact test; [2] Student t test

ND, not determined; av., average; vs, versus.

Supplemental Table S4. Clinicopathological features of each sample of the breast cancer cohort (n=39) separated into the groups "lumina A/B versus HER2 positive versus triple negative" and sorted by survival time and sample ID with corresponding p-values.

Table S4.		Clinical data											
Sample ID		Age at diagnosis (years)	Survival (years)	pT-status	pN-status	pM-status	ER-status	PR-status	Her2neu-status	Ki67 (% stained cells)	Intrinsic subtype	Ploidy (by FISH)	Av. Ploidy (by FISH)
Luminal A/B	1L	55	19.1	pT2	pN0	pM0	pos	pos	neg	< 5	Luminal A	diploid	2
	2L	55	19.6	ND	ND	pM0	pos	pos	neg	< 5	Luminal A	diploid	2.1
	4L	68	19.5	ND	ND	pM0	pos	pos	neg	< 5	Luminal A	aneuploid	2.6
	5L	73	15.7	pT2	pN0	pM0	pos	pos	neg	< 5	Luminal A	aneuploid	4
	7L	66	19.3	pT1	pN0	pM0	pos	neg	neg	< 5	Luminal A	diploid	2
	9L	77	13.2	pT2	pN1	pM0	pos	neg	neg	< 5	Luminal A	aneuploid	4
	10L	85	13.9	pT1	pN1	pM0	pos	pos	neg	5	Luminal A	diploid	2.1
	15L	72	20.5	pT4	ND	pM0	pos	pos	neg	10	Luminal A	aneuploid	3
	16L	71	20.6	pT1	pN0	pM0	pos	ND	neg	< 5	Luminal A	diploid	2
	17L	56	20.7	pT2	pN1	pM0	pos	ND	neg	< 5	Luminal A	diploid	2.1
	18L	59	21.5	pT2	pN1	pM0	pos	pos	neg	< 5	Luminal A	diploid	2
	19L	51	20.5	pT2	pN1	pM0	pos	pos	neg	5	Luminal A	diploid	2
	20L	50	21.5	pT1	pN0	pM0	pos	neg	neg	< 5	Luminal A	aneuploid	4
	21L	51	19.5	pT2	pN1	pM0	pos	pos	neg	5	Luminal A	diploid	2.1
	3S	74	2.1	ND	ND	M0	pos	ND	neg	5	Luminal A	diploid	2
	4S	67	0.8	ND	ND	M0	pos	pos	neg	< 5	Luminal A	aneuploid	4
	5S	82	3	pT4	pN1	pM0	pos	pos	neg	5	Luminal A	aneuploid	2.3
	7S	82	2	ND	ND	pM0	pos	pos	neg	5	Luminal A	aneuploid	3
	10S	73	3.4	pT4	ND	pM0	pos	pos	neg	< 5	Luminal A	aneuploid	4
	12S	77	1.8	pT4	pN1	pM0	pos	pos	neg	5	Luminal A	aneuploid	4
	13S	58	1.00	pT2	pN1	pM1	pos	pos	neg	< 5	Luminal A	diploid	2
	16S	84	3.7	ND	ND	pM0	pos	pos	neg	< 5	Luminal A	aneuploid	4
	17S	84	4.5	pT2	pN2	pM0	pos	pos	neg	< 5	Luminal A	diploid	2
	3L	56	20.5	ND	ND	pM0	pos	pos	neg	20	Luminal B, HER2 negative	aneuploid	4.6
	11L	71	19.6	pT2	pN1	pM0	pos	pos	pos	5	Luminal B, HER2 positive	diploid	2.1
	8S	53	2.9	pT3	pN1	pM0	pos	pos	neg	20	Luminal B, HER2 negative	diploid	2
	14S	58	1.2	pT2	pN1	pM1	neg	pos	pos	10	Luminal B, HER2 positive	diploid	2
	15S	69	4.8	pT4	pN0	pM0	pos	pos	pos	< 5	Luminal B, HER2 positive	aneuploid	4
HER2 pos	8L	70	20.5	pT2	pN0	pM0	neg	neg	pos	< 5	HER2 positive	aneuploid	3.2
	14L	61	17.6	pT2	pN0	pM0	neg	neg	pos	< 5	HER2 positive	aneuploid	3
	1S	59	3.3	pT2	pN0	M0	neg	neg	pos	< 5	HER2 positive	aneuploid	4
	18S	50	4.1	pT3	pN1	pM0	neg	neg	pos	< 5	HER2 positive	aneuploid	4.1
Triple neg	6L	78	17	pT4	ND	pM0	neg	neg	neg	< 5	Triple negative	aneuploid	4
	12L	60	19.5	pT2	pN2	pM0	neg	neg	neg	< 5	Triple negative	aneuploid	2.5
	13L	55	20.5	pT1	pN1	pM0	neg	neg	neg	< 5	Triple negative	aneuploid	3.1
	2S	81	1.6	pT2	pN0	M0	neg	neg	neg	60	Triple negative	aneuploid	3.3
	6S	55	0.2	ND	pN1	pM0	neg	neg	neg	5	Triple negative	diploid	2.1
	9S	62	0.5	pT2	pN1	pM0	neg	neg	neg	80	Triple negative	aneuploid	3
	11S	78	1.6	pT4	pN1	pM0	neg	neg	neg	40	Triple negative	aneuploid	3

ND, not determined; av., average.

	av. age at diagnosis (years)	av. Survival (years)	pT-status	pN-status	pM-status
Overview Luminal A/B	67.0	12.0	15 pT1/2, 6 pT3/4, 7 ND	6 pN0, 13 pN1/2, 9 ND	26 pM0, 2 pM1
Overview HER2 pos	60.0	11.4	3 pT1/2, 1 pT3/4	3 pN0, 1 pN1/2	4 pM0
Overview Triple neg	67.0	8.7	4 pT1/2, 2 pT3/4, 1 ND	1 pN0, 5 pN1/2, 1 ND	7 pM0
p-value calculation	av. age at diagnosis (years)	av. Survival (years)	pT-status	pN-status	pM-status
p-values	0.498[2]	0.675[2]	1[1]	0.191[1]	1[1]

[1] Fisher exact test; [2] One way Anova
