# Supplementary material for: Single Cell Genetic Profiling of Tumors of Breast Cancer Patients Aged 50 Years and Older Reveals Enormous Intratumor Heterogeneity Independent of Individual Prognosis
Source: Cancers (Basel). 2021 Jul 5;13(13):3366. doi: 10.3390/cancers13133366 (PMC8267950; doi:10.3390/cancers13133366)
Supplement: Supplementary file 1 [file cancers-13-03366-s001.zip › cancers-1245840-SI/Supplementary_Files/Supplemental Tables/S11-14 Supplemental Tables.pdf]

Supplemental Tables S11-14

Supplemental Table S11. Results of Next Generation Sequencing with the targeted sequence capture approach OncoVar (coding exons of 563 cancer related genes). Distribution of mutations sorted by chromosomal location that affected genes in at least three samples listed for the groups "long survival patients versus short survival patients" with corresponding p-values.

**Table S11.**	**Long versus Short Survival**				**p-values**	
Gene	Gene location	Short survival samples	Long survival samples	Sum	p-value before MTC	p-value after MTC
SPEN	1p36.21	1/18 (5.6%)	2/21 (9.5%)	3/39 (7.7%)	1[1]	1
SF3B1	2q33.1	0/18 (0%)	3/21 (14.3%)	3/39 (7.7%)	0.239[1]	0.573
PIK3CA	3q26	4/18 (22.2%)	8/21 (38.1%)	12/39 (30.8%)	0.322[1]	0.573
MAP3K1	5q11.2	3/18 (16.7%)	4/21 (19%)	7/39 (17.9%)	1[1]	1
KMT2C	7q36.1	4/18 (22.2%)	2/21 (9.5%)	6/39 (15.4%)	0.381[1]	0.573
CDH1	16q22.1	3/18 (16.7%)	1/21 (4.8%)	4/39 (10.3%)	0.318[1]	0.573
TP53	17p13	5/18 (27.8%)	3/21 (14.3%)	8/39 (20.5%)	0.430[1]	0.573
ITGB2	21q22.3	3/18 (16.7%)	0/21 (0%)	3/39 (7.7%)	0.083[1]	0.573
Mutations per sample		1.3	1.1		0.567[2]	

[1] Fisher exact test; [2] Student t test

MTC, multiple test correction.

Supplemental Table S12. Results of Next Generation Sequencing with the targeted sequence capture approach OncoVar (coding exons of 563 cancer related genes). Distribution of mutations sorted by chromosomal location that affected genes in at least three samples listed for the "diploid versus aneuploid samples" with corresponding p-values.

**Table S12.**	**Diploid versus Aneuploid**				**p-values**	
Gene	Gene location	Diploid samples	Aneuploid samples	Sum	p-value before MTC	p-value after MTC
SPEN	1p36.21	1/16 (6,3%)	2/23 (8.7%)	3/39 (7.7%)	1[1]	1
SF3B1	2q33.1	2/16 (12.5%)	1/23 (4.4%)	3/39 (7.7%)	0.557[1]	1
PIK3CA	3q26	6/16 (37.5%)	6/23 (26.1%)	12/39 (30.8%)	0.498[1]	1
MAP3K1	5q11.2	3/16 (18.8%)	4/23 (17.4%)	7/39 (17.9%)	1[1]	1
KMT2C	7q36.1	1/16 (6,3%)	5/23 (21.7%)	6/39 (15.4%)	0.370[1]	1
CDH1	16q22.1	2/16 (12.5%)	2/23 (8.7%)	4/39 (10.3%)	1[1]	1
TP53	17p13	2/16 (12.5%)	6/23 (26.1%)	8/39 (20.5%)	0.432[1]	1
ITGB2	21q22.3	1/16 (6,3%)	2/23 (8.7%)	3/39 (7.7%)	1[1]	1
Mutations per sample		1.1	1.2		0.768[2]	

[1] Fisher exact test; [2] Student t test

MTC, multiple test correction.

Supplemental Table S13. Results of Next Generation Sequencing with the targeted sequence capture approach OncoVar (coding exons of 563 cancer related genes). Distribution of mutations sorted by chromosomal location that affected genes in at least three samples listed for the groups "samples with a low instability index versus samples with a high instability index" with corresponding p-values.

**Table S13.**	**Low (<25) versus high (>25) Instability Index**				**p-values**	
Gene	Gene location	Samples with low instability index (<25)	Samples with high instability index (>25)	Sum	p-value before MTC	p-value after MTC
SPEN	1p36.21	1/20 (5%)	2/19 (10.5%)	3/39 (7.7%)	0.605[1]	0.968
SF3B1	2q33.1	2/20 (10%)	1/19 (5.3%)	3/39 (7.7%)	1[1]	1
PIK3CA	3q26	9/20 (45%)	3/19 (15.8%)	12/39 (30.8%)	0.082[1]	0.508
MAP3K1	5q11.2	5/20 (25%)	2/19 (10.5%)	7/39 (17.9%)	0.408[1]	0.816
KMT2C	7q36.1	2/20 (10%)	4/19 (21.1%)	6/39 (15.4%)	0.408[1]	0.816
CDH1	16q22.1	2/20 (10%)	2/19 (10.5%)	4/39 (10.3%)	1[1]	1
TP53	17p13	2/20 (10%)	6/19 (31.6%)	8/39 (20.5%)	0.127[1]	0.508
ITGB2	21q22.3	2/20 (10%)	1/19 (5.3%)	3/39 (7.7%)	1[1]	1
Mutations per sample		1.3	1.1		0.650[2]	

[1] Fisher exact test; [2] Student t test

MTC, multiple test correction.

Supplemental Table S14. Results of Next Generation Sequencing with the targeted sequence capture approach OncoVar (coding exons of 563 cancer related genes). Distribution of mutations sorted by chromosomal location that affected genes in at least three samples listed for the groups "luminal A/B versus HER2 positive versus triple negative" with corresponding p-values.

**Table S14.**	**Intrinsic Subtypes**					**p-values**	
Gene	Gene location	Luminal A+B	HER2 pos	Triple neg	Sum	p-value before MTC	p-value after MTC
SPEN	1p36.21	2/28 (7.1%)	1/4 (25%)	0/7 (0%)	3/39 (7.7%)	0.352[1]	0.563
SF3B1	2q33.1	3/28 (10.7%)	0/4 (0%)	0/7 (0%)	3/39 (7.7%)	1[1]	1
PIK3CA	3q26	11/28 (39.3%)	0/4 (0%)	1/7 (14.3%)	12/39 (30.8%)	0.234[1]	0.563
MAP3K1	5q11.2	5/28 (17.9%)	0/4 (0%)	2/7 (28.6%)	7/39 (17.9%)	0.650[1]	0.824
KMT2C	7q36.1	3/28 (10.7%)	0/4 (0%)	3/7 (42.9%)	6/39 (15.4%)	0.081[1]	0.324
CDH1	16q22.1	4/28 (14.3%)	0/4 (0%)	0/7 (0%)	4/39 (10.3%)	0.721[1]	0.824
TP53	17p13	2/28 (7.1%)	2/4 (50%)	4/7 (57.1%)	8/39 (20.5%)	0.0048[1]	**0.0384***
ITGB2	21q22.3	2/28 (7.1%)	1/4 (25%)	0/7 (0%)	3/39 (7.7%)	0.352[1]	0.563
Mutations per sample		1.1	1.0	1.4	1.2	0.731[2]	

[1] Fisher exact test; [2] One way Anova

MTC, multiple test correction.

* Significant difference between Luminal A/B and Triple negative group for *TP53*

Subtype Group 1	Subtype Group 2	p-value before MTC	p-value after MTC
Luminal A/B	HER2 pos	0.066[1]	0.099
Luminal A/B	Triple neg	0.0085[1]	**0.026**
HER2 pos	Triple neg	1[1]	1

[1] Fisher exact test
