# Supplementary material for: Single Cell Genetic Profiling of Tumors of Breast Cancer Patients Aged 50 Years and Older Reveals Enormous Intratumor Heterogeneity Independent of Individual Prognosis
Source: Cancers (Basel). 2021 Jul 5;13(13):3366. doi: 10.3390/cancers13133366 (PMC8267950; doi:10.3390/cancers13133366)
Supplement: Supplementary file 1 [file cancers-13-03366-s001.zip › cancers-1245840-SI/Supplementary_Files/Supplemental Tables/S15-17 Supplemental Tables.pdf]

Supplemental Tables S15-17

Supplemental Table S15. Average signal numbers (ASNs) per miFISH gene marker comprising the breast cancer cohort (n=39) listed for the group "long survival patients versus short survival patients" and sorted by sample ID with corresponding p-values. ASNs represent the calculated average of all signal counts per gene marker of the total number of the 250 analyzed nuclei within the respective sample. For the calculation of p-values the student t-test was used.

Table S15	Sample ID	*COX2*	*DBC1*	*MYC*	*CCND1*	*CDH1*	*TP53*	*HER2*	*ZNF217*
Long Survival Samples	1L	4	2.1	2	2	2	1.1	3.1	2
	2L	2.1	3.1	3.1	2.1	1.1	1.2	2.2	2.2
	3L	6.0	5.3	9.7	11.1	4.5	4.0	8.5	10.3
	4L	2.6	1.3	2.7	10.1	1.4	1.3	3.6	2.6
	5L	8.1	4.2	4.1	3.9	2.8	2.1	4.1	4.7
	6L	6.9	4.0	3.9	3.9	2.0	3.9	3.9	5.0
	7L	2.0	1.0	3.1	2.0	1.0	2.0	2.0	2.0
	8L	6.6	2.2	10.9	3.7	3.1	4.2	56.9	6.6
	9L	5.0	4.0	4.1	11.8	2.0	4.0	3.9	4.0
	10L	3.8	1.2	3.7	2.0	1.0	2.2	2.2	2.2
	11L	2.1	3.3	3.1	3.2	2.2	2.4	10.6	3.0
	12L	2.9	2.7	3.3	2.4	2.2	2.3	2.1	2.6
	13L	5.1	2.2	5.8	2.1	2.5	2.2	2.3	5.0
	14L	5.3	2.0	5.3	3.0	3.0	2.2	30.2	4.0
	15L	3.9	2.2	7.8	3.5	2.1	2.5	7.3	5.4
	16L	2.0	2.0	2.0	2.0	1.0	1.0	2.1	2.0
	17L	2.0	1.1	7.2	2.1	1.1	1.4	3.9	3.0
	18L	4.0	3.0	3.0	2.0	1.0	1.0	2.0	2.0
	19L	4.0	2.0	5.1	2.0	1.0	2.0	2.0	4.0
	20L	4.0	3.8	3.8	3.9	2.0	2.1	19.5	8.8
	21L	2.1	3.9	3.9	2.1	1.8	2.2	2.2	3.0
Short Survival Samples	1S	7.0	3.1	3.0	3.0	4.0	2.1	29.7	4.0
	2S	5.9	1.2	5.2	3.3	3.3	1.4	3.1	3.3
	3S	2.9	1.0	2.0	2.0	2.0	1.0	1.1	3.0
	4S	6.1	3.6	3.7	15.7	3.8	2.1	6.2	3.0
	5S	3.4	1.1	3.6	2.2	1.2	1.2	2.3	2.6
	6S	2.8	2.1	6.2	4.0	2.0	2.1	2.1	3.1
	7S	3.9	2.8	4.6	2.9	2.5	2.2	3.9	4.5
	8S	2.0	1.0	8.1	2.0	1.0	1.0	1.0	6.1
	9S	4.0	4.0	4.0	2.6	2.1	2.0	2.0	2.0
	10S	4.5	3.8	3.9	18.4	1.9	3.9	4.1	3.9
	11S	5.4	2.5	6.5	3.8	2.9	2.3	3.9	3.1
	12S	3.9	3.9	3.9	12.1	2.0	2.0	4.0	4.8
	13S	2.2	1.2	1.2	2.0	1.0	1.0	1.0	2.0
	14S	3.0	3.0	3.0	2.1	2.0	2.1	2.1	2.0
	15S	5.0	2.0	10.0	4.0	2.0	3.0	4.9	4.0
	16S	5.9	2.0	6.1	4.0	3.0	3.9	4.0	4.0
	17S	2.0	1.0	3.0	2.0	1.0	2.0	2.2	2.0
	18S	5.6	3.0	8.6	3.6	4.6	2.1	36.5	6.0

Table S15 / Sample ID	*COX2*	*DBC1*	*MYC*	*CCND1*	*CDH1*	*TP53*	*HER2*	*ZNF217*
Average ASN all samples	4.1	2.5	4.7	4.4	2.1	2.2	7.4	3.8
Average ASN Long Survival	4.0	2.7	4.7	3.9	1.9	2.3	8.3	4.0
Average ASN Short Survival	4.2	2.4	4.8	5.0	2.4	2.1	6.3	3.5
p-values before MTC	0.753	0.350	0.832	0.410	0.211	0.560	0.596	0.397
p-values after MTC	0.832	0.795	0.832	0.795	0.795	0.795	0.795	0.795

ASN, average signal number; MTC, multiple test correction.

(green) copy number gain was detected in more than 15% of the nuclei.

(red) copy number loss was detected in more than 15% of the nuclei.

(yellow) The threshold value of 15% of all nuclei was reached for both a detected copy number gain and for a copy number loss.

Supplemental Table S16. Average signal numbers (ASNs) per miFISH gene marker comprising the breast cancer cohort (n=39) listed for the group "diploid versus aneuploid samples" and sorted by survival time with corresponding p-values. ASNs represent the calculated average of all signal counts per gene marker of the total number of the 250 analyzed nuclei within the respective sample. For the calculation of p-values the student t-test was used.

Table S16	Sample ID	*COX2*	*DBC1*	*MYC*	*CCND1*	*CDH1*	*TP53*	*HER2*	*ZNF217*	
Diploid Samples	1L	4	2.1	2	2	2	1.1	3.1	2	Long Survival
Diploid Samples	2L	2.1	3.1	3.1	2.1	1.1	1.2	2.2	2.2	Long Survival
Diploid Samples	7L	2.0	1.0	3.1	2.0	1.0	2.0	2.0	2.0	Long Survival
Diploid Samples	10L	3.8	1.2	3.7	2.0	1.0	2.2	2.2	2.2	Long Survival
Diploid Samples	11L	2.1	3.3	3.1	3.2	2.2	2.4	10.6	3.0	Long Survival
Diploid Samples	16L	2.0	2.0	2.0	2.0	1.0	1.0	2.1	2.0	Long Survival
Diploid Samples	17L	2.0	1.1	7.2	2.1	1.1	1.4	3.9	3.0	Long Survival
Diploid Samples	18L	4.0	3.0	3.0	2.0	1.0	1.0	2.0	2.0	Long Survival
Diploid Samples	19L	4.0	2.0	5.1	2.0	1.0	2.0	2.0	4.0	Long Survival
Diploid Samples	21L	2.1	3.9	3.9	2.1	1.8	2.2	2.2	3.0	Long Survival
Diploid Samples	3S	2.9	1.0	2.0	2.0	2.0	1.0	1.1	3.0	Short Survival
Diploid Samples	6S	2.8	2.1	6.2	4.0	2.0	2.1	2.1	3.1	Short Survival
Diploid Samples	8S	2.0	1.0	8.1	2.0	1.0	1.0	1.0	6.1	Short Survival
Diploid Samples	13S	2.2	1.2	1.2	2.0	1.0	1.0	1.0	2.0	Short Survival
Diploid Samples	14S	3.0	3.0	3.0	2.1	2.0	2.1	2.1	2.0	Short Survival
Diploid Samples	17S	2.0	1.0	3.0	2.0	1.0	2.0	2.2	2.0	Short Survival
Aneuploid Samples	3L	6.0	5.3	9.7	11.1	4.5	4.0	8.5	10.3	Long Survival
Aneuploid Samples	4L	2.6	1.3	2.7	10.1	1.4	1.3	3.6	2.6	Long Survival
Aneuploid Samples	5L	8.1	4.2	4.1	3.9	2.8	2.1	4.1	4.7	Long Survival
Aneuploid Samples	6L	6.9	4.0	3.9	3.9	2.0	3.9	3.9	5.0	Long Survival
Aneuploid Samples	8L	6.6	2.2	10.9	3.7	3.1	4.2	56.9	6.6	Long Survival
Aneuploid Samples	9L	5.0	4.0	4.1	11.8	2.0	4.0	3.9	4.0	Long Survival
Aneuploid Samples	12L	2.9	2.7	3.3	2.4	2.2	2.3	2.1	2.6	Long Survival
Aneuploid Samples	13L	5.1	2.2	5.8	2.1	2.5	2.2	2.3	5.0	Long Survival
Aneuploid Samples	14L	5.3	2.0	5.3	3.0	3.0	2.2	30.2	4.0	Long Survival
Aneuploid Samples	15L	3.9	2.2	7.8	3.5	2.1	2.5	7.3	5.4	Long Survival
Aneuploid Samples	20L	4.0	3.8	3.8	3.9	2.0	2.1	19.5	8.8	Long Survival
Aneuploid Samples	1S	7.0	3.1	3.0	3.0	4.0	2.1	29.7	4.0	Short Survival
Aneuploid Samples	2S	5.9	1.2	5.2	3.3	3.3	1.4	3.1	3.3	Short Survival
Aneuploid Samples	4S	6.1	3.6	3.7	15.7	3.8	2.1	6.2	3.0	Short Survival
Aneuploid Samples	5S	3.4	1.1	3.6	2.2	1.2	1.2	2.3	2.6	Short Survival
Aneuploid Samples	7S	3.9	2.8	4.6	2.9	2.5	2.2	3.9	4.5	Short Survival
Aneuploid Samples	9S	4.0	4.0	4.0	2.6	2.1	2.0	2.0	2.0	Short Survival
Aneuploid Samples	10S	4.5	3.8	3.9	18.4	1.9	3.9	4.1	3.9	Short Survival
Aneuploid Samples	11S	5.4	2.5	6.5	3.8	2.9	2.3	3.9	3.1	Short Survival
Aneuploid Samples	12S	3.9	3.9	3.9	12.1	2.0	2.0	4.0	4.8	Short Survival
Aneuploid Samples	15S	5.0	2.0	10.0	4.0	2.0	3.0	4.9	4.0	Short Survival
Aneuploid Samples	16S	5.9	2.0	6.1	4.0	3.0	3.9	4.0	4.0	Short Survival
Aneuploid Samples	18S	5.6	3.0	8.6	3.6	4.6	2.1	36.5	6.0	Short Survival

Table S16	Sample ID	*COX2*	*DBC1*	*MYC*	*CCND1*	*CDH1*	*TP53*	*HER2*	*ZNF217*	
	Average ASN all samples	4.1	2.5	4.7	4.4	2.1	2.2	7.4	3.8	**Average**
	Average ASN Diploid Samples	2.7	2.0	3.7	2.2	1.4	1.6	2.6	2.7	
	Average ASN Aneuploid Samples	5.1	2.9	5.4	5.9	2.6	2.6	10.7	4.5	
	p-values before MTC	<0.0001	0.0115	0.0217	0.0013	<0.0001	0.0003	0.0127	0.0008	**p-values**
	p-values after MTC	**<0.0001**	**0.0146**	**0.0217**	**0.0021**	**<0.0001**	**0.0007**	**0.0146**	**0.0015**	

ASN, average signal number; MTC, multiple test correction

copy number gain was detected in more than 15% of the nuclei.

copy number loss was detected in more than 15% of the nuclei.

The threshold value of 15% of all nuclei was reached for both a detected copy number gain and for a copy number loss.

Supplemental Table S17. Average signal numbers (ASNs) per miFISH gene marker comprising the breast cancer cohort (n=39) listed for the group "samples with a low instability index versus samples with a high instability index" and sorted by ploidy with corresponding p-values. ASNs represent the calculated average of all signal counts per gene marker of the total number of the 250 analyzed nuclei within the respective sample. For the calculation of p-values the student t-test was used.

Table S17	Sample ID	*COX2*	*DBC1*	*MYC*	*CCND1*	*CDH1*	*TP53*	*HER2*	*ZNF217*	
Low Instability Index (<25) Samples	7L	2.0	1.0	3.1	2.0	1.0	2.0	2.0	2.0	Diploid Samples
	17S	2.0	1.0	3.0	2.0	1.0	2.0	2.2	2.0	
	16L	2.0	2.0	2.0	2.0	1.0	1.0	2.1	2.0	
	18L	4.0	3.0	3.0	2.0	1.0	1.0	2.0	2.0	
	13S	2.2	1.2	1.2	2.0	1.0	1.0	1.0	2.0	
	8S	2.0	1.0	8.1	2.0	1.0	1.0	1.0	6.1	
	19L	4.0	2.0	5.1	2.0	1.0	2.0	2.0	4.0	
	2L	2.1	3.1	3.1	2.1	1.1	1.2	2.2	2.2	
	1L	4	2.1	2	2	2	1.1	3.1	2	
	14S	3.0	3.0	3.0	2.1	2.0	2.1	2.1	2.0	
	3S	2.9	1.0	2.0	2.0	2.0	1.0	1.1	3.0	
	21L	2.1	3.9	3.9	2.1	1.8	2.2	2.2	3.0	
	9S	4.0	4.0	4.0	2.6	2.1	2.0	2.0	2.0	Aneuploid Samples
	16S	5.9	2.0	6.1	4.0	3.0	3.9	4.0	4.0	
	9L	5.0	4.0	4.1	11.8	2.0	4.0	3.9	4.0	
	15S	5.0	2.0	10.0	4.0	2.0	3.0	4.9	4.0	
	20L	4.0	3.8	3.8	3.9	2.0	2.1	19.5	8.8	
	1S	7.0	3.1	3.0	3.0	4.0	2.1	29.7	4.0	
	6L	6.9	4.0	3.9	3.9	2.0	3.9	3.9	5.0	
	12S	3.9	3.9	3.9	12.1	2.0	2.0	4.0	4.8	
High Instability Index (>25) Samples	11L	2.1	3.3	3.1	3.2	2.2	2.4	10.6	3.0	Diploid
	6S	2.8	2.1	6.2	4.0	2.0	2.1	2.1	3.1	
	10L	3.8	1.2	3.7	2.0	1.0	2.2	2.2	2.2	
	17L	2.0	1.1	7.2	2.1	1.1	1.4	3.9	3.0	
	14L	5.3	2.0	5.3	3.0	3.0	2.2	30.2	4.0	Aneuploid Samples
	5S	3.4	1.1	3.6	2.2	1.2	1.2	2.3	2.6	
	4L	2.6	1.3	2.7	10.1	1.4	1.3	3.6	2.6	
	13L	5.1	2.2	5.8	2.1	2.5	2.2	2.3	5.0	
	5L	8.1	4.2	4.1	3.9	2.8	2.1	4.1	4.7	
	2S	5.9	1.2	5.2	3.3	3.3	1.4	3.1	3.3	
	11S	5.4	2.5	6.5	3.8	2.9	2.3	3.9	3.1	
	7S	3.9	2.8	4.6	2.9	2.5	2.2	3.9	4.5	
	4S	6.1	3.6	3.7	15.7	3.8	2.1	6.2	3.0	
	18S	5.6	3.0	8.6	3.6	4.6	2.1	36.5	6.0	
	12L	2.9	2.7	3.3	2.4	2.2	2.3	2.1	2.6	
	10S	4.5	3.8	3.9	18.4	1.9	3.9	4.1	3.9	
	3L	6.0	5.3	9.7	11.1	4.5	4.0	8.5	10.3	
	8L	6.6	2.2	10.9	3.7	3.1	4.2	56.9	6.6	
	15L	3.9	2.2	7.8	3.5	2.1	2.5	7.3	5.4	

Table 17	Sample ID	*COX2*	*DBC1*	*MYC*	*CCND1*	*CDH1*	*TP53*	*HER2*	*ZNF217*	
Average ASN all samples		4.1	2.5	4.7	4.4	2.1	2.2	7.4	3.8	**Average**
Average ASN low Instability Index		3.7	2.6	3.9	3.5	1.7	2.0	4.7	3.4	
Average ASN high Instability Index		4.5	2.5	5.6	5.3	2.5	2.3	10.2	4.1	
p-values before MTC		0.129	0.893	0.027	0.167	0.012	0.338	0.156	0.246	**p-values**
p-values after MTC		0.267	0.893	0.107	0.267	0.094	0.386	0.267	0.328	

ASN, average signal number; MTC, multiple test correction

copy number gain was detected in more than 15% of the nuclei.

copy number loss was detected in more than 15% of the nuclei.

The threshold value of 15% of all nuclei was reached for both a detected copy number gain and for a copy number loss.
