# Supplementary material for: Single Cell Genetic Profiling of Tumors of Breast Cancer Patients Aged 50 Years and Older Reveals Enormous Intratumor Heterogeneity Independent of Individual Prognosis
Source: Cancers (Basel). 2021 Jul 5;13(13):3366. doi: 10.3390/cancers13133366 (PMC8267950; doi:10.3390/cancers13133366)
Supplement: Supplementary file 1 [file cancers-13-03366-s001.zip › cancers-1245840-SI/Supplementary_Files/Supplemental Tables/S18-20 Supplemental Tables.pdf]

Supplemental Tables S18-20

Supplemental Table S18. MiFISH results showing the major clone signal-pattern, ploidy measurement results and the calculation of Instability-, Shannon and Simpson-Index plus FISHtree parameters of each sample of the breast cancer cohort (n=39) separated into the group "long survival patients versus short survival patients" and sorted by sample ID with corresponding p-values.

Table S18.		Ploidy results		Diversity Indices			Major signal pattern			FISHtree parameters	
Sample ID		Av. Ploidy (FISH)	Ploidy measurement (FISH)	Instability Index	Shannon Index	Simpson Index	number of cells with major pattern	% cells with major pattern	Major signal patterns COX2-DBC2-MYC-CCND1-CDH1-TP53-HER2-ZNF217	Total number of events in the tree	Tree max. depth
Long Survival Samples	1L	2	diploid	13.2	3.97	0.92	170	68	4-2-2-2-2-1-3-2	61	8
	2L	2.1	diploid	11.2	4.17	0.94	165	66	2-3-3-2-1-1-2-2	55	8
	3L	4.6	aneuploid	72.4	4.12	0.93	19	7.6	6-6-10-12-5-4-9-10	455	23
	4L	2.6	aneuploid	34.8	3.97	0.92	97	38.8	2-1-2-8-1-1-3-2	180	14
	5L	4	aneuploid	46.4	4.04	0.93	71	28.4	8-4-4-4-3-2-4-5	207	15
	6L	4	aneuploid	20.8	4.03	0.93	159	63.6	7-4-4-4-2-4-4-5	89	11
	7L	2	diploid	2	4.24	0.94	217	86.8	2-1-3-2-1-2-2-2	27	4
	8L	3.2	aneuploid	86.4	3.80	0.92	6	2.4	6-2-13-3-3-4-60-7	536	42
	9L	4	aneuploid	12	4.10	0.93	181	72.4	5-4-4-12-2-4-4-4	71	7
	10L	2.1	diploid	33.2	3.61	0.88	69	27.6	4-1-4-2-1-2-2-2	142	11
	11L	2.1	diploid	25.6	3.94	0.92	115	46	2-3-3-3-2-2-10-3	134	12
	12L	2.5	aneuploid	62.8	4.13	0.93	19	7.6	2-2-2-2-1-2-2-2	276	12
	13L	3.1	aneuploid	44.8	4.14	0.94	57	22.8	5-2-6-2-2-2-2-5	249	16
	14L	3	aneuploid	26.4	3.79	0.90	36	14.4	6-2-6-3-3-2-30-4	112	13
	15L	3	aneuploid	86.8	4.00	0.93	6+6	4.8	3-2-9-3-2-2-4-6, 3-2-7-3-2-3-7-6	404	31
	16L	2	diploid	5.2	4.24	0.94	220	88	2-2-2-2-1-1-2-2	36	5
	17L	2.1	diploid	45.2	3.52	0.86	22	8.8	2-1-6-2-1-1-4-3	193	19
	18L	2	diploid	6	4.12	0.93	225	90	4-3-3-2-1-1-2-2	44	7
	19L	2	diploid	10	4.13	0.93	210	84	4-2-5-2-1-2-2-4	56	8
	20L	4	aneuploid	14.8	3.89	0.92	140	56	4-4-4-4-2-2-20-9	84	9
	21L	2.1	diploid	24	4.20	0.94	96	38.4	2-4-4-2-2-2-2-3	104	11
Short Survival Samples	1S	4	aneuploid	17.2	4.10	0.93	162	64.8	7-3-3-3-4-2-30-4	75	10
	2S	3.3	aneuploid	47.6	3.63	0.89	53	21.2	6-1-5-3-3-1-3-3	194	17
	3S	2	diploid	14.4	4.11	0.93	188	75.2	3-1-2-2-2-1-1-3	63	9
	4S	4	aneuploid	54.4	3.60	0.87	39	15.6	6-4-4-16-4-2-6-3	278	23
	5S	2.3	aneuploid	28.4	3.97	0.92	78	31.2	3-1-3-2-1-1-2-2	153	16
	6S	2.1	diploid	27.2	3.98	0.92	87	34.8	3-2-6-4-2-2-2-3	151	12
	7S	3	aneuploid	51.2	3.91	0.91	22	8.8	4-3-5-3-2-2-4-4	197	15
	8S	2	diploid	7.6	4.03	0.92	111+111	88.8	2-1-8-2-1-1-1-5, 2-1-8-2-1-1-1-7	64	6
	9S	3	aneuploid	11.2	4.11	0.93	143	57.2	4-4-4-3-2-2-2-2	56	9
	10S	4	aneuploid	68.8	3.96	0.92	19	7.6	4-4-4-20-2-4-4-4	288	21
	11S	3	aneuploid	50	3.95	0.92	16	6.4	6-3-7-4-3-3-4-3	201	16
	12S	4	aneuploid	24.8	4.02	0.92	84	33.6	4-4-4-10-2-2-4-5	116	21
	13S	2	diploid	6	4.25	0.94	186	74.4	2-1-1-2-1-1-1-2	37	6
	14S	2	diploid	13.6	4.07	0.93	197	78.8	3-3-3-2-2-2-2-2	63	7
	15S	4	aneuploid	12	3.74	0.87	128	51.2	5-2-10-4-2-3-5-4	61	8
	16S	4	aneuploid	11.6	3.96	0.92	182	72.8	6-2-6-4-3-4-4-4	56	8
	17S	2	diploid	4.8	4.14	0.93	197	78.8	2-1-3-2-1-2-2-2	34	6
	18S	4.1	aneuploid	60	3.83	0.91	10+10	8	5-3-9-4-5-2-40-6, 6-3-9-3-5-2-40-6	223	13
Sample ID		av. Ploidy (FISH)	Ploidy measurement (FISH)	av. Instability Index	av. Shannon Index	av. Simpson Index	number of cells with major pattern	% cells with major pattern	Major signal patterns COX2-DBC2-MYC-CCND1-CDH1-TP53-HER2-ZNF217	Total number of events in the tree	Tree max depth
Overview Long Survival		2.8	10 diploid, 11 aneuploid	32.6	4.01	0.92	109.8	ND	ND	167.4	13.6
Overview Short Survival		3.0	6 diploid, 12 aneuploid	28.4	3.96	0.92	112.4	ND	ND	128.3	12.4
p-values before MTC		0.367[2]	0.516[1]	0.582[2]	0.473[2]	0.410[2]	0.889[2]	ND	ND	0.301[2]	0.607[2]
p-values after MTC		0.694	0.694	0.694	0.694	0.694	0.916	ND	ND	0.694	0.694

[1] Fisher exact test; [2] Student t test

ND, not determined, MTC, multiple test correction, av., average.

Supplemental Table S19. MiFISH results showing the major clone signal-pattern, ploidy measurement results and the calculation of Instability-, Shannon and Simpson-Index plus FISHtree parameters of each sample of the breast cancer cohort (n=39) separated into the group "diploid versus aneuploid samples" and sorted by survival time with corresponding p-values (calculated with Student t-test).

Table S19.		Ploidy results		Diversity Indices			Major signal pattern			FISHtree results			
	Sample ID	Av. Ploidy (FISH)	Ploidy results (FISH)	Instability Index	Shannon Index	Simpson Index	number of cells with major pattern	% cells with major pattern	Major signal patterns COX2-DBC2-MYC-CCND1-CDH1-TP53-HER2-ZNF217	Total number of events in the tree	Tree max. depth	Survival (years)	
Diploid Samples	1L	2	diploid	13.2	0.92	3.97	170	68.0	4-2-2-2-2-1-3-2	61	8	19.1	Long Survival
	2L	2.1	diploid	11.2	0.94	4.17	165	66.0	2-3-3-2-1-1-2-2	55	8	19.6	
	7L	2	diploid	2	0.94	4.24	217	86.8	2-1-3-2-1-2-2-2	27	4	19.3	
	10L	2.1	diploid	33.2	0.88	3.61	69	27.6	4-1-4-2-1-2-2-2	142	11	13.9	
	11L	2.1	diploid	25.6	0.92	3.94	115	46.0	2-3-3-3-2-2-10-3	134	12	19.6	
	16L	2	diploid	5.2	0.94	4.24	220	88.0	2-2-2-2-1-1-2-2	36	5	20.5	
	17L	2.1	diploid	45.2	0.86	3.52	22	8.8	2-1-6-2-1-1-4-3	193	19	20.6	
	18L	2	diploid	6	0.93	4.12	225	90.0	4-3-3-2-1-1-2-2	44	7	21.5	
	19L	2	diploid	10	0.93	4.13	210	84.0	4-2-5-2-1-2-2-4	56	8	20.5	
	21L	2.1	diploid	24	0.94	4.20	96	38.4	2-4-4-2-2-2-2-3	104	11	19.5	
	3S	2.0	diploid	14.4	0.93	4.11	188	75.2	3-1-2-2-2-1-1-3	63	9	2.1	Short Survival
	6S	2.1	diploid	27.2	0.92	3.98	87	34.8	3-2-6-4-2-2-2-3	151	12	0.2	
	8S	2.0	diploid	7.6	0.92	4.03	111+111	88.8	2-1-8-2-1-1-1-5, 2-1-8-2-1-1-1-7	64	6	2.9	
	13S	2.0	diploid	6	0.94	4.25	186	74.4	2-1-1-2-1-1-1-2	37	6	1	
	14S	2.0	diploid	13.6	0.93	4.07	197	78.8	3-3-3-2-2-2-2-2	63	7	1.2	
	17S	2.0	diploid	4.8	0.93	4.14	197	78.8	2-1-3-2-1-2-2-2	34	6	4.5	
Aneuploid Samples	3L	4.6	aneuploid	72.4	0.93	4.12	19	7.6	6-6-10-12-5-4-9-10	455	23	20.5	Long Survival
	4L	2.6	aneuploid	34.8	0.92	3.97	97	38.8	2-1-2-8-1-1-3-2	180	14	19.5	
	5L	4	aneuploid	46.4	0.93	4.04	71	28.4	8-4-4-4-3-2-4-5	207	15	15.7	
	6L	4	aneuploid	20.8	0.93	4.03	159	63.6	7-4-4-4-2-4-4-5	89	11	17.0	
	8L	3.2	aneuploid	86.4	0.92	3.80	6	2.4	6-2-13-3-3-4-60-7	536	42	20.5	
	9L	4	aneuploid	12	0.93	4.10	181	72.4	5-4-4-12-2-4-4-4	71	7	13.2	
	12L	2.5	aneuploid	62.8	0.93	4.13	19	7.6	2-2-2-2-1-2-2-2	276	12	19.5	
	13L	3.1	aneuploid	44.8	0.94	4.14	57	22.8	5-2-6-2-2-2-2-5	249	16	20.5	
	14L	3	aneuploid	26.4	0.90	3.79	36	14.4	6-2-6-3-3-2-30-4	112	13	17.6	
	15L	3	aneuploid	86.8	0.93	4.00	6+6	4.8	3-2-9-3-2-2-4-6, 3-2-7-3-2-3-7-6	404	31	20.5	
	20L	4	aneuploid	14.8	0.92	3.89	140	56.0	4-4-4-4-2-2-20-9	84	9	21.5	
	1S	4	aneuploid	17.2	0.93	4.10	162	64.8	7-3-3-3-4-2-30-4	75	10	3.3	Short Survival
	2S	3.3	aneuploid	47.6	0.89	3.63	53	21.2	6-1-5-3-3-1-3-3	194	17	1.6	
	4S	4	aneuploid	54.4	0.87	3.60	39	15.6	6-4-4-16-4-2-6-3	278	23	0.8	
	5S	2.3	aneuploid	28.4	0.92	3.97	78	31.2	3-1-3-2-1-1-2-2	153	16	3.0	
	7S	3	aneuploid	51.2	0.91	3.91	22	8.8	4-3-5-3-2-2-4-4	197	15	2.0	
	9S	3	aneuploid	11.2	0.93	4.11	143	57.2	4-4-4-3-2-2-2-2	56	9	0.5	
	10S	4	aneuploid	68.8	0.92	3.96	19	7.6	4-4-4-20-2-4-4-4	288	21	3.4	
	11S	3	aneuploid	50	0.92	3.95	16	6.4	6-3-7-4-3-3-4-3	201	16	1.6	
	12S	4	aneuploid	24.8	0.92	4.02	84	33.6	4-4-4-10-2-2-4-5	116	21	1.8	
	15S	4	aneuploid	12	0.87	3.74	128	51.2	5-2-10-4-2-3-5-4	61	8	4.8	
	16S	4	aneuploid	11.6	0.92	3.96	182	72.8	6-2-6-4-3-4-4-4	56	8	3.7	
	18S	4.1	aneuploid	60	0.91	3.83	10+10	8.0	5-3-9-4-5-2-40-6, 6-3-9-3-5-2-40-6	223	13	4.1	

Sample ID	av. Ploidy (FISH)	Ploidy measurement (FISH)	av. Instability Index	av. Shannon Index	av. Simpson Index	number of cells with major pattern	% cells with major pattern	Major signal patterns COX2-DBC2-MYC-CCND1-CDH1-TP53-HER2-ZNF217	Total number of events in the tree	Tree max depth
Overview Diploid Samples	2.0	16 diploid	15.6	0.92	4.04	161.6	ND	ND	79.0	8.7
Overview Aneuploid Samples	3.5	23 aneuploid	41.1	0.92	3.95	75.8	ND	ND	198.3	16.1
		p-values before MTC[2]	0.0001	0.349	0.129	0.001	ND	ND	0.0004	0.0006
		p-values after MTC	**0.0006**	0.349	0.154	**0.0006**	ND	ND	**0.0008**	**0.0008**

[1] Fisher exact test; [2] Student t test

ND, not determined, MTC, multiple test correction, av., average, vs, versus.

Supplemental Table S20. MiFISH results showing the major clone signal-pattern, ploidy measurement results and the calculation of Instability-, Shannon and Simpson-Index plus FISHtree parameters of each sample of the breast cancer cohort (n=39) separated into the group "samples with a low instability index versus samples with a high instability index" and sorted by ploidy with corresponding p-values.

Table S20.		Ploidy results			Diversity Indices			Major signal pattern			FISHtree results	
	Sample ID	Av. Ploidy (FISH)	Ploidy (by FISH)		Instability Index	Shannon Index	Simpson Index	number of cells with major pattern	% cells with major pattern	Major signal patterns COX2-DBC2-MYC-CCND1-CDH1-TP53-HER2-ZNF217	Total number of events in the tree	Tree max depth
Low Instability Index (<25) Samples	7L	2	diploid	Diploid Samples	2	4.24	0.94	217	86.8	2-1-3-2-1-2-2-2	27	4
	17S	2	diploid		4.8	4.14	0.93	197	78.8	2-1-3-2-1-2-2-2	34	6
	16L	2	diploid		5.2	4.24	0.94	220	88	2-2-2-2-1-1-2-2	36	5
	18L	2	diploid		6	4.12	0.93	225	90	4-3-3-2-1-1-2-2	44	7
	13S	2	diploid		6	4.25	0.94	186	74.4	2-1-1-2-1-1-1-2	37	6
	8S	2	diploid		7.6	4.03	0.92	111+111	44.4	2-1-8-2-1-1-1-5, 2-1-8-2-1-1-1-7	64	6
	19L	2	diploid		10	4.13	0.93	210	84	4-2-5-2-1-2-2-4	56	8
	2L	2.1	diploid		11.2	4.17	0.94	165	66	2-3-3-2-1-1-2-2	55	8
	1L	2	diploid		13.2	3.97	0.92	170	68	4-2-2-2-2-1-3-2	61	8
	14S	2	diploid		13.6	4.07	0.93	197	78.8	3-3-3-2-2-2-2-2	63	7
	3S	2	diploid		14.4	4.11	0.93	188	75.2	3-1-2-2-2-1-1-3	63	9
	21L	2.1	diploid		24	4.20	0.94	96	38.4	2-4-4-2-2-2-2-3	104	11
	9S	3	aneuploid	Aneuploid Samples	11.2	4.11	0.93	143	57.2	4-4-4-3-2-2-2-2	56	9
	16S	4	aneuploid		11.6	3.96	0.92	182	72.8	6-2-6-4-3-4-4-4	56	8
	9L	4	aneuploid		12	4.10	0.93	181	72.4	5-4-4-12-2-4-4-4	71	7
	15S	4	aneuploid		12	3.74	0.87	128	51.2	5-2-10-4-2-3-5-4	61	8
	20L	4	aneuploid		14.8	3.89	0.92	140	56	4-4-4-4-2-2-20-9	84	9
	1S	4	aneuploid		17.2	4.10	0.93	162	64.8	7-3-3-3-4-2-30-4	75	10
	6L	4	aneuploid		20.8	4.03	0.93	159	63.6	7-4-4-4-2-4-4-5	89	11
	12S	4	aneuploid		24.8	4.02	0.92	84	33.6	4-4-4-10-2-2-4-5	116	21
High Instability Index (>25) Samples	11L	2.1	diploid	Diploid	25.6	3.94	0.92	115	46	2-3-3-3-2-2-10-3	134	12
	6S	2.1	diploid		27.2	3.98	0.92	87	34.8	3-2-6-4-2-2-2-3	151	12
	10L	2.1	diploid		33.2	3.61	0.88	69	27.6	4-1-4-2-1-2-2-2	142	11
	17L	2.1	diploid		45.2	3.52	0.86	22	8.8	2-1-6-2-1-1-4-3	193	19
	14L	3	aneuploid	Aneuploid Samples	26.4	3.79	0.90	36	14.4	6-2-6-3-3-2-30-4	112	13
	5S	2.3	aneuploid		28.4	3.97	0.92	78	31.2	3-1-3-2-1-1-2-2	153	16
	4L	2.6	aneuploid		34.8	3.97	0.92	97	38.8	2-1-2-8-1-1-3-2	180	14
	13L	3.1	aneuploid		44.8	4.14	0.94	57	22.8	5-2-6-2-2-2-2-5	249	16
	5L	4	aneuploid		46.4	4.04	0.93	71	28.4	8-4-4-4-3-2-4-5	207	15
	2S	3.3	aneuploid		47.6	3.63	0.89	53	21.2	6-1-5-3-3-1-3-3	194	17
	11S	3	aneuploid		50	3.95	0.92	16	6.4	6-3-7-4-3-3-4-3	201	16
	7S	3	aneuploid		51.2	3.91	0.91	22	8.8	4-3-5-3-2-2-4-4	197	15
	4S	4	aneuploid		54.4	3.60	0.87	39	15.6	6-4-4-16-4-2-6-3	278	23
	18S	4.1	aneuploid		60	3.83	0.91	10+10	4	5-3-9-4-5-2-40-6, 6-3-9-3-5-2-40-6	223	13
	12L	2.5	aneuploid		62.8	4.13	0.93	19	7.6	2-2-2-2-1-2-2-2	276	12
	10S	4	aneuploid		68.8	3.96	0.92	19	7.6	4-4-4-20-2-4-4-4	288	21
	3L	4.6	aneuploid		72.4	4.12	0.93	19	7.6	6-6-10-12-5-4-9-10	455	23
	8L	3.2	aneuploid		86.4	3.80	0.92	6	2.4	6-2-13-3-3-4-60-7	536	42
	15L	3	aneuploid		86.8	4.00	0.93	6	2.4	3-2-9-3-2-2-4-6, 3-2-7-3-2-3-7-6	404	31
		av. Ploidy (FISH)	Ploidy measurement (FISH)		av. Instability Index	av. Shannon Index	av. Simpson Index	number of cells with major pattern	% cells with major pattern	Major signal patterns COX2-DBC2-MYC-CCND1-CDH1-TP53-HER2-ZNF217	Total number of events in the tree	Tree max depth
Overview low Instability Index		2.8	12 diploid, 8 aneuploid		12.1	4.08	0.93	171.1	ND	ND	62.6	8.4
Overview high Instability Index		3.1	4 diploid, 15 aneuploid		50.1	3.89	0.91	44.3	ND	ND	240.7	17.9
p-values before MTC		0.293[2]	0.023[1]		ND	0.0007[2]	0.014[2]	<0.0001[2]	ND	ND	<0.0001[2]	<0.0001[2]
p-values after MTC		0.293	**0.027**		ND	**0.001**	**0.020**	**<0.0001**	ND	ND	**<0.0001**	**<0.0001**

[1] Fisher exact test; [2] Student t test

ND, not determined, MTC, multiple test correction, av., average.
