# Supplementary material for: Single Cell Genetic Profiling of Tumors of Breast Cancer Patients Aged 50 Years and Older Reveals Enormous Intratumor Heterogeneity Independent of Individual Prognosis
Source: Cancers (Basel). 2021 Jul 5;13(13):3366. doi: 10.3390/cancers13133366 (PMC8267950; doi:10.3390/cancers13133366)
Supplement: Supplementary file 1 [file cancers-13-03366-s001.zip › cancers-1245840-SI/Supplementary_Files/Supplemental Tables/S5-7 Supplemental Tables.pdf]

Supplemental Tables S5-7

Supplemental Table S5. MiFISH results showing copy number gains, losses and alterations per gene marker comprising the whole breast cancer cohort (n=39) listed for the group "long survival patients versus short survival patients" with corresponding p-values. MiFISH gene markers are sorted by chromosomal order. If a gain/ loss of signal counts in one of the analyzed miFISH gene markers occured in ≥15% of all nuclei in the respective sample, a gain/loss of copy number was registered accordingly in the tables.

Table S5.			Long versus Short Survival			p-values	
	Gene	Gene location	All samples	Long survival samples	Short survival samples	p-value before MTC	p-value after MTC
Gains	*COX2*	1p31.1	28/39 (71.8%)	13/21 (61.9%)	15/18 (83.3%)	0.171[1]	0.456
	DBC2	8p21.3	9/39 (23.1%)	7/21 (33.3%)	2/18 (11.1%)	0.139[1]	0.456
	MYC	8q24.21	27/39 (69.2%)	15/21 (71.4%)	12/18 (66.7%)	1[1]	1.000
	CCND1	11q13.3	11/39 (28.2%)	6/21 (28.6%)	5/18 (27.8%)	1[1]	1.000
	CDH1	16q22.1	2/39 (5.1%)	1/21 (4.8%)	1/18 (5.6%)	1[1]	1.000
	TP53	17p13.1	5/39 (12.8%)	4/21 (19%)	1/18 (5.6%)	0.349[1]	0.620
	HER2	17q12	18/39 (46.2%)	10/21 (47.6%)	8/18 (44.4%)	1[1]	1.000
	ZNF217	20q13.2	21/39 (53.8%)	13/21 (61.9%)	8/18 (44.4%)	0.343[1]	0.620
	Gene	Gene location	All samples	Long survival samples	Short survival samples	p-value before MTC	p-value after MTC
Losses	*COX2*	1p31.1	0/39 (0%)	0/21 (0%)	0/18 (0%)	1[1]	1.000
	DBC2	8p21.3	23/39 (59.0%)	9/21 (42.9%)	14/18 (77.8%)	0.049[1]	0.456
	MYC	8q24.21	5/39 (12.8%)	1/21 (4.8%)	4/18 (22.2%)	0.162[1]	0.456
	CCND1	11q13.3	5/39 (12.8%)	2/21 (9.5%)	3/18 (16.7%)	0.647[1]	0.941
	CDH1	16q22.1	29/39 (74.4%)	18/21 (85.7%)	11/18 (61.1%)	0.141[1]	0.456
	TP53	17p13.1	27/39 (69.2%)	13/21 (61.9%)	14/18 (77.8%)	0.322[1]	0.620
	HER2	17q12	8/39 (20.5%)	2/21 (9.5%)	6/18 (33.3%)	0.112[1]	0.456
	ZNF217	20q13.2	3/39 (7.7%)	1/21 (4.8%)	2/18 (11.1%)	0.586[1]	0.938
Ratio	Ratio gains per sample		3.10	3.29	2.89	0.398[2]	0.502
	Ratio losses per sample		2.56	2.19	3.0	0.086[2]	0.259
	Ratio CNA per sample		5.67	5.48	5.89	0.502[2]	0.502

[1] Fisher exact test; [2] Student t test

CNA, copy number alterations; MTC, multiple test correction.

Supplemental Table S6. MiFISH results showing copy number gains, losses and alterations per gene marker comprising the whole breast cancer cohort (n=39) listed for the group "diploid versus aneuploid samples" with corresponding p-values. MiFISH gene markers are sorted by chromosomal order. If a gain/ loss of signal counts in one of the analyzed miFISH gene markers occured in ≥15% of all nuclei in the respective sample, a gain/loss of copy number was registered accordingly in the tables.

**Table S6.**			**Diploid versus Aneuploid Samples**			**p-values**	
**Gains**	Gene	Gene location	All Samples	Diploid Samples	Aneuploid Samples	p-value before MTC	p-value after MTC
	COX2	1p31.1	28/39 (71.8%)	8/16 (50%)	20/23 (87%)	0.027[1]	0.328
	DBC2	8p21.3	9/39 (23.1%)	5/16 (31.3%)	4/23 (17.4%)	0.444[1]	0.710
	MYC	8q24.21	27/39 (69.2%)	12/16 (75%)	15/23 (65.2%)	0.726[1]	0.830
	CCND1	11q13.3	11/39 (28.2%)	2/16 (12.5%)	9/23 (39.1%)	0.086[1]	0.344
	CDH1	16q22.1	2/39 (5.1%)	0/16 (0%)	2/23 (8.7%)	0.503[1]	0.732
	TP53	17p13.1	5/39 (12.8%)	3/16 (18.8%)	2/23 (8.7%)	0.385[1]	0.684
	HER2	17q12	18/39 (46.2%)	5/16 (31.3%)	13/23 (56.5%)	0.192[1]	0.512
	ZNF217	20q13.2	21/39 (53.8%)	7/16 (43.8%)	14/23 (60.9%)	0.342[1]	0.684
**Losses**	Gene	Gene location	All Samples	Diploid Samples	Aneuploid Samples	p-value before MTC	p-value after MTC
	COX2	1p31.1	0/39 (0%)	0/16 (0%)	0/23 (0%)	1[1]	1.000
	DBC2	8p21.3	23/39 (59.0%)	7/16 (43.8%)	16/23 (69.6%)	0.185[1]	0.512
	MYC	8q24.21	5/39 (12.8%)	1/16 (6.3%)	4/23 (17.4%)	0.631[1]	0.830
	CCND1	11q13.3	5/39 (12.8%)	0/16 (0%)	5/23 (21.7%)	0.066[1]	0.344
	CDH1	16q22.1	29/39 (74.4%)	11/16 (68.8%)	18/23 (78.3%)	0.711[1]	0.830
	TP53	17p13.1	27/39 (69.2%)	8/16 (50%)	19/23 (82.6%)	0.041[1]	0.328
	HER2	17q12	8/39 (20.5%)	3/16 (18.8%)	5/23 (21.7%)	1[1]	1.000
	ZNF217	20q13.2	3/39 (7.7%)	0/16 (0%)	3/23 (13.0%)	0.255[1]	0.583
**Ratio**	Ratio gains per sample		3.10	2.6	3.43	0.096[2]	0.096
	Ratio losses per sample		2.56	1.9	3.04	0.013[2]	**0.020**
	Ratio CNA per sample		5.67	4.50	6.48	0.0004[2]	**0.0012**

[1] Fisher exact test; [2] Student t test

CNA, copy number alterations; MTC, multiple test correction.

Supplemental Table S7. MiFISH results showing copy number gains, losses and alterations per gene marker comprising the whole breast cancer cohort (n=39) listed for the group "samples with a low instability index versus samples with a high instability index" with corresponding p-values. MiFISH gene markers are sorted by chromosomal order. If a gain/ loss of signal counts in one of the analyzed miFISH gene markers occured in ≥15% of all nuclei in the respective sample, a gain/loss of copy number was registered accordingly in the tables.

**Table S7.**			**Low versus high Instability Index Samples**			**p-values**	
	Gene	Gene location	All Samples	Low Instability Index Samples	High Instability Index Samples	p-value before MTC	p-value after MTC
**Gains**	*COX2*	1p31.1	28/39 (71.8%)	12/20 (60%)	16/19 (84.2%)	0.163[1]	0.393
	DBC2	8p21.3	9/39 (23.1%)	5/20(25%)	4/19 (21.1%)	1[1]	1
	MYC	8q24.21	27/39 (69.2%)	11/20 (55%)	16/19 (84.2%)	0.089[1]	0.356
	CCND1	11q13.3	11/39 (28.2%)	2/20 (10%)	9/19 (47.4%)	0.012[1]	0.096
	CDH1	16q22.1	2/39 (5.1%)	0/20 (0%)	2/19 (10.5%)	0.219[1]	0.438
	TP53	17p13.1	5/39 (12.8%)	1/20 (5%)	4/19 (21.1%)	0.172[1]	0.393
	HER2	17q12	18/39 (46.2%)	5/20 (25%)	13/19 (68.4%)	0.01[1]	0.096
	ZNF217	20q13.2	21/39 (53.8%)	7/20 (35%)	14/19 (73.3%)	0.031[1]	0.165
**Losses**	Gene	Gene location	All Samples	Low Instability Index Samples	High Instability Index Samples	p-value before MTC	p-value after MTC
	COX2	1p31.1	0/39 (0%)	0/20 (0%)	0/20 (0%)	1[1]	1
	DBC2	8p21.3	23/39 (59.0%)	9/20 (45%)	14/20 (73.7%)	0.117[1]	0.374
	MYC	8q24.21	5/39 (12.8%)	3/20 (15%)	2/20 (10.5%)	1[1]	1
	CCND1	11q13.3	5/39 (12.8%)	2/20 (10%)	3/20 (15.8%)	0.654[1]	0.872
	CDH1	16q22.1	29/39 (74.4%)	16/20 (80%)	13/20 (68.4%)	0.473[1]	0.757
	TP53	17p13.1	27/39 (69.2%)	12/20 (60%)	15/20 (79.0%)	0.311[1]	0.553
	HER2	17q12	8/39 (20.5%)	4/20 (20%)	4/20 (21.1%)	1[1]	1
	ZNF217	20q13.2	3/39 (7.7%)	1/20 (5%)	2/20 (10.5%)	0.596[1]	0.867
**Ratio**	Ratio gains per sample		3.10	2.15	4.11	<0.0001[2]	**<0.0001**
	Ratio losses per sample		2.56	2.35	2.79	0.350[2]	0.350
	Ratio CNA per sample		5.67	4.50	6.89	<0.0001[2]	**<0.0001**

[1] Fisher exact test; [2] Student t test

CNA, copy number alterations; MTC, multiple test correction.
