# Supplementary material for: Single Cell Genetic Profiling of Tumors of Breast Cancer Patients Aged 50 Years and Older Reveals Enormous Intratumor Heterogeneity Independent of Individual Prognosis
Source: Cancers (Basel). 2021 Jul 5;13(13):3366. doi: 10.3390/cancers13133366 (PMC8267950; doi:10.3390/cancers13133366)
Supplement: Supplementary file 1 [file cancers-13-03366-s001.zip › cancers-1245840-SI/Supplementary_Files/Supplemental Tables/S8 Supplemental Table.pdf]

**Supplemental Table S8.** Mutual exclusivity and co-occurrence analysis of copy number alterations of eight breast-cancer related genes (miFISH results of gene-probes *COX2, DBC2, MYC, CCND1, CDH1, TP53, HER2, ZNF217*) and mutations in the two most frequently mutated genes *TP53* and *PIK3CA* (NGS results) with corresponding p- and q-values using the Mutual Exclusivity Modules in Cancer (MeMo) algorithm as described in Materials and Methods.

| **Genes** | | **Mutual exclusivity/ Co-occurence analysis** | | | | | **p/q-Values** | | **Tendency** | |
|---|---|---|---|---|---|---|---|---|---|---|
| Gene A | Gene B | Neither | A Not B | B Not A | Both | Log2 Odds Ratio | p-Value | q-Value | Co-occurence/ Mutual exclusivity | |
| *DBC2* | *MYC* | 5 | 2 | 2 | 30 | >3 | <0.001 | 0.023 | Co-occurrence | **significant** |
| *PIK3CA* | *HER2* | 5 | 9 | 22 | 3 | <-3 | 0.001 | 0.023 | Mutual exclusivity | |
| *DBC2* | *HER2* | 6 | 8 | 1 | 24 | >3 | 0.005 | 0.042 | Co-occurrence | |
| *TP53* | *HER2* | 6 | 8 | 1 | 24 | >3 | 0.005 | 0.042 | Co-occurrence | |
| *PIK3CA* | *CCND1* | 12 | 11 | 15 | 1 | <-3 | 0.006 | 0.042 | Mutual exclusivity | |
| *PIK3CA* | *CDH1* | 9 | 0 | 18 | 12 | >3 | 0.022 | 0.133 | Co-occurrence | **not significant** |
| *CCND1* | *HER2* | 11 | 3 | 12 | 13 | 1.99 | 0.062 | 0.320 | Co-occurrence | |
| *TP53* | *ZNF217* | 5 | 11 | 2 | 21 | 2.255 | 0.085 | 0.338 | Co-occurrence | |
| *CCND1* | *ZNF217* | 12 | 4 | 11 | 12 | 1.71 | 0.085 | 0.338 | Co-occurrence | |
| *DBC2* | *TP53* | 3 | 4 | 4 | 28 | 2.392 | 0.094 | 0.338 | Co-occurrence | |
| *CCND1* | *TP53* | 6 | 1 | 17 | 15 | 2.404 | 0.121 | 0.396 | Co-occurrence | |
| *MYC* | *HER2* | 4 | 10 | 3 | 22 | 1.553 | 0.194 | 0.537 | Co-occurrence | |
| *COX2* | *CDH1* | 1 | 8 | 10 | 20 | -2 | 0.194 | 0.537 | Mutual exclusivity | |
| *COX2* | *CCND1* | 8 | 15 | 3 | 13 | 1.209 | 0.234 | 0.577 | Co-occurrence | |
| *CDH1* | *HER2* | 2 | 12 | 7 | 18 | -1.222 | 0.288 | 0.577 | Mutual exclusivity | |
| *MYC* | *ZNF217* | 4 | 12 | 3 | 20 | 1.152 | 0.294 | 0.577 | Co-occurrence | |
| *COX2* | *MYC* | 3 | 4 | 8 | 24 | 1.17 | 0.302 | 0.577 | Co-occurrence | |
| *HER2* | *ZNF217* | 7 | 9 | 7 | 16 | 0.83 | 0.303 | 0.577 | Co-occurrence | |
| *COX2* | *HER2* | 5 | 9 | 6 | 19 | 0.815 | 0.337 | 0.577 | Co-occurrence | |
| *PIK3CA* | *ZNF217* | 10 | 6 | 17 | 6 | -0.766 | 0.340 | 0.577 | Mutual exclusivity | |
| *PIK3CA* | *DBC2* | 4 | 3 | 23 | 9 | -0.939 | 0.365 | 0.577 | Mutual exclusivity | |
| *PIK3CA* | *TP53* | 4 | 3 | 23 | 9 | -0.939 | 0.365 | 0.577 | Mutual exclusivity | |
| *MYC* | *TP53* | 2 | 5 | 5 | 27 | 1.111 | 0.369 | 0.577 | Co-occurrence | |
| *COX2* | *PIK3CA* | 7 | 20 | 4 | 8 | -0.515 | 0.456 | 0.654 | Mutual exclusivity | |
| *CDH1* | *TP53* | 1 | 6 | 8 | 24 | -1 | 0.480 | 0.654 | Mutual exclusivity | |
| *COX2* | *ZNF217* | 5 | 11 | 6 | 17 | 0.365 | 0.500 | 0.654 | Co-occurrence | |
| *DBC2* | *CDH1* | 2 | 7 | 5 | 25 | 0.515 | 0.520 | 0.654 | Co-occurrence | |
| *MYC* | *CDH1* | 2 | 7 | 5 | 25 | 0.515 | 0.520 | 0.654 | Co-occurrence | |
| *CCND1* | *CDH1* | 5 | 4 | 18 | 12 | -0.263 | 0.554 | 0.654 | Mutual exclusivity | |
| *CDH1* | *ZNF217* | 4 | 12 | 5 | 18 | 0.263 | 0.554 | 0.654 | Co-occurrence | |
| *DBC2* | *CCND1* | 4 | 19 | 3 | 13 | -0.132 | 0.617 | 0.654 | Mutual exclusivity | |
| *MYC* | *CCND1* | 4 | 19 | 3 | 13 | -0.132 | 0.617 | 0.654 | Mutual exclusivity | |
| *DBC2* | *ZNF217* | 3 | 13 | 4 | 19 | 0.132 | 0.617 | 0.654 | Co-occurrence | |
| *PIK3CA* | *MYC* | 5 | 2 | 22 | 10 | 0.184 | 0.635 | 0.654 | Co-occurrence | |
| *COX2* | *DBC2* | 2 | 5 | 9 | 23 | 0.032 | 0.654 | 0.654 | Co-occurrence | |
| *COX2* | *TP53* | 2 | 5 | 9 | 23 | 0.032 | 0.654 | 0.654 | Co-occurrence | |
