# Supplementary material for: Single Cell Genetic Profiling of Tumors of Breast Cancer Patients Aged 50 Years and Older Reveals Enormous Intratumor Heterogeneity Independent of Individual Prognosis
Source: Cancers (Basel). 2021 Jul 5;13(13):3366. doi: 10.3390/cancers13133366 (PMC8267950; doi:10.3390/cancers13133366)
Supplement: Supplementary file 1 [file cancers-13-03366-s001.zip › cancers-1245840-SI/Supplementary_Files/Supplemental Tables/S9 Supplemental Table.pdf]

**Supplemental Table S9.** Genes included in OncoVar assay (n=563).

| OncoVar genes | | | | | | | | | |
|---|---|---|---|---|---|---|---|---|---|
| ABL1 | BLM | CTNNA1 | FANCG | HIF1A | LCK | MYH11 | PIK3CG | RUNX1T1 | TET2 |
| ABL2 | BLNK | CTNNB1 | FANCI | HIST1H3B | LDLR | MYH7 | PIK3R1 | RYR1 | TFE3 |
| ACN9 | BMPR1A | CUL2 | FANCL | HLF | LIFR | MYH9 | PIK3R2 | RYR2 | TGFBR1 |
| ACTA2 | BRAF | CUL3 | FANCM | HNF1A | LMNA | MYL2 | PIM1 | SAMD9 | TGFBR2 |
| ACTC1 | BRCA1 | CYLD | FAS | HOOK3 | LPHN3 | MYL3 | PKHD1 | SBDS | TGM7 |
| ACVR1 | BRCA2 | CYP2C19 | FBN1 | HOXA3 | LPP | MYLK | PKP2 | SCN5A | THBS1 |
| ACVR1B | BRD3 | CYP2D6 | FBXO11 | HRAS | LRP1B | NBN | PLAG1 | SDHA | TIAF1 |
| ACVR2A | BRIP1 | DAXX | FBXW7 | HSP90AA1 | LTF | NCOA1 | PLCG1 | SDHAF1 | TIMP3 |
| ACVRL1 | BTK | DCC | FGF10 | HSP90AB1 | LTK | NCOA2 | PLEKHG5 | SDHAF2 | TLR4 |
| ADAMTS20 | BUB1B | DDB2 | FGF14 | ICK | MAF | NCOA3 | PML | SDHB | TLX1 |
| AFF1 | C11ORF30 | DDIT3 | FGF19 | IDH1 | MAFB | NCOA4 | PMS1 | SDHC | TMEM127 |
| AFF3 | CACNA1S | DDR2 | FGF23 | IDH2 | MAGEA1 | NF1 | PMS2 | SDHD | TMEM43 |
| AKAP9 | CALR | DEK | FGF3 | IFITM1 | MAGI1 | NF2 | PNP | SEPT9 | TNFAIP3 |
| AKT1 | CARD11 | DICER1 | FGF4 | IFITM3 | MAGOH | NFE2L2 | POLD1 | SETD2 | TNFRSF14 |
| AKT2 | CASC5 | DNMT3A | FGF6 | IGF1R | MALT1 | NFKB1 | POLE | SF3B1 | TNK2 |
| AKT3 | CBFB | DOT1L | FGFR1 | IGF2 | MAML1 | NFKB2 | POLH | SGK1 | TNNI3 |
| ALK | CBL | DPYD | FGFR2 | IGF2R | MAML2 | NFKBIA | POT1 | SH2B3 | TNNT2 |
| AMER1 | CCND1 | DSC2 | FGFR3 | IKBKB | MAP2K1 | NIN | POU5F1 | SH2D1A | TOP1 |
| APC | CCND2 | DSG2 | FGFR4 | IKBKE | MAP2K2 | NKX2-1 | PPARG | SIRT1 | TP53 |
| APEX1 | CCND3 | DSP | FH | IKZF1 | MAP2K4 | NLRP1 | PPP2R1A | SMAD2 | TPM1 |
| APOB | CCNE1 | DST | FLCN | IL2 | MAP3K1 | NOTCH1 | PRDM1 | SMAD3 | TPR |
| AR | CD274 | EGFR | FLI1 | IL21R | MAP3K7 | NOTCH2 | PRKAG2 | SMAD4 | TRAF7 |
| ARAF | CD44 | EML4 | FLT1 | IL6 | MAPK1 | NOTCH3 | PRKAR1A | SMARCA4 | TRIM24 |
| ARFRP1 | CD79A | EP300 | FLT3 | IL6ST | MAPK8 | NOTCH4 | PRKDC | SMARCB1 | TRIM33 |
| ARID1A | CD79B | EP400 | FLT4 | IL7R | MARK1 | NPM1 | PRSS1 | SMO | TRIP11 |
| ARID1B | CDC73 | EPHA3 | FN1 | ING4 | MARK4 | NRAS | PSIP1 | SMUG1 | TRRAP |
| ARID2 | CDH1 | EPHA5 | FNIP1 | INHBA | MAX | NSD1 | PTCH1 | SOCS1 | TSC1 |
| ARNT | CDH11 | EPHA6 | FNIP2 | IRF4 | MBD1 | NTRK1 | PTEN | SOX10 | TSC2 |
| ASXL1 | CDH2 | EPHA7 | FOXL2 | IRS2 | MBIP | NTRK2 | PTGS2 | SOX11 | TSHR |
| ATF1 | CDH20 | EPHB1 | FOXO1 | ITGA10 | MCL1 | NTRK3 | PTPN11 | SOX2 | U2AF1 |
| ATM | CDH5 | EPHB4 | FOXO3 | ITGA9 | MDM2 | NUMA1 | PTPRD | SPEN | UBA1 |
| ATR | CDK12 | EPHB6 | FOXP1 | ITGB2 | MDM4 | NUP214 | PTPRT | SPOP | UBR5 |
| ATRX | CDK4 | ERBB2 | FOXP4 | ITGB3 | MED12 | NUP93 | RAC1 | SRC | UGT1A1 |
| AURKA | CDK6 | ERBB3 | FZR1 | JAK1 | MEF2B | NUP98 | RAD50 | SRSF2 | USP9X |
| AURKB | CDK8 | ERBB4 | G6PD | JAK2 | MEN1 | PAK3 | RAD51 | SSX1 | VHL |
| AURKC | CDKN1B | ERCC1 | GAS6 | JAK3 | MET | PALB2 | RAD51C | STAG1 | WAS |
| AXIN1 | CDKN2A | ERCC2 | GATA1 | JUN | MGMT | PARP1 | RAF1 | STAG2 | WHSC1 |
| AXIN2 | CDKN2B | ERCC3 | GATA2 | KAT6A | MITF | PAX3 | RALGDS | STAT3 | WISP3 |
| AXL | CDKN2C | ERCC4 | GATA3 | KAT6B | MLH1 | PAX5 | RARA | STAT4 | WRN |
| BAI3 | CEBPA | ERCC5 | GDNF | KCNH2 | MLLT10 | PAX7 | RB1 | STK11 | WT1 |
| BAP1 | CHEK1 | ERG | GID4 | KCNJ5 | MMP2 | PAX8 | RECQL4 | STK36 | XPA |
| BARD1 | CHEK2 | ESR1 | GLA | KCNQ1 | MN1 | PBRM1 | REL | SUFU | XPC |
| BCL10 | CIC | ETS1 | GNA11 | KDM5A | MPL | PBX1 | RET | SYK | XPO1 |
| BCL11A | CKS1B | ETV1 | GNA13 | KDM5C | MRE11A | PCSK9 | RHEB | SYNE1 | XRCC1 |
| BCL11B | CMPK1 | ETV4 | GNAQ | KDM6A | MSH2 | PDE4DIP | RHOA | TAF1 | XRCC2 |
| BCL2 | COL1A1 | ETV5 | GNAS | KDR | MSH6 | PDGFB | RHOH | TAF1L | ZNF217 |
| BCL2L1 | COL3A1 | EWSR1 | GPC3 | KEAP1 | MTOR | PDGFRA | RICTOR | TAL1 | ZNF384 |
| BCL2L2 | CRBN | EXT1 | GPR124 | KIF1B | MTR | PDGFRB | RNASEL | TBX22 | ZNF521 |
| BCL3 | CREB1 | EXT2 | GRIN2A | KIT | MTRR | PDK1 | RNF2 | TCEB1 | ZNF703 |
| BCL6 | CREBBP | EZH2 | GRM8 | KLF6 | MUC1 | PER1 | RNF213 | TCEB2 | ZRSR2 |
| BCL9 | CRKL | FAM46C | GSK3B | KLHL6 | MUTYH | PGAP3 | RNF43 | TCF12 | |
| BCOR | CRLF2 | FANCA | GTF2I | KMT2A | MYB | PGR | ROS1 | TCF3 | |
| BCORL1 | CRTC1 | FANCB | GUCY1A2 | KMT2B | MYBPC3 | PHOX2B | RPS6KA2 | TCF7L1 | |
| BCR | CSF1R | FANCC | H3F3A | KMT2C | MYC | PIK3C2B | RPS6KB1 | TCF7L2 | |
| BIRC2 | CSMD3 | FANCD2 | H3F3B | KNSTRN | MYCL | PIK3CA | RPTOR | TCL1A | |
| BIRC3 | CSNK2A1 | FANCE | HCAR1 | KRAS | MYCN | PIK3CB | RRM1 | TERT | |
| BIRC5 | CTCF | FANCF | HGF | LAMP1 | MYD88 | PIK3CD | RUNX1 | TET1 | |
