# Supplementary figures and images for: Single Cell Genetic Profiling of Tumors of Breast Cancer Patients Aged 50 Years and Older Reveals Enormous Intratumor Heterogeneity Independent of Individual Prognosis
Source: Cancers (Basel). 2021 Jul 5;13(13):3366. doi: 10.3390/cancers13133366 (PMC8267950; doi:10.3390/cancers13133366)

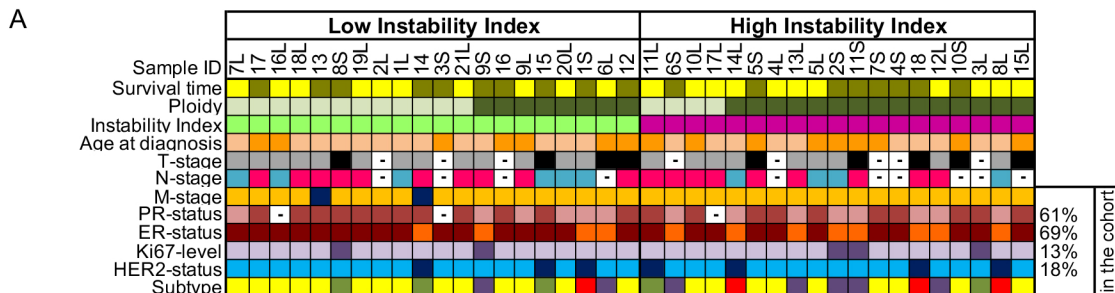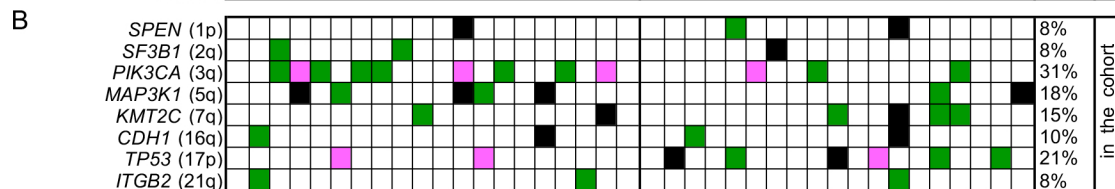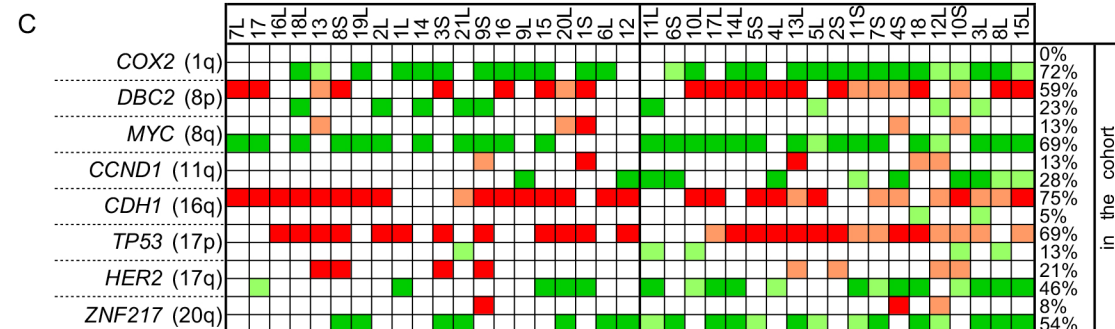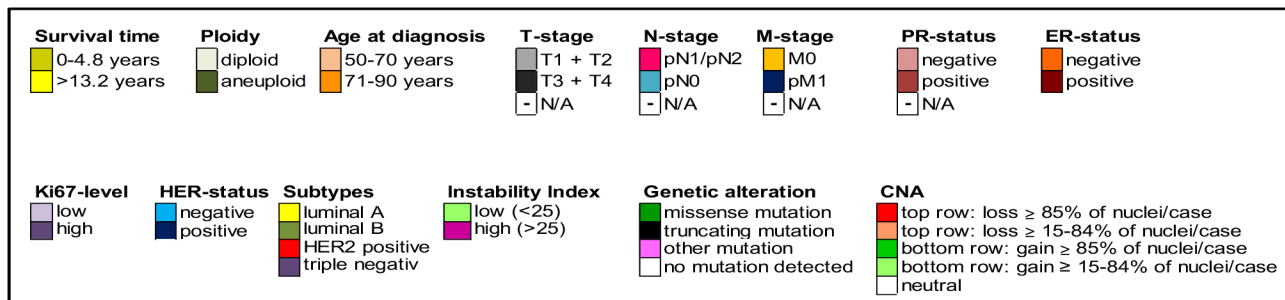

Supplemental Figure S4

Supplement: Supplementary file 1 [file cancers-13-03366-s001.zip › cancers-1245840-SI/Supplementary_Files/Supplemental Figures/Supplemental Figure S4.pdf]

A

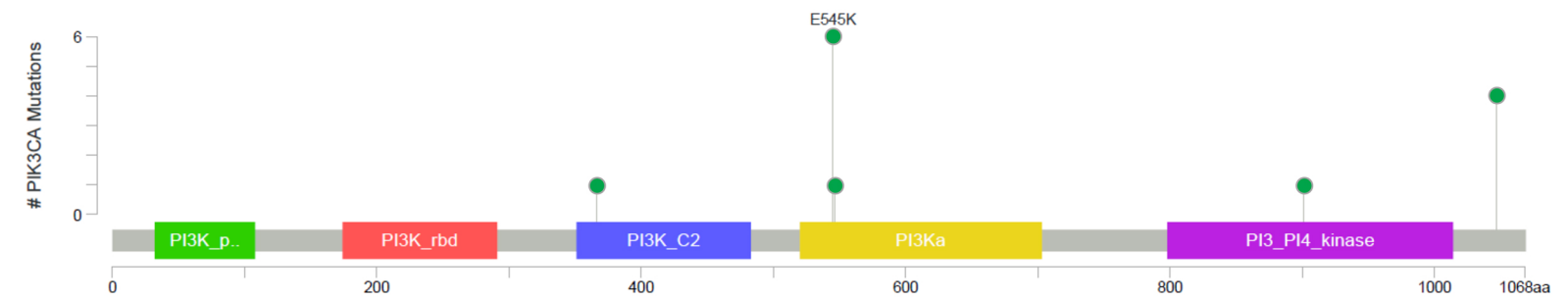

B

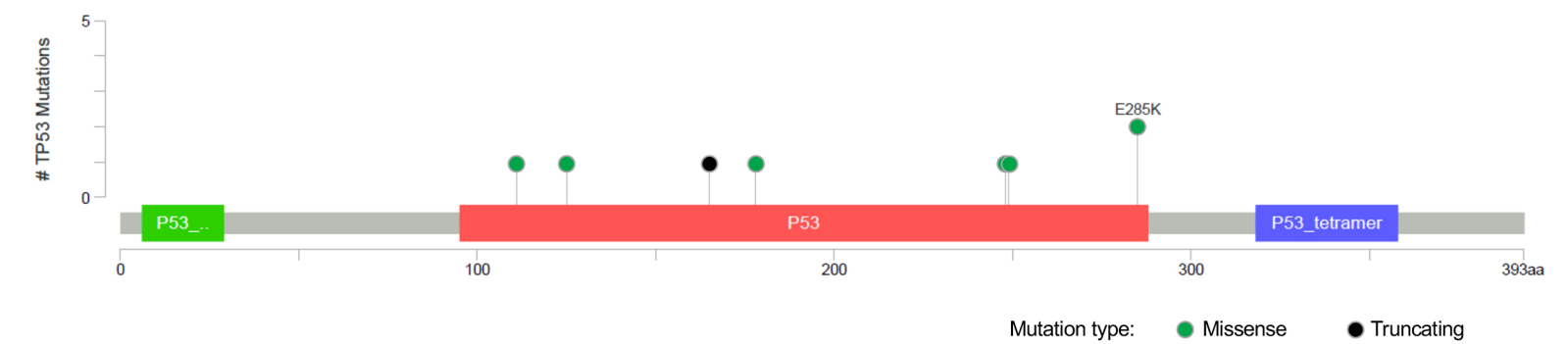

Supplemental Figure S5

Supplement: Supplementary file 1 [file cancers-13-03366-s001.zip › cancers-1245840-SI/Supplementary_Files/Supplemental Figures/Supplemental Figure S5.pdf]

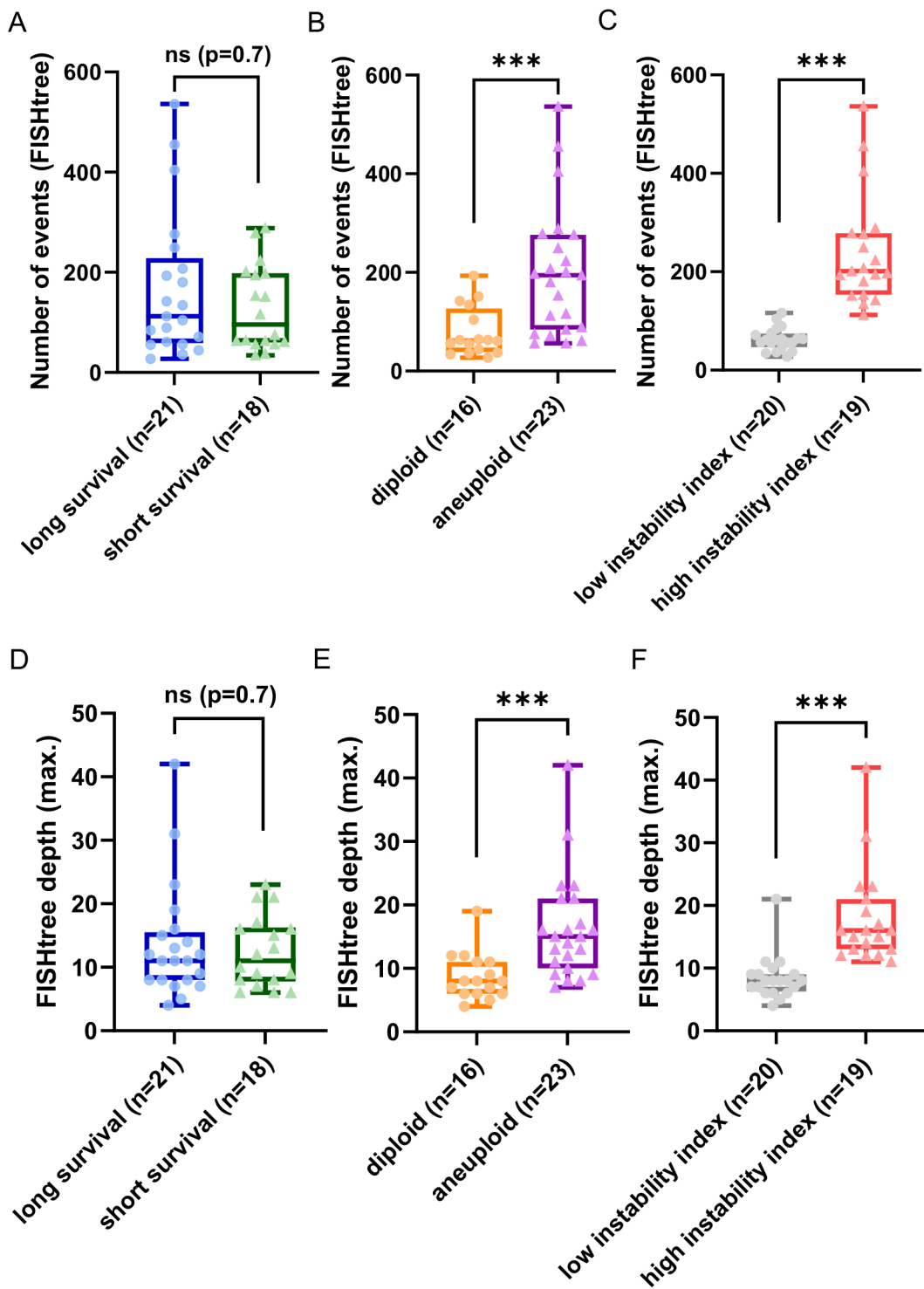

Supplemental Figure S7

Supplement: Supplementary file 1 [file cancers-13-03366-s001.zip › cancers-1245840-SI/Supplementary_Files/Supplemental Figures/Supplemental Figure S7.pdf]
